# Parasitic plants in Europe: ecological niches and spatial patterns

**DOI:** 10.1111/plb.70099

**Published:** 2025-09-18

**Authors:** N. Fahs, I. Axmanová, J.‐C. Svenning, J. Padullés Cubino, I. Biurrun, S. Boch, J. A. Campos, A. Čarni, J. Dengler, E. Garbolino, T. Heinken, K. Knotková, J. Těšitel

**Affiliations:** ^1^ Department of Botany and Zoology, Faculty of Science Masaryk University Brno Czechia; ^2^ Center for Ecological Dynamics in a Novel Biosphere (ECONOVO) Aarhus University Aarhus Denmark; ^3^ Campus de Bellaterra (UAB) Edifici C, Center for Ecological Research and Forestry Applications Cerdanyola del Vallès Spain; ^4^ Unit of Botany, Edifici C, Campus de Bellaterra (UAB) Autonomous University of Barcelona Cerdanyola del Vallès Spain; ^5^ Department of Plant Biology and Ecology University of the Basque Country UPV/EHU Leioa Spain; ^6^ Swiss Federal Institute for Forest, Snow and Landscape Research WSL Birmensdorf Switzerland; ^7^ Jovan Hadži Institute of Biology Research Centre of the Slovenian Academy of Sciences and Arts Ljubljana Slovenia; ^8^ School for Viticulture and Enology University of Nova Gorica Nova Gorica Slovenia; ^9^ Vegetation Ecology Research Group, Institute of Natural Resource Sciences (IUNR) ZHAW University of Applied Sciences Wädenswil Switzerland; ^10^ Plant Ecology, Bayreuth Center of Ecology and Ecological Research (BayCEER) University of Bayreuth Bayreuth Germany; ^11^ Higher Institute for Environmental Engineering and Management (ISIGE) Fontainebleau France; ^12^ General Botany, Institute for Biochemistry and Biology University of Potsdam Potsdam Germany

**Keywords:** CHELSA Bioclim, ecological niche, EUNIS classification, hemiparasite, parasitic plant, parasitic vine, root parasite

## Abstract

- Parasitic plants inhabit a wide range of ecosystems worldwide, where they may have critical roles as “ecosystem engineers”. We examined the ecology of parasitic plants in Europe. We aimed to identify habitat preferences, spatial distribution, and environmental drivers of parasitic plant functional types: euphytoid hemiparasites, obligate root parasites, and parasitic vines, and assess individual species' ecological niches.
- We analysed 244 parasitic plant species in a dataset of 819,452 vegetation plots across European natural vegetation. We used a boosted regression tree model to assess the effects of macro‐climate, topography, and habitat descriptors (open, wet, saline) on the distribution of parasitic plant functional types. We analysed their distribution along the gradients of ecological indicator values. Finally, we determined the niches of individual species along all the environmental gradients.
- Parasitic plants occur across Europe and in nearly all habitats. Euphytoid hemiparasites (173 species) are most abundant in colder environments with moderate to high precipitation and low precipitation seasonality. In contrast, obligate root parasites (52 species) and parasitic vines (12 species) are primarily associated with warm‐temperate to Mediterranean dry climates. All three functional types prefer nutrient‐poor to moderately rich conditions. Some species diverge from the trend of their functional type.
- The spatial distribution and niches of parasitic plant functional types correspond to their fundamental physiological properties, including mode of resource acquisition and level of photosynthesis. Euphytoid hemiparasites are likely to be negatively affected by climate warming, while obligate root parasites and parasitic vines might benefit from future warmer and drier climates.

## Graphical abstract

Parasitic plant functional types in Europe exhibit different distribution patterns in terms of habitats, geography, and major environmental gradients, reflecting their physiological properties.

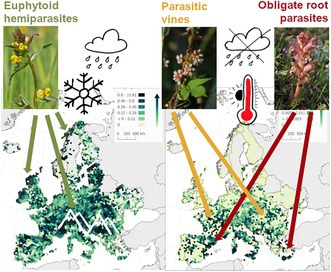

## INTRODUCTION

Parasitic plants represent a diverse group of angiosperms, accounting for 1.6% of all known angiosperm species (Nickrent 2020). These plants obtain some or all of their essential nutrients from other plants, thereby escaping some of the ecological constraints associated with resource deficiencies. Using a specialised organ, the haustorium, they attach to another plant's vascular bundle either at the root or stem (Teixeira‐Costa 2021). The variability of parasitic plant strategies and uneven distribution of diversity in both the phylogenetic and biogeographic sense (Těšitel 2016; Nickrent 2020; Teixeira‐Costa & Davis 2021) makes it impossible to draw general conclusions on the adaptive value of plant parasitism, which could be derived from, e.g., phylogenetically informed regression. Parasitic plants, however, play a significant role in the ecology of terrestrial ecosystems. For numerous parasitic plant species, a series of (mostly local) case studies demonstrated their ability to facilitate nutrient cycling, manipulate productivity, structure plant communities, and eventually increase diversity (e.g., Pennings & Callaway 1996; Davies *et al*. 1997; Quested 2008; Fibich *et al*. 2017; Heer *et al*. 2018; Těšitel *et al*. 2021). Despite their ecological relevance, large‐scale studies assessing the ecological niches of parasitic plants are rare and often only regional (Ter Borg 1985; Giannini *et al*. 2011; Těšitel *et al*. 2015; O'Neill & Rana 2016; Zhang *et al*. 2018). The establishment of vegetation databases, such as the European Vegetation Archive (EVA; Chytrý *et al*. 2016), has facilitated several broad‐scale analyses of functional groups and vegetation patterns (Večeřa *et al*. 2021; Midolo *et al*. 2024). However, a comprehensive study of the ecological niches of parasitic plants across European habitats is lacking.

Parasitic plants display a wide range of parasitic resource acquisition strategies, life forms and cycles, and host‐attachment mechanisms (Heide‐Jørgensen 2008). These facets of parasitic plant functioning imply different adaptations and constraints, which translate into fitness under different environmental conditions. Functional classifications of parasitic plants were therefore introduced to better understand their biology (Heide‐Jørgensen 2013; Těšitel 2016; Teixeira‐Costa & Davis 2021). Here, we adopted the functional classification of Teixeira‐Costa & Davis (2021), slightly modified for the European flora (Těšitel *et al*. 2024 in Chytrý *et al*. 2024):

- Euphytoid hemiparasites: these are green parasitic plants that retain photosynthetic capacity. Once attached to the host below the ground, they acquire practically all mineral nutrients and water from their host's xylem (Irving & Cameron 2009). Euphytoid hemiparasites rely on their own photosynthesis for most organic carbon, making them dependent on light availability and thus susceptible to aboveground competition (Matthies 1995; Mudrák & Lepš 2010). Nonetheless, parasitism often reduces the competitive ability of the hosts, which consequently restricts the effect of competition on the hemiparasites (Matthies 1995; Těšitel *et al*. 2015). Genera common in Europe include *Euphrasia*, *Melampyrum*, *Odontites, Rhinanthus, Pedicularis* and *Thesium*.
- Obligate root parasites: these plants cannot survive without a host during their early development, relying entirely on their seed reserves while locating a suitable host (Teixeira‐Costa & Davis 2021). Unlike euphytoid hemiparasites, they are mostly non‐photosynthetic, obtaining resources from both the xylem and phloem of their host, and do not depend on light availability for survival. From this group, the most common species in Europe belong to the genera *Orobanche* and *Lathraea*. Endoparasites, the most reduced form of parasitic plants (Teixeira‐Costa & Davis 2021), are included in this category in the present study, because the only represented species, *Cytinus hypocystis*, grows endophytically within the roots of its host, and thus is an obligate parasite (de Vega *et al*. 2023).
- Parasitic vines: this group comprises two evolutionarily distinct genera, *Cuscuta* and *Cassytha*, with only *Cuscuta* found in Europe. They germinate on the ground and grow independently until attaching to a host stem (Kuijt 1969). After successfully attaching to the host's xylem and phloem, they no longer need their connection to the ground, which then withers (Verdcourt 1948; Heide‐Jørgensen 2008). *Cuscuta* exhibits only rudimentary photosynthesis, which, however, still plays an important role in seed production and establishing its first connection to the host (Tĕšitel *et al*. 2018).
- Mistletoes (only partially considered in this study): these are photosynthesising woody angiosperms that germinate on the branches of their hosts (mainly trees), and connect to the host xylem (Teixeira‐Costa & Davis 2021). By growing in the canopy, mistletoes successfully elevate themselves from light restriction in the understory (Vidal‐Russell & Nickrent 2008). Only a few mistletoe species occur in Europe (e.g., species in the genera *Viscum* and *Loranthus*), representing a tiny fraction of their global diversity, which is concentrated in tropical and former Gondwanan regions, including those outside the tropics (Heide‐Jørgensen 2008).

In this study, we aim to identify spatial patterns and environmental determinants of functional types of parasitic plants, as well as their ecological niches within natural and semi‐natural vegetation across Europe. Through both community‐level and species‐level analyses, our specific objectives are to: (I) characterise the spatial patterns and habitat associations of different parasitic plant functional types; (II) identify the key environmental drivers shaping these spatial patterns; and (III) define the ecological niches of individual parasitic species.

## MATERIAL AND METHODS

### Vegetation data

The study is based on an extensive dataset of 1,122,281 vegetation plot records covering various habitats across Europe, provided by the European Vegetation Archive (EVA; Chytrý *et al*. 2016; project number: 137; date of download: 26‐11‐2021). The initial dataset was extracted from EVA based on specific selection criteria: (i) presence of georeference (location uncertainty up to 10 km), (ii) presence of cover‐abundance values, and (iii) excluding aquatic vegetation. Additional filtering was performed to focus on the countries of interest (see Appendix S1). The database includes plots of varying sizes, with many lacking plot size information. We chose to include all plots in our analyses so as not to lose relevant data. The variation in plot size is not expected to affect analyses using Ecological indicator values for Europe (EIVE). To minimise the influence of plot size on all the other analyses, we focused on relative cover values and additionally used plot size as a variable in the boosted regression tree analysis. Plot sizes ranged from 1 to 1000 m^2^ (final plot size ranges for each habitat type at the fine (EUNIS‐level 3) and broad (EUNIS‐level 1) levels are detailed in Appendix S2).

To minimise potential spatial pseudoreplication, we implemented geographic stratification, considering both the spatial distance and similarity of species composition. Specifically, from each pair of plots within 1 km distance and compositional similarity >0.8 (Bray–Curtis dissimilarity weighted by cover values), only one plot was randomly selected.

All plots were classified into EUNIS (European Nature Information System) habitat types using the EUNIS‐ESy expert system (Chytrý *et al*. 2020). The first hierarchical level corresponds to broad‐level habitat types: grasslands (R), forests (T), coastal sand and cliff habitats (N), coastal saltmarshes (M), wetlands (Q), heathlands, scrub and tundra (S), inland sparsely vegetated habitats (U) and anthropogenic habitats (V). The subsequent levels provide more precise habitat definitions, such as “Dry grasslands” at the medium level and “Perennial rocky grassland of Central and Southeast Europe” at the fine level. We excluded all strongly synanthropic habitats (class V, and several types of forest plantation of non‐native trees in other classes) from our analysis, as our focus was on natural and semi‐natural vegetation. Additionally, we excluded transitional habitat types of forest clearings (R57), recently felled areas (T41‐43) and plantations and forests of non‐native trees (T1H‐K, T29, T1A, T3L‐T3N, T3M, T4). An overview of plot numbers assigned to the specific EUNIS habitat types on different levels and their use in different analyses is provided in Table S3. The final dataset included 819,452 vegetation plots. Different subsets were used for the different analyses, based on data availability. For more information see Table S3.

### Species nomenclature and parasitic plant functional types

We removed all records of non‐vascular species and entries that were not identified to at least the family level. We standardised species names and abbreviations according to the Euro+Med PlantBase checklist for vascular plants (Euro+Med 2023). Intraspecific taxa were merged at the species level, and species were merged into aggregates, as defined in the EUNIS‐ESy expert system classification (Chytrý *et al*. 2020). Given the inconsistent use of layers (tree/shrub/herb) in available vegetation records, we did not consider them in our analysis. Instead, we aggregated records from different layers within a plot by summing the individual species cover values, accounting for their possible overlap using the Jennings–Fischer formula (Jennings *et al*. 2009; Fischer 2015).

We assigned each taxon a category according to Těšitel *et al*. (2024): euphytoid hemiparasite, obligate root parasite (here: including one endophyte species, see above), parasitic vine, mistletoe, autotroph. Species records identified only to the family level were retained for all non‐parasitic plants (families with no parasitic species), while records of parasitic plants were all identified at least to the genus level. We kept these for analysing the distribution of parasitic plant functional types while excluding them from species‐level analyses. We only kept mistletoes for displaying their occurrence in habitat types (Appendix S2) and the species‐pool analysis (Appendix S4), as their representation in our dataset was insufficient and strongly geographically biased. This prevented us from making reasonable inferences from their records.

### Environmental variables

We obtained climate data from CHELSA Bioclim (Karger *et al*. 2017, 2018) and BIOCLIM+ (Brun *et al*. 2022a, 2022b) at a spatial resolution of 30 arc seconds. To account for topographic variation, we included data on the Terrain Ruggedness Index (TRI) at ~90 m (3 arc seconds) resolution from Geomorpho90m (Amatulli *et al*. 2018). A complete list of the selected variables, together with information on eventual transformation applied to improve the linearity of associations, is provided in Table S3. We first made a preselection within the climate predictor dataset to reduce its dimensionality and remove strongly intercorrelated variables. This was done by sequential selection of candidate predictors based on their ability to explain variability in the remaining part of the climate predictor dataset. When multiple predictors accounted for variation to a similar extent, we prioritised those that best represented conditions during the main growing season for most plant species. For example, we favoured the Mean daily mean air temperatures of the warmest quarter (bio10) over the Mean annual air temperature (bio1). Technically, this was conducted using redundancy analysis (RDA) with stepwise predictor selection. The final selection of predictors explained 95.6% of the total climate data variability (*R* ^2^_adj_). The final selection of climate variables included mean diurnal air temperature range (bio2), temperature seasonality (bio4), mean daily mean air temperatures of the warmest quarter (bio10), annual precipitation amount (bio12), precipitation seasonality (bio15), mean monthly precipitation amount of the warmest quarter (bio18), and mean monthly potential evapotranspiration (pen_penman; for further information see Appendix S5).

To further describe habitat properties, we developed binary descriptors for each fine‐level EUNIS habitat type in three different categories: 1. *Open habitats*: if the habitat is considered open vegetation with merely small shrubs and no prominent shading in the herb layer by trees or tall shrubs; 2. *Wet habitats*: if the habitat is predominantly waterlogged throughout the year or regularly flooded; 3. *Saline habitats*: if at least low constant salinity is expected. An overview of the classification for each habitat type is presented in Table S3.

### Ecological indicator values for Europe

To analyse fine‐scale responses of functional types of parasitic plants to community interactions and processes along environmental gradients, we utilised Ecological Indicator Values for Europe (EIVE) for soil nitrogen, soil moisture, soil reaction, temperature and light (Dengler *et al*. 2023). These values provide empirical indicators (e.g., 0 for low‐soil nitrogen environments to 10 for high‐soil nitrogen environments) and represent fine‐scale ecological site conditions that might not be captured by macro‐environmental variables (following Ellenberg 1974, compiled and rescaled for Europe by Dengler *et al*. 2023). For species aggregates in our dataset that were not provided by Dengler *et al*. (2023), we calculated the mean value for each variable using all species included in these aggregates (according to Chytrý *et al*. 2020) for which data were available. We then computed the unweighted mean EIVE for each plot separately for each functional type of parasite, as well as for all types combined, excluding the species belonging to the focal type in each case.

### Statistical analysis

#### Spatial patterns and habitat associations

The relative cover of each parasitic plant functional type was obtained per plot as the sum of relative cover values of all parasitic species of the respective functional type recorded in the plot, which is each species' individual cover divided by the sum of individual species cover values in the plot. The relative cover is indicative of the proportions of parasitic plants in the community and allows the comparison of their relative abundances between high‐ and low‐density vegetation types.

Using QGIS (QGIS Desktop 3.32.1; QGIS Association 2024), we defined a hexagonal grid with cells 50 km in latitude across the study area, with each grid cell covering an area of 2165 km^2^. We then calculated the mean relative cover for each functional type of parasite for each grid cell based on the plot data. Colour classes for the resulting maps were defined using the quantile classification method implemented in QGIS (excluding grid cells with 0% mean relative cover from the class calculation). Additionally, we prepared habitat‐specific maps based on the broad‐level EUNIS habitat types for each functional type following the same methods (Appendix S6) and maps representing the distribution of selected environmental variables (Appendix S7).

#### Environmental drivers

We modelled the effect of environmental variables (climate, topography) and habitat descriptors (open, wet, saline) on the plot‐level relative cover of each parasitic plant functional type using boosted regression trees (BRTs). BRTs are effective for modelling large‐scale vegetation data, as they combine regression trees and boosting algorithms to fit complex, non‐linear relationships using various types of response variables (De'ath 2007; Viana *et al*. 2022). We included plot size (square‐root transformed) in the model, where available, to account for differences between small and large plots and to illustrate the influence on the results. Based on the availability of environmental variables, 565,063 plots were included, with 100,378 of these plots containing at least one parasitic plant species.

The models were fitted with the *gbm.step* routine (Elith *et al*. 2008) in the ‘*dismo*’ package (Hijmans *et al*. 2023) in R v. 4.3.0 (2023‐04‐21; R Core Team 2023). We used Bernoulli distribution and applied weights for each parasitic plant functional type separately, based on the approach of Yu *et al*. (2020). Plot‐level weighting was set to 1 if there was no occurrence of a species belonging to the focal functional type of parasite; otherwise, the functional type's relative cover was used as weight. We scaled the weighted data as follows:

$$\text{Scaled weight}=\begin{array}{l} \frac{\text{weight}-\text{min}.\;\text{weight within the funct.}\;\text{type}}{\text{max}.\;\text{weight within the funct.}\;\text{type}-\text{min}.\;\text{weight within the funct.}\;\text{type}}\times \;100+1 \end{array}$$

The ratio of the training/test subset was set to 0.5, the learning rate was set to 0.001, and the tree complexity to 5. The optimal number of regression trees was identified through a 10‐fold cross‐validation procedure. From the resulting BRT models, we extracted the values of the relative importance of individual predictors. We used partial dependence plots to visualise the relationships between the predictor and the response variables.

We tested for spatial autocorrelation in the residuals of each model using Moran's *I* statistics for distance classes defined by Sturges's rule, using the *‘corrlog’* function in the *pgirmess* package (Giraudoux 2024). We detected no signal of spatial autocorrelation in the residuals of any of our models (see Appendix S8).

Because of intrinsic correlations among the EIVE gradients (Tichý *et al*. 2023), we opted for simple pairwise comparisons between plots with and without parasitic plant functional types rather than fitting multiple regression models. To test for significant differences in mean EIVE values, we applied a modified permutation test (Zelený & Schaffers 2012). In each permutation we randomly permuted the EIVE values of species, then calculated the mean randomised EIVE values for each plot, and computed the mean difference between plots with and without each functional type (Diff_rand_). This procedure was repeated 1,000 times to generate a null distribution of EIVE value differences (for histograms of the distribution see Appendix S9). We compared this null distribution to the real observed differences in mean EIVE values (Diff_real_). The Standardised Effect Size (SES) was obtained as: SES = (Diff_real_ – mean (Diff_rand_))/SD (Diff_rand_), and the *P* value was determined by the count of instances where Diff_rand_ exceeded Diff_real_ in the direction of the SES, divided by the number of permutations.

#### Ecological niches

For individual parasitic species with at least 50 occurrences in the dataset, we estimated their ecological niches by calculating their 95% range (minimum, mean, and maximum) along the gradients of environmental variables (climate and TRI), habitat descriptors and the mean EIVE values. We determined the density using kernel density estimates (*‘density’* function in the *stats* package) with Gaussian distribution and square‐rooted relative cover as weights (square‐rooted cover of a plot divided by the sum of square‐rooted covers of a species over all plots). Relative density (density value divided by the sum of all density values of a species for a variable) was then used to calculate the 0.025 and 0.975 quantiles. The mean was calculated as the arithmetic mean, weighted by the same weights used for the density calculation. Plots showing the niches for all species with at least 50 occurrences for all environmental variables are presented in Appendix S10.

To present the ecological niches of parasitic plant species in the context of their phylogenetic relationships, we obtained the phylogeny using the R package *V.PhyloMaker2* (Jin & Qian 2022). The mega phylogeny implemented in the package was derived from two mega‐trees (Zanne *et al*. 2014; Smith & Brown 2018). Data were only available for 37 parasitic species (~46% of all parasitic species). The data for the missing species were added using the ‘scenario 3’ approach implemented in the same R package (for further details, see Jin & Qian 2022).

## RESULTS

### Spatial patterns and habitat associations

We identified 244 parasitic plants out of a total of 11,126 plant taxa in our dataset (= 2.2%; see Appendix S4 for additional information on the species pool). The majority of parasitic plant species were euphytoid hemiparasites (176 species), followed by obligate root parasites (52), parasitic vines (12), and mistletoes (4). We excluded mistletoes from further analyses due to their limited representation in the dataset.

**Fig. 1:**
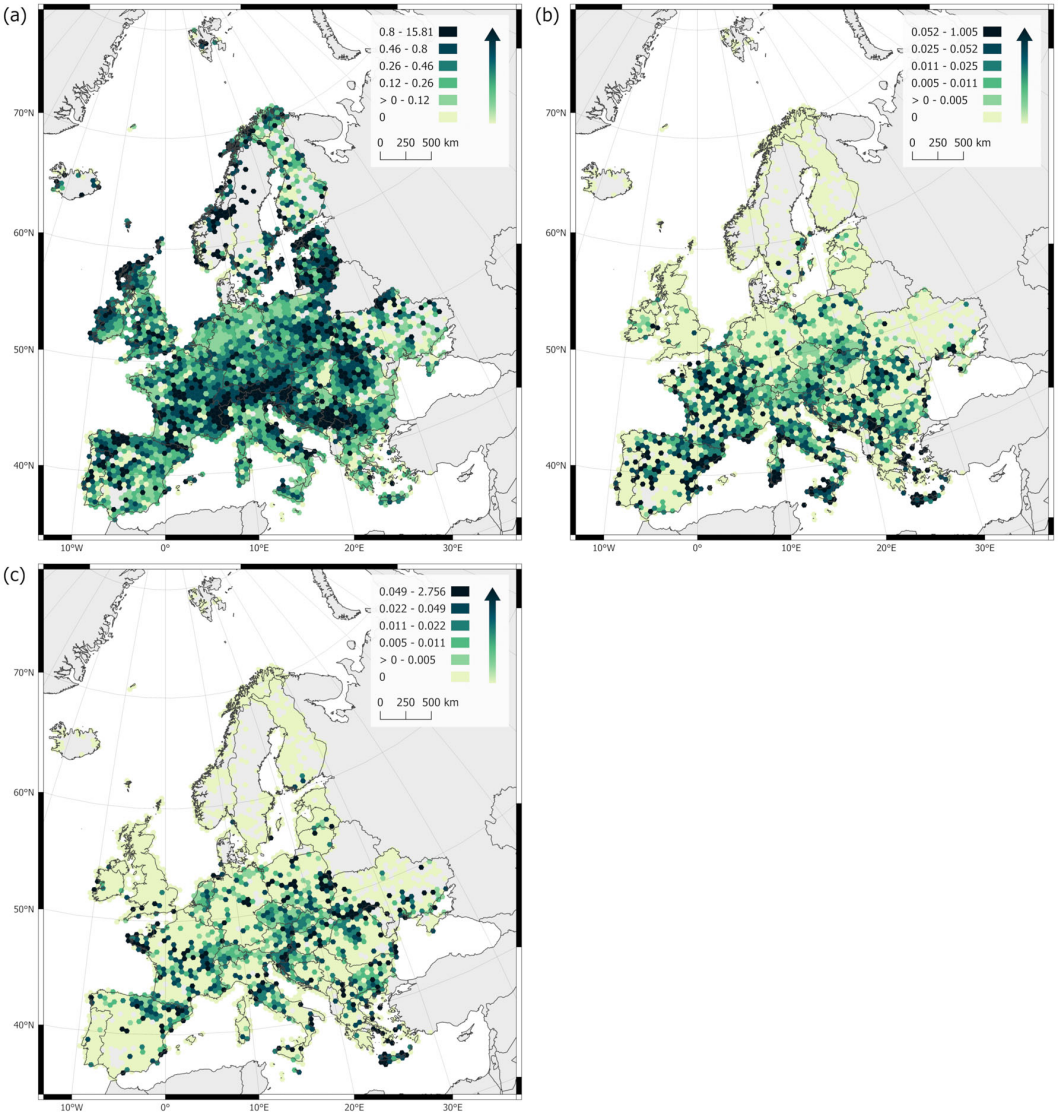
Mean relative cover (%) of (a) euphytoid hemiparasites, (b) obligate root parasites and (c) parasitic vines in Europe, per grid cell. Grid cells are 50 km in latitude. Values are only calculated for grid cells containing at least five plots. Projection: Lambert Azimuthal Equal Area (IAU:2015, EPSG:39980).

**Fig. 2:**
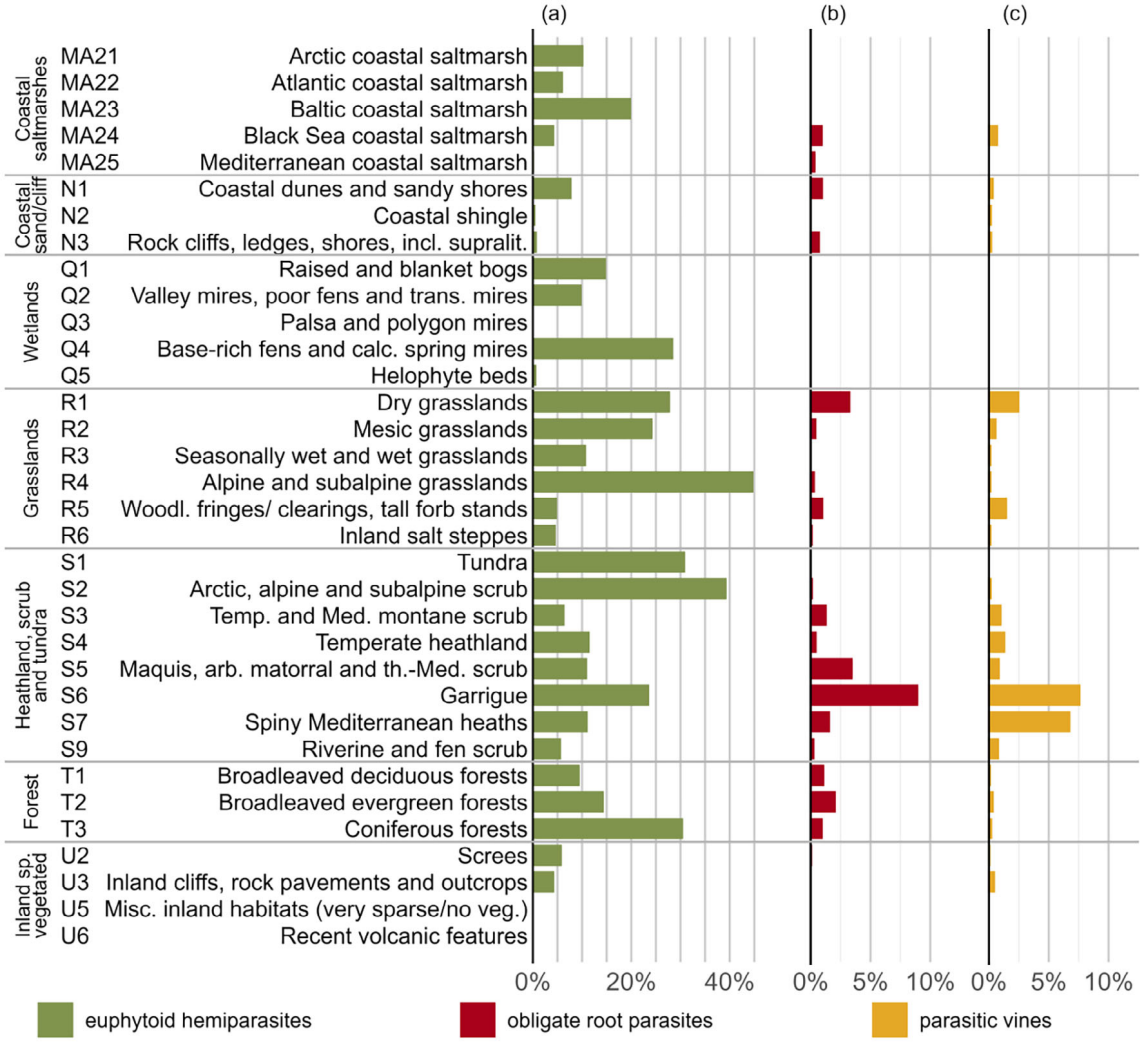
Occurrence of (a) euphytoid hemiparasites, (b) obligate root parasites, and (c) parasitic vines in European vegetation. For each medium‐level EUNIS habitat type, the percentage of plots with at least one record of the different functional types of parasitic plants is displayed.

The distribution maps show that parasitic plants occur throughout Europe, yet distinct regional patterns and differences between the functional types are apparent (Fig. 1). Euphytoid hemiparasites are most common in the European vegetation (Fig. 2) with the highest relative cover in mountainous and northern regions (Fig. 1). They are relatively less represented in the Mediterranean area and in the lowlands. The lowland eastern Baltic region is a notable exception as a hotspot for euphytoid hemiparasites. Obligate root parasites are most abundant in the Mediterranean and southern temperate zones. Parasitic vines, while also mostly present in the south, are less prevalent in the Mediterranean, apart from the eastern Mediterranean islands. Both groups are absent in Northern Europe, showing a distinct limit at the transition between the temperate and hemiboreal zones.

Parasitic plants are generally frequent in all types of European vegetation except coastal sands and cliffs and sparse inland vegetation (Fig. 2). This omnipresence, however, is mainly driven by euphytoid hemiparasites. They are most common in alpine and arctic habitats, where they were found in more than one‐third of the vegetation plots, followed by diverse habitats including coniferous forests, calcareous fens, mesic and dry grasslands, garrigues and coastal saltmarshes of Northern Europe. In contrast, obligate root parasites and parasitic vines are most frequent in dry grasslands and Mediterranean scrub habitats. Despite these large‐scale trends, habitat preferences of individual parasitic plant species may be quite distinct, as summarised in Table S2.

### Environmental drivers

**Fig. 3:**
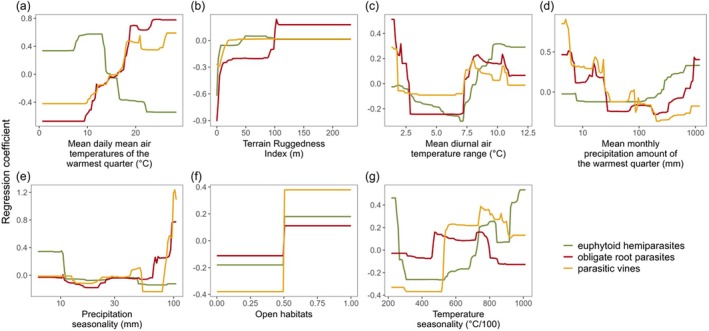
Partial dependence plots from the boosted regression tree model (BRT) showing the effect of the most important environmental variables (a–g) on the relative cover of parasitic plant functional types in Europe. Lines show the curves of the fitted functions. Values of precipitation (d, e) are shown on log‐scaled axes. See Appendix S8 for partial dependence plots of all environmental variables.

The boosted regression tree models (BRTs) of environmental predictors identified climate variables as the most important predictors shaping the niches of parasitic plants, followed by topography (terrain ruggedness) and broad habitat properties (open, wet, saline; Table 1). All three functional types avoid flat landscapes (low topographic ruggedness; Fig. 3). They also tend to prefer open habitats and avoid wet environments. The responses to the principal climate gradients are, however, very different. Euphytoid hemiparasites are associated with low summer temperatures, high summer precipitation, and low precipitation seasonality. Parasitic vines show contrary responses to these environmental predictors, as do obligate root parasites, except for their bimodal response to summer precipitation. All parasitic plants are common in conditions of high diurnal temperature variation. Obligate root parasites and parasitic vines display a second optimum at low values, which is less pronounced in euphytoid hemiparasites.

**Table 1:** Relative influence of predictors in the boosted regression tree (BRT) models on relative cover of the different parasitic functional types in Europe.

	euphytoid hemiparasites	obligate root parasites	parasitic vines
Mean diurnal air temperature range (°C)	16.0 (•)	8.3 (•)	8.4 (•)
Temperature seasonality (°C/100)	9.8 (•)	7.7 (•)	7.3 (•)
Mean daily mean air temperatures of the warmest quarter (°C)	26.7 (↘)	17.5 (↗)	11.4 (↗)
Annual precipitation amount (mm)	2.5 (↗)	6.5 (↗)	6.7 (↘)
Precipitation seasonality (mm)	1.9 (↘)	9.6 (↗)	11.4 (↗)
Mean monthly precipitation amount of the warmest quarter (mm)	8.5 (↗)	10.6 (•)	7.7 (↘)
Mean monthly potential evapotranspiration (mm)	2.2 (↘)	6.7 (•)	9.8 (•)
Terrain Ruggedness Index (m)	6.9 (↗)	15.6 (↗)	11.6 (↗)
Wet habitats	9.3 (↘)	3.8 (↘)	4.3 (↘)
Open habitats	11.6 (↗)	5.5 (↗)	8.4 (↗)
Saline habitats	0.3 (↘)	0.0 (↘)	0.6 (↘)
Plot size (m^2^)	4.2 (↗)	8.3 (↗)	12.5 (•)

Score values of each model are scaled, summing up to 100% (numbers displayed are rounded). Variables with influence <5% are shown in grey. Symbols indicate a more positive (arrow up), negative (arrow down) or complex (dot) correlation of the variables to the functional type.

**Fig. 4:**
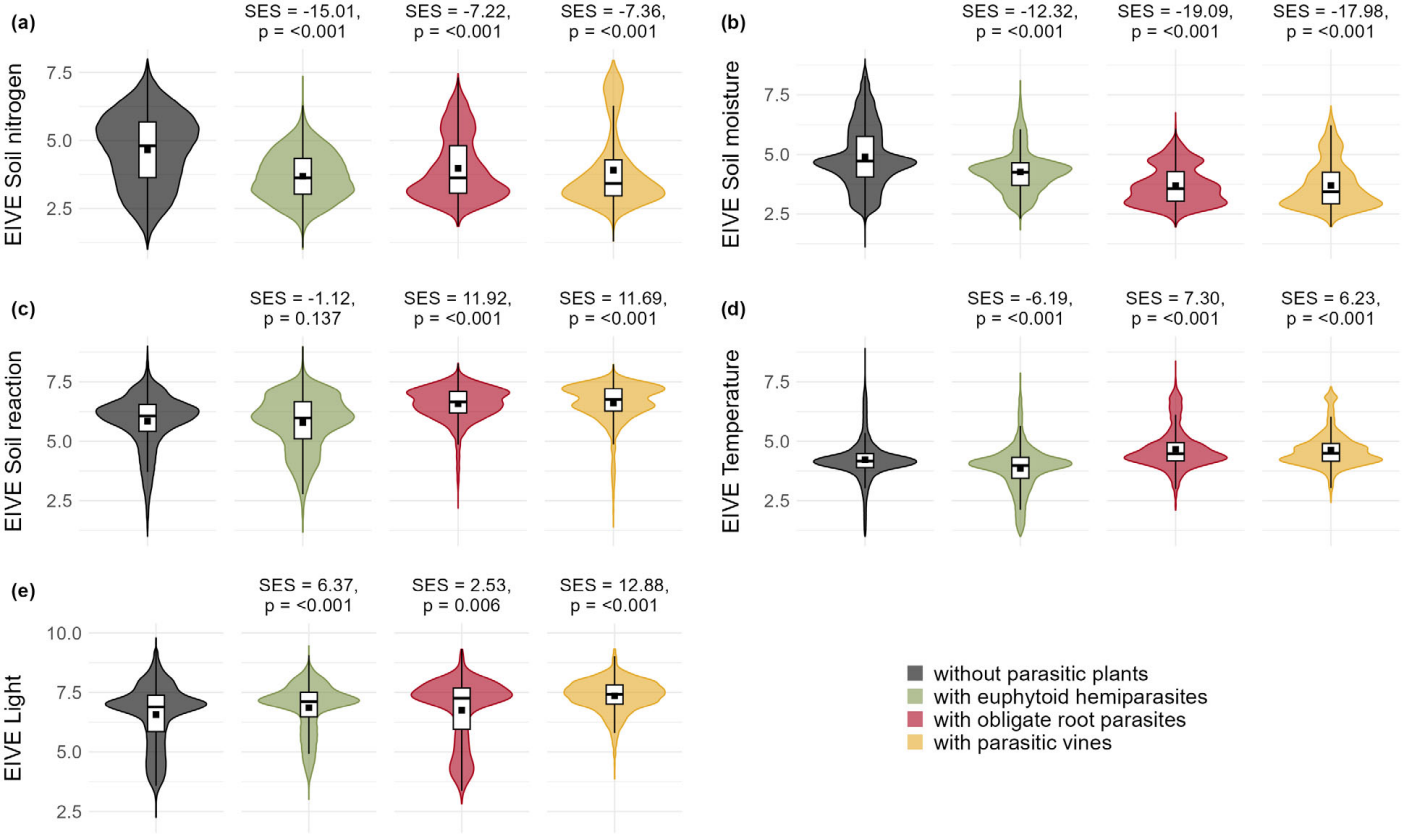
Violin plots showing the distribution of EIVE values in plots classified by occurrence of parasitic plant functional types for (a) Soil nitrogen, (b) soil moisture, (c) soil reaction, (d) temperature, and (e) light. Boxplots represent median, quartiles and non‐outlier ranges, while outliers are retained in the density computation for the violin plot construction. Black squares indicate the mean. SES indicates the Standardised Effect Size of the difference between the mean EIVE value of plots with and without a functional type of parasitic plant. *P* values indicate the statistical significance of these differences.

The comparisons revealed significant differences between plots with and without parasitic plants (Fig. 4). All functional types of parasitic plants were associated with conditions of significantly lower soil nitrogen (N) and soil moisture, as well as higher light availability than expected by chance. Euphytoid hemiparasites were notably absent from the top 25% of the soil N gradient, indicating a sharp limit of their ecological niche. In contrast, obligate root parasites and parasitic vines were more frequent in low soil N conditions, but occurred across the entire soil N gradient. Similar, yet distinct, limits are apparent in response to soil moisture and light, with both obligate root parasites and parasitic vines avoiding high soil moisture, while parasitic vines also avoid low light conditions. We found divergent trends in EIVE Temperature, as euphytoid hemiparasites were growing in significantly colder conditions, whereas the other two types were significantly more prevalent in warmer conditions than expected by chance. EIVE Soil reaction was significant only for obligate root parasites and parasitic vines, both of which were found in environments with higher soil reaction values than expected by chance.

### Ecological niches

**Fig. 5:**
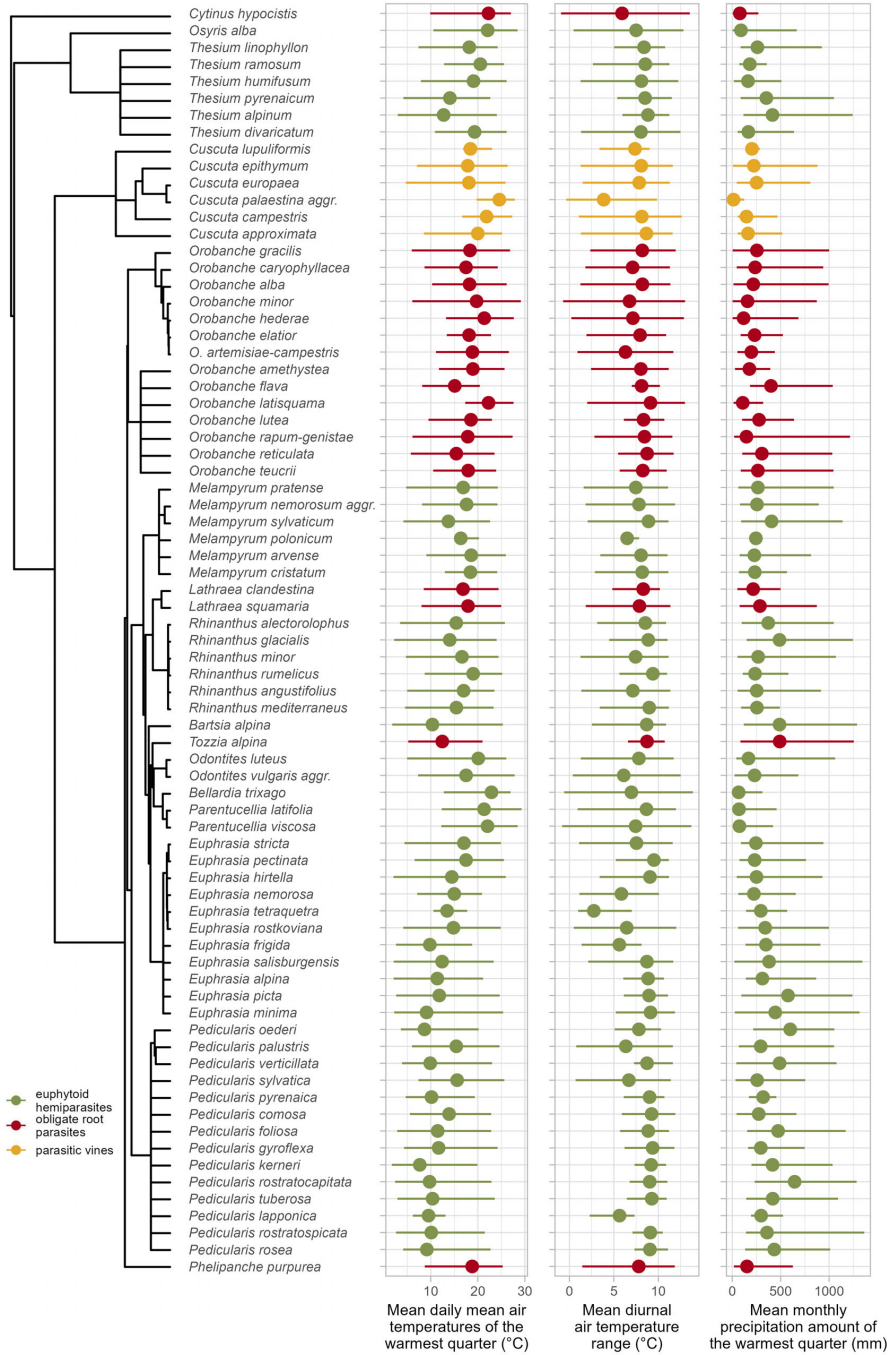
Ranges (lines) and optima (full points) of parasitic plant species along selected temperature and precipitation gradients in Europe. The ranges were defined as intervals covering 95% of cover‐weighted presences of given species, while the optima were defined as cover‐weighted averages. Phylogenetic relationships among species are displayed on the left‐hand side. For the figure, we retained all analysed species of parasitic vines and obligate root parasites with at least 50 occurrences, but selected the 50 most frequent euphytoid hemiparasites to preserve legibility.

The individual species optima along environmental gradients generally exhibit consistent preferences with the corresponding functional types, although we identified some species opposing these trends or showing broader ranges than suggested by the BRT analysis (Fig. 5). Most notable are *Osyris alba*, several *Thesium* species, *Odontites luteus*, *Bellardia trixago*, and *Parentucellia* species, which are euphytoid hemiparasites growing in high summer temperatures and conditions of low precipitation. In addition, most *Rhinanthus* and *Melampyrum* species either show wide ranges in relation to climate variables or grow at intermediate conditions. Individual species of obligate root parasites and parasitic vines follow the patterns for summer temperature, diurnal temperature range, and summer rainfall indicated by the BRT analysis for the groups. Some taxa, like *Cuscuta palaestina* aggr., and certain *Orobanche* species, exhibit either very wide or very narrow ranges, showing preferences for extremes of their functional type's typical range. *Orobanche flava* and *Tozzia alpina* grow in cold‐summer and high‐precipitation areas, and thus represent exceptions among obligate root parasites.

**Fig. 6:**
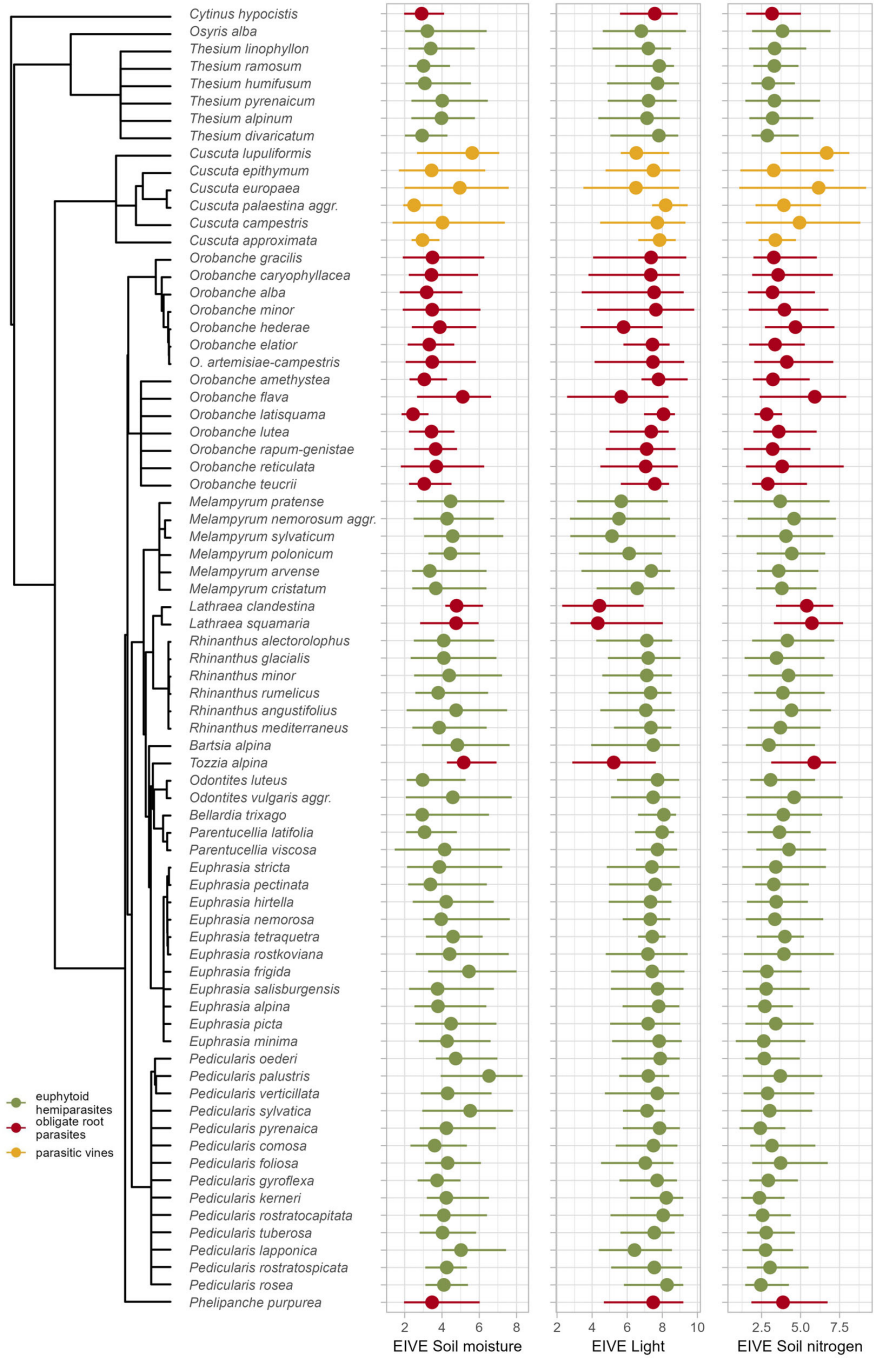
Ranges (lines) and optima (full points) of parasitic plant species along selected gradients of Ecological Indicator Values for Europe (EIVE). For each vegetation plot, we calculated means of EIVE values excluding the focal species from the calculation. Ranges were defined as intervals covering 95% of cover‐weighted presences of given species, while optima were defined as cover‐weighted average. Phylogenetic relationships among species are displayed on the left‐hand side. For the figure, we retained all analysed species of parasitic vines and obligate root parasites with at least 50 occurrences, but selected the 50 most frequent euphytoid hemiparasites to preserve legibility.

Species preferences along EIVE gradients display great variability within functional types (Fig. 6). All euphytoid hemiparasites occur in vegetation with low to moderate EIVE Soil nitrogen values, but there are notable differences between species. Some species occur in very low soil nitrogen conditions (many *Thesium*, *Euphrasia and Pedicularis* species, *Bartsia alpina*, and *Odontites luteus*), while others prefer moderate nitrogen levels (most *Melampyrum* and *Rhinanthus* species) or can tolerate a range of conditions (*Osyris alba*, *Melampyrum pratense, Rhinanthus alectorolophus*, and *Euphrasia rostkoviana*). *Melampyrum* species tolerating low EIVE Light values and *Pedicularis palustris*, associated with high EIVE Soil moisture values, represent additional divergences from the majority trends in euphytoid hemiparasites (Fig. 6). Among obligate root parasites, *Orobanche flava, Lathraea* species, and *Tozzia alpina* are distinct by occurring at high EIVE Soil moisture conditions, which is in contrast to the majority trend (Fig. 6). These species and *O. hederae* also occur at low EIVE Light values, while some other *Orobanche* species display a wide tolerance to light levels. This caused the relatively weak pattern of obligate root parasite occurrence in relation to EIVE Light (as reflected in the SES; Fig. 4). In contrast, parasitic vine species are uniformly associated with high EIVE Light but show rather divergent optima and wide ranges along the EIVE Soil nitrogen and partly also to EIVE Soil moisture gradients. Especially *Cuscuta lupuliformis and C. europea* tend to occur in moister and higher N conditions compared to other parasitic vine species, contributing to the secondary optima of this functional type along these gradients (Fig. 4).

## DISCUSSION

Our results indicate that parasitic plants represent a notable portion of the European species pool, accounting for approximately 2.2% of the total number of species aggregates in our dataset. This figure is slightly higher than the global proportion of parasites in angiosperms (~1.6%; Nickrent 2020) but is comparable to other temperate regions (e.g., China 2.3%, Zhang *et al*. 2018; Nepal 2.9%, Joshi *et al*. 2000; O'Neill & Rana 2016). Compared to the global representation, the European flora of angiosperm parasites contains proportionally more euphytoid hemiparasites and obligate root parasites but relatively few mistletoes (for exact numbers, see Appendix S4). These differences in species representation are likely driven by evolutionary history and biogeography rather than by ecological success alone. The most represented parasitic family, Orobanchaceae – comprising most European euphytoid hemiparasites and obligate root parasites – likely originated in former Laurasia, possibly in Eastern Asia (Wolfe *et al*. 2005), and underwent notable diversification in temperate Western Eurasia (Těšitel *et al*. 2010). The genus *Cuscuta*, representing parasitic vines, likely originated in Central or South Asia (García *et al*. 2014). In contrast, the European euphytoid hemiparasite and mistletoe species of Santalales belong to clades that have Gondwanan evolutionary origins (for *Osyris* and *Thesium* suggested by Der & Nickrent 2008; for *Loranthus* suggested by Liu *et al*. 2018; for *Viscum* suggested by Maul *et al*. 2019). Bottleneck effects associated with long‐distance dispersal might therefore explain their low diversity in the Palearctic.

Our results confirm that parasitic plants are a ubiquitous component of European natural and semi‐natural vegetation. However, this statement primarily applies to euphytoid hemiparasites, which are widely distributed across vegetation types and regions in Europe, as indicated by previous regional studies (Ter Borg 1985; Těšitel, Fibich, *et al*. 2015). In contrast, parasitic vines and obligate root parasites are much rarer and confined to specific habitat types and regions. The geographic distribution patterns and abundance analyses along environmental gradients have identified principal drivers of parasitic plant occurrence in Europe. While some of these drivers, e.g., mean temperature and precipitation seasonality, have been recognised in previous studies (Ter Borg 1985; Grenz & Sauerborn 2007; Jiang & Zhang 2021), others, e.g., habitat openness and topographic heterogeneity (TRI), represent novel findings, especially for the European continent. Among all the assessed drivers, only TRI (Fig. 3) has a consistent impact across all parasitic plant functional types. This suggests that parasitic plants avoid flat landscapes, but even minimal topographic heterogeneity supports their abundance. This low incidence of parasitic plants in flat lowlands is probably related to the high land‐use intensity in the lowlands of Northern Central Europe, and Northern and Eastern Europe (Rega *et al*. 2020; Dou *et al*. 2021), and the dependence of many parasitic plants on low‐intensity management practices, especially in grasslands (Svensson & Carlsson 2005; Blažek & Lepš 2015). In this respect, the Eastern Baltic region has an exceptional status; despite its flat terrain, it serves as a hotspot for euphytoid hemiparasites (Fig. 1). This region has a lower land‐use intensity than other European lowland areas (Rega *et al*. 2020; Dou *et al*. 2021) and was demonstrated to harbour high biodiversity in other studies (e.g. for forest vegetation: Večeřa *et al*. 2019; or plant life forms and diversity of hemicryptophytes: Midolo *et al*. 2024).

We identified distinct limits of distribution of obligate root parasites and parasitic vines in relation to climate, towards northern and colder regions. Their sharp boundaries suggest that they are likely driven by fundamental biological constraints specific to these parasitic plant functional types. Cold temperatures may inhibit the efficiency of their heterotrophic nutrient acquisition pathway, particularly the translocation of carbon from the host and its subsequent metabolism for energetic and structural purposes, due to the generally low respiratory rates in cold temperatures (Lambers *et al*. 2008). Similar latitudinal diversity patterns observed in ectothermic animals (amphibians and reptiles; Buckley *et al*. 2012; Sillero *et al*. 2014) support the hypothesis that constraints of universal metabolic mechanisms influence these geographic boundaries. Strikingly, the occurrence pattern of euphytoid hemiparasites shows an opposite trend, with remarkable frequency and abundance extending into northern and cold regions beyond the limits of obligate root parasites and parasitic vines. Unlike the other types, euphytoid hemiparasites are less reliant on carbon translocation from their hosts, as they are photosynthetically active. Furthermore, several studies have demonstrated that in cold climates low soil temperatures can markedly restrict root growth, thereby limiting aboveground growth (Vapaavuori *et al*. 1992; Körner 2015; Nagelmüller *et al*. 2017). Such conditions may relatively favour the hemiparasitic strategy based on drawing belowground resources from other plants and thus avoiding costly investment in extensive root systems. This ability could be a mechanism underlying range expansions and diversifications of euphytoid hemiparasites in cold climates, as documented for the widespread genera *Euphrasia*, *Pedicularis* and *Bartsia* in a worldwide context (Li 1948; Taylor & Rumsey 2003; Gussarova *et al*. 2008). At the same time, many European genera of euphytoid hemiparasites are known to require cold stratification for seed germination (Weber 1981; Ter Borg 1985). This requirement may have contributed to the observed pattern, given the intrinsic correlation between the mean temperature of the warmest and coldest quarters (Pearson *r* = 0.67). However, only 1.48% of the plots in the analysis had mean winter temperatures of more than 10°C, which is recognised as the upper limit for germination of some well‐studied *Rhinanthus* species (Ter Borg 2005). Some euphytoid hemiparasites, including, e.g., several *Pedicularis* species, *Melampyrum pratense*, and both *Parentucellia* species, however, can germinate even at 20°C (SER, INSR, RBGK, Society for Ecological Restoration, International Network for Seed Based Restoration, Royal Botanic Gardens Kew 2023; Ter Borg 1985; Weber 1981). Thus, high winter temperatures do not appear to impose a strict limit on the distribution of euphytoid hemiparasites. Almost all European parasitic plants avoid wet soil conditions (Fig. 4), suggesting that plant parasitism has low adaptive value in wetland ecosystems. However, some euphytoid hemiparasites, such as *Pedicularis palustris* and *Pedicularis sylvatica* (and occasionally certain *Euphrasia* species; Appendix S2), grow in waterlogged bogs and mires, indicating that oligotrophic wet conditions can still be favourable for (some) euphytoid hemiparasites. Contrasting niche patterns in relation to summer precipitation (Fig. 3; positive in euphytoid hemiparasites, negative in parasitic vines, and bimodal in obligate root parasites), low values of which generally indicate summer drought, may partly be attributed to an inherent negative correlation between summer temperature and precipitation, but also to the biological differences between the parasitic plant groups. Parasitic vines and obligate root parasites lack developed leaves and mostly display only minimal levels of transpiration (Ehleringer & Marshall 1995; Westwood 2013; Clayson *et al*. 2014). Their resource acquisition mostly relies on the translocation of carbon‐rich resources from the host phloem (Westwood 2013; Těšitel 2016). This results in outstanding levels of water‐use efficiency that are particularly beneficial in dry, low‐precipitation environments. Consistent with this, semi‐arid and even arid areas globally host a remarkable diversity of obligate root parasites (Hydnoraceae, Lennooidae, *Cynomorium*) and *Cuscuta* vines (Heide‐Jørgensen 2008). In contrast, euphytoid hemiparasites acquire resources from the host via the transpiration stream (Ehleringer & Marshall 1995; Westwood 2013; Těšitel 2016). Many species, particularly within the Orobanchaceae, display elevated transpiration rates to facilitate xylem sap uptake. This strategy is most efficient in moderately dry conditions, where host plants close stomata to conserve water, which redirects the xylem stream to the hemiparasite (Těšitel *et al*. 2011). However, such an exploitative strategy is associated with low water‐use efficiency (Ehleringer & Marshall 1995), limiting the occurrence of euphytoid hemiparasites in highly water‐deficient conditions. Some euphytoid species have thus adapted their life cycles to coincide with the moist periods in otherwise dry regions (winter–spring annuals *Bellardia trixago* and *Parentucellia* spp. in the Mediterranean; *Odontites luteus* exploiting late‐summer rainy seasons in the continental steppe). A more conservative hemiparasitic strategy, associated with a higher water‐use efficiency, has been observed in some Santalales (e.g., Tennakoon 1997; Luo & Guo 2010). This adaptation may underlie the occurrence of *Osyris alba* and some *Thesium* species in very dry conditions (Figs. 5 and 6). Among obligate root parasites, *Tozzia alpina* and *Lathraea* spp. acquire their nutrition exclusively from the host xylem, similar to their euphytoid relatives, while excreting excess water through hydathode trichomes (Ziegler 1955; Světlíková *et al*. 2015). This clearly underpins their association with moister conditions compared to most obligate root parasites, resulting in the bimodal pattern of obligate root parasites' occurrence in relation to summer precipitation.

Soil nitrogen availability is particularly important for euphytoid hemiparasites, aligning with the N‐parasitism hypothesis that mineral nutrients are the crucial resources acquired by hemiparasites (Ehleringer & Marshall 1995). High nutrient availability in eutrophic conditions should reduce the advantage of hemiparasites and eventually lead to their competitive exclusion from communities of high productivity (Matthies 1995; Mudrák *et al*. 2014; Těšitel, Těšitelová, *et al*. 2015). Negative effects of high nutrient availability and community productivity on hemiparasite survival have been demonstrated in a number of field experimental studies (Van Hulst *et al*. 1987; Mudrák & Lepš 2010; Těšitel *et al*. 2013; Těšitel, Těšitelová, *et al*. 2015). However, only a few euphytoid hemiparasites have their optima in highly oligotrophic conditions (EIVE Soil nitrogen around 2.5). Most of them favour environments with moderately low to moderate nutrient availability. This aligns with experimental evidence showing that hemiparasites benefit from nutrients in a similar way to autotrophic plants, until competition from the host community becomes limiting above a certain threshold of productivity (Van Hulst *et al*. 1987; Těšitel *et al*. 2013; Těšitel, Těšitelová, *et al*. 2015). In contrast, soil N availability appears to be of relatively lower importance for obligate root parasites and parasitic vines, as indicated by their weaker negative correlation with EIVE N and broader range for this gradient. Some species of both of these types grow in high nitrogen conditions (*Tozzia alpina*, *Lathrea* species, *Orobanche flava*, *Cuscuta lupuliformes*, *C. europaea*). Nevertheless, obligate root parasites and parasitic vines may still hold an advantage in low‐nitrogen conditions, due to their lower metabolic nitrogen requirements (compared to photosynthetic plants) and their lack of sensitivity to competition in eutrophic environments.

Euphytoid hemiparasites, although varying in levels of carbon heterotrophy (Těšitel *et al*. 2010; Giesemann & Gebauer 2022), rely primarily on their own photosynthesis for organic carbon acquisition. Consequently, they would be expected to be positively associated with light availability. However, light availability was not identified as a particularly strong driver for the occurrence of the entire group of euphytoid hemiparasites. At least partially, this is caused by species of the genus *Melampyrum*, several of which grow in the forest understory, and can achieve high population densities even under relatively low‐light conditions, where their photosynthesis cannot provide sufficient carbon for their growth and reproduction (Světlíková *et al*. 2018). The biological mechanisms behind this are not clear, especially given the relatively low heterotrophic carbon estimates provided by stable isotope analyses (Giesemann & Gebauer 2022). Surprisingly, light availability seems more important for parasitic vines, despite their only rudimentary photosynthesis and ability to acquire carbon from the host phloem (Van Der Kooij *et al*. 2000). However, photosynthesis has been demonstrated to be crucial for *Cuscuta* seedling establishment, the first connection with the host, and seed production (Martinčová *et al*. 2019). Obligate root parasites display a significantly positive, although weak, association with light availability. The majority of fully heterotrophic species of this functional type, including most *Orobanche* and *Phelipanche* species, prefer high light conditions (Fig. 6). In contrast, *O. flava* and *O. hederae* grow in low‐light conditions, and are host‐specialised on tall herbs with a closed canopy and a shaded forest understory liana, respectively. Even *Tozzia alpina*, which is special in this group because of its photosynthetic shoots, grows preferably in shady conditions (Fig. 6). Thus, the apparent association of obligate root parasites with high‐light conditions may be a secondary effect of host preference rather than a direct requirement for light. Beyond community openness, specific traits of the host vegetation – such as mean life cycle length or plant height – may influence parasite–host interactions (Karlsson 1974; Ter Borg 1985; Těšitel, Fibich, *et al*. 2015). Incorporating functional trait data in future studies could provide deeper insights into the specific assemblage requirements of host communities supporting parasitic plants.

Fundamental biological properties and resource acquisition strategies drive the spatial distribution of parasitic plants, their occurrence in habitat types, and their ecological niches in Europe. Euphytoid hemiparasites, the most species‐rich and ecologically versatile group, thrive in diverse ecosystems by efficiently exploiting belowground resources, especially so in resource‐limited environments. In contrast, parasitic vines and obligate root parasites are restricted to narrower habitat ranges, suggesting that functional specialisation leads to high ecological specialisation. Euphytoid hemiparasites are linked to colder climates and habitats with lower productivity, where the limited availability of resources or harsh environmental conditions limit productivity of most plant species. In contrast, obligate root parasites and parasitic vines are associated with higher temperatures and drier soil conditions. These behave as typical heterotrophs and are less dependent on water availability, which is crucial for efficient photosynthesis. Due to their association with relatively cold climates and regions of low productivity, euphytoid hemiparasites are highly susceptible to climate warming and ongoing eutrophication, which has already been documented empirically (Jandt *et al*. 2022; Klinkovská *et al*. 2024). We might be losing species that have been recognised for their important ecological roles in natural ecosystems. In contrast, we could expect expansion of some obligate root parasites and parasitic vines – which may also include harmful agricultural weeds – in the near future.

## AUTHOR CONTRIBUTIONS

JT and IA conceived the idea. NF, IA, J‐CS, JPC and JT developed the analyses for the study. KK, IA, JT and NF prepared data for the study. NF, JPC and JT performed the analyses. J‐CS, IB, SB, JAC, AČ, JD, EG and TH contributed vegetation‐plot data. NF and JT wrote the manuscript. All authors contributed comments on the manuscript drafts and approved the final version.

## FUNDING INFORMATION

NF was supported by the Operational Programme Research, Development and Education—“Project Internal Grant Agency of Masaryk University” (No. CZ.02.2.69/0.0/0.0/19_073/0016943). NF, IA and JT were supported by the Czech Science Foundation (project number 24‐12161S). JCS was supported by the Center for Ecological Dynamics in a Novel Biosphere (ECONOVO), funded by the Danish National Research Foundation (grant DNRF173). IB and JAC were supported by a Basque Government Research Project (IT1487‐22).

## Supporting information


**Appendix S1.** Overview of the datasets included in this study.

**Appendix S2.** Additional information on the occurrence of parasitic plant species in EUNIS habitat types and the most common habitat types for all parasitic plant species.

**Appendix S3.** Additional information on the data structure, EUNIS habitat types and environmental variables.

**Appendix S4.** Additional information for functional types of parasitic plants and their occurrences in the species pool.

**Appendix S5.** Results of the RDA forward selection.

**Appendix S6.** Maps for the mean relative cover of parasitic functional types in EUNIS habitat types.

**Appendix S7.** Maps for the environmental variables used in the study.

**Appendix S8.** Details on fitting and evaluation of the boosted regression tree analysis.

**Appendix S9.** Additional information for the modified permutation test for EIVE values.

**Appendix S10.** Additional results for analysis of niches along all environmental variables.

## Data Availability

Openly available data include climate (https://chelsa‐climate.org), Terrain Ruggedness (https://doi.org/10.5069/G91R6NPX), and EIVE values (https://vcs.pensoft.net/article/98324/). The vegetation data that support this study's findings are not publicly available because of database restrictions. However, they can be requested directly from the EVA database (https://euroveg.org/eva‐database/obtaining‐data) by referring to the project reference number 137.
