# Supplementary material for: Parasitic plants in Europe: ecological niches and spatial patterns
Source: Plant Biol (Stuttg). 2025 Sep 18;27(7):1285–99. doi: 10.1111/plb.70099 (PMC12631522; doi:10.1111/plb.70099)
Supplement: Supplementary file 1 — Appendix S1. Overview of the datasets included in this study. [file PLB-27-1285-s009.pdf]

APPENDIX S1. Overview of the datasets included in the study

Table S1.1. The contribution of vegetation plots included in this study by database. For each database, we provide the total number of plots and the proportion of plots in the final dataset, after selection (N = 819,452). See the list of databases at https://euroveg.org/eva-database/databases for more details.

Database	Database custodian	Number of plots	Percentage of dataset (%)
**AMS-VegBank**	Alessandro Chiarucci	8596	1.049
**Albanian Vegetation Database**	Michele De Sanctis	435	0.053
**Ammophiletea Database**	Corrado Marcenò	2540	0.310
**Austria_VINCA**	Wolfgang Willner	33980	4.147
**Balkan Dry Grassland Database**	Kiril Vassilev	6583	0.803
**Balkan Vegetation Database**	Kiril Vassilev	11615	1.417
**Basque Country Database**	Idoia Biurrun	13940	1.701
**Beech Forest DB SE Balkan**	Aleksander Marinšek	1261	0.154
**Belgium-INBOVEG**	Els De Bie	11875	1.449
**Belgium-forest**	Els De Bie	3611	0.441
**Britain_nvcd**	John S. Rodwell	24981	3.049
**Bulgarian Vegetation Database**	Iva Apostolova	1469	0.179
**CBNA**	Sylvain Abdulhak	2643	0.323
**CircumMed Pine Forest database**	Gianmaria Bonari	3468	0.423
**Croatia_mix**	Zvjezdana Stančić	2515	0.307
**Croatian Vegetation Database**	Željko Škvorc	9711	1.185
**Czechia_nvd**	Milan Chytrý	67131	8.192
**Eastern European Steppe Database**	Denys Vynokurov	3277	0.400
**European Boreal Forest Database 1**	Anni Kanerva Jašková	39	0.005
**European Boreal Forest Database 2**	Anni Kanerva Jašková	6008	0.733
**European Coastal Vegetation Database-A**	John Janssen	2156	0.263
**European Mire VDB**	Tomáš Peterka	8110	0.990
**European Weed Vegetation Database**	Filip Küzmič	935	0.114
**France_SOPHY**	Emmanuel Garbolino	106548	13.002
**Germany Coastal VDB**	Maike Isermann	8	0.001
**Germany Vegetweb 1**	Florian Jansen	7353	0.897
**Germany Vegetweb 2**	Friedemann Goral	5865	0.716
**Germany Vegetweb 3**	Friedemann Goral	1416	0.173
**Germany_gvrd**	Ute Jandt	33985	4.147
**Germany_vegmv**	Florian Jansen	25950	3.167
**GrassVeg.DE**	Ricarda Pätsch	6272	0.765
**Gravel Bar Database_Europe**	Veronika Kalníková	768	0.094
**Greece_forests**	Ioannis Tsiripidis	623	0.076
**Greece_nat**	Panayotis Dimopoulos	4083	0.498

Table S1.1 continued

**High Mediterranean Mountains Database**	Gianpietro Giusso del Galdo	619	0.076
**Hungary**	János Csiky	3328	0.406
**Ireland_nvd**	Úna FitzPatrick	18415	2.247
**Italy_HabItAlp**	Laura Casella	3532	0.431
**Italy_UniRoma**	Emiliano Agrillo	21702	2.648
**Italy_mires**	Laura Casella	566	0.069
**Kriti**	Erwin Bergmeier	2789	0.340
**Latvian Grassland VDB**	Solvita Rūsiņa	4484	0.547
**Lithuania**	Valerius Rašomavičius	5490	0.670
**Macedonia**	Renata Ćušterevska	189	0.023
**Masaryk University Database 1**	Milan Chytrý	423	0.052
**Masaryk University Database 2**	Milan Chytrý	100	0.012
**Netherlands**	Stephan Hennekens	85845	10.476
**Netherlands Military sites**	Iris de Ronde	8092	0.987
**Nordic Vegetation Database 1**	Jonathan Lenoir	4714	0.575
**Nordic Vegetation Database 2**	Jonathan Lenoir	510	0.062
**Nordic_Baltic EDGG**	Jürgen Dengler	3148	0.384
**Poland**	Zygmunt Kącki	53999	6.590
**Poland Forest Database**	Remigiusz Pielech	3199	0.390
**RanVegDunes**	Alicia Acosta	1055	0.129
**Romania Grassland Database**	Eszter Ruprecht	18085	2.207
**Romania_indreica**	Adrian Indreica	6466	0.789
**SE Europe Forest DB**	Andraž Čarni	2307	0.282
**Schleswig-Holstein Db**	Joachim Schrautzer	2023	0.247
**Scottish Coastal Survey**	Robin Pakeman	5231	0.638
**Scottish Vegetation Survey**	Ruth Mitchell	5315	0.649
**Serbia_grasslands**	Svetlana Aćić	4641	0.566
**Serbian VDB**	Mirjana Krstivojević Ćuk	707	0.086
**Serra da Estrella database**	Jan Jansen	821	0.100
**Slovakia_nvd**	Milan Valachovič	29393	3.587
**Slovenia**	Urban Šilc	13152	1.605
**Spain_sivim**	Xavier Font	1573	0.192
**Spain_sivim_Catalonia**	Xavier Font	2246	0.274
**Spain_sivim_alpine**	Borja Jiménez-Alfaro	4278	0.522
**Spain_sivim_floodplains**	Idoia Biurrun	3253	0.397
**Spain_sivim_forests**	Juan Antonio Campos	4048	0.494
**Spain_sivim_grasslands**	Maria Pilar Rodríguez-Rojo	9024	1.101
**Spain_sivim_sclerophyllous**	Federico Fernández-González	2631	0.321
**Spain_sivim_sclerophyllous_pinus**	Federico Fernández-González	5630	0.687
**Spain_sivim_scrubs**	Rosario G Gavilán	2403	0.293

Table S1.1 continued

**Spain_sivim_wetlands**	Aaron Pérez-Haase	3740	0.456
**Switzerland Grassland DB**	Ariel Bergamini	7790	0.951
**Switzerland_forests**	Thomas Wohlgemuth	12662	1.545
**Turkey Forest Database**	Ali Kavgacı	44	0.005
**UK Floodplain Meadows Database**	Irina Tatarenko	10950	1.336
**Ukraine Grassland Database A**	Anna Kuzemko	6625	0.808
**Ukraine Halophytic Coastal VDB**	Tetiana Dziuba	500	0.061
**Ukraine Psammophytic VDB**	Tetiana Dziuba	622	0.076
**Ukraine_onyshchenko**	Viktor Onyshchenko	1987	0.242
**VegFrance**	Jan-Bernard Bouzillé	2529	0.309
**VegItaly**	Roberto Venanzoni	5735	0.700
**Viola db**	Angela Stanisci	1086	0.133
**WetVegEurope Database**	Flavia Landucci	6	0.001

Table S1.2. Contribution of vegetation plots included in this study by country. For each country, we provide the total number of plots in the original dataset (N = 1,081,084), and the total number and the proportion of plots in the final dataset after selection (N = 819,452).

Country	Plot number in the original dataset	Plot number in the final dataset	Percentage of the final dataset (%)
**Albania**	481	452	0.055
**Andorra**	564	440	0.054
**Austria**	40359	32915	4.017
**Belarus**	2081	0	0.000
**Belgium**	27775	20017	2.443
**Bosnia-Herzegovina**	3683	2796	0.341
**Bulgaria**	18634	15577	1.901
**Croatia**	16428	12494	1.525
**Czech Republic**	86554	66319	8.093
**Denmark**	286	238	0.029
**Estonia**	2852	2469	0.301
**Faroe Islands**	16	16	0.002
**Finland**	6220	4844	0.591
**France**	127074	105407	12.863
**Georgia**	316	0	0.000
**Germany**	118974	82995	10.128
**Greece**	13188	10130	1.236
**Hungary**	5622	3683	0.449
**Iceland**	499	431	0.053
**Ireland**	22852	18923	2.309
**Italy**	63951	47095	5.747
**Kosovo**	1052	642	0.078
**Latvia**	11053	8524	1.040
**Lithuania**	6899	5601	0.684
**Luxembourg**	216	207	0.025
**Macedonia**	2168	823	0.100
**Malta**	790	419	0.051
**Moldova**	209	182	0.022
**Monaco**	42	22	0.003
**Montenegro**	393	331	0.040
**Netherlands**	125806	93477	11.407
**Norway**	6763	4830	0.589
**Poland**	75644	59097	7.212
**Portugal**	3276	2688	0.328
**Romania**	30421	25058	3.058
**Russian Federation**	38305	0	0.000
**San Marino**	128	61	0.007
**Serbia**	11251	7179	0.876
**Slovak Republic**	35383	29608	3.613

Table S1.2 continued

**Slovenia**	20033	14826	1.809
**Spain**	70907	48505	5.919
**Svalbard and Jan Mayen Is**	230	183	0.022
**Sweden**	5041	3823	0.467
**Switzerland**	29317	26167	3.193
**Turkey**	611	0	0.000
**Ukraine**	15600	13155	1.605
**United Kingdom**	72334	46803	5.711

Figures S1.1 to S1.7 give an overview of the number of plots in the full dataset (dataset A, S3) by region for each broad-scale EUNIS habitat type used in the analysis (see Appendix S3 for more information on the dataset).

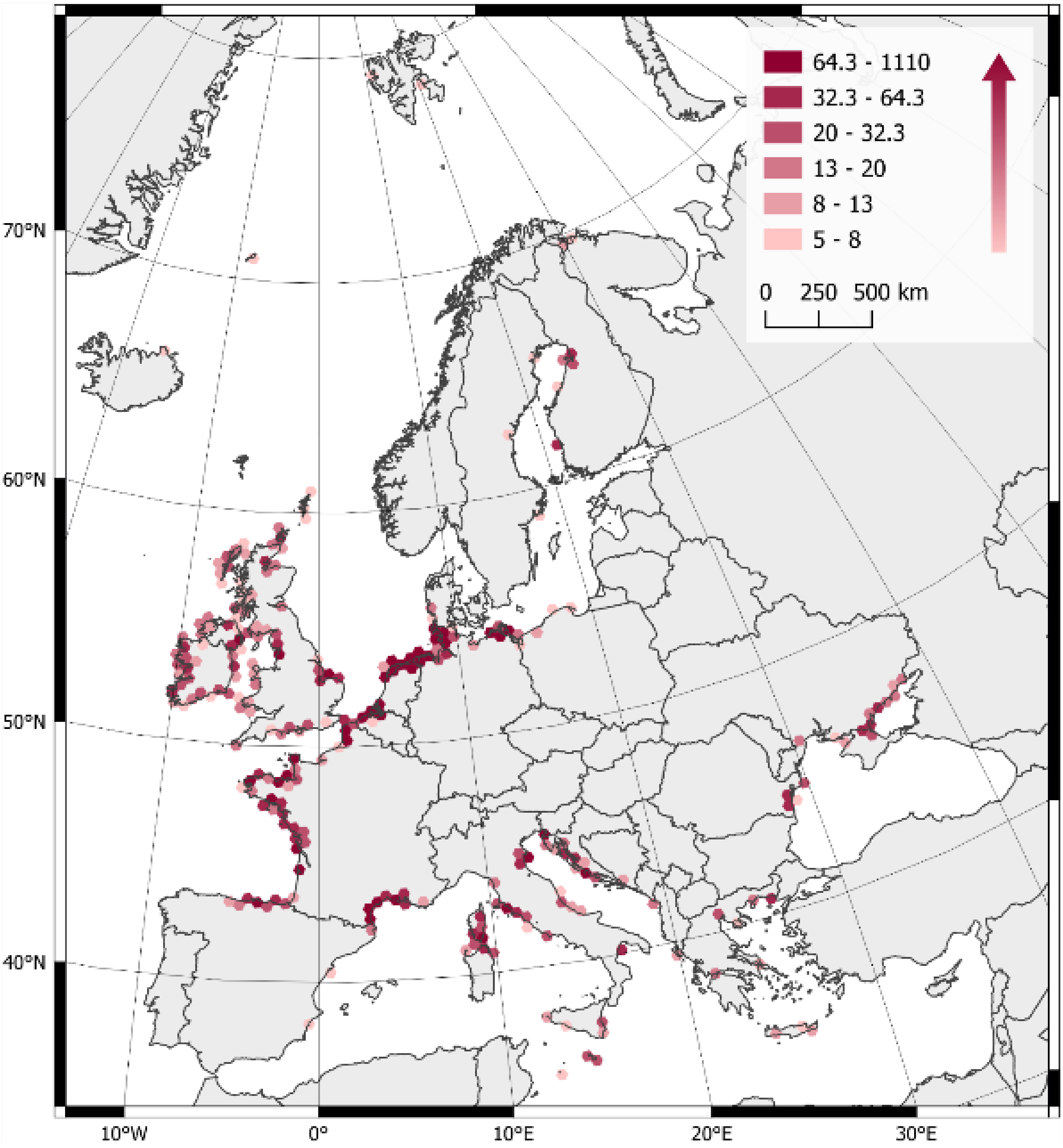

Fig. S1.1. Number of vegetation plots in the final data set for EUNIS habitat type M Coastal Saltmarshes. For the definition of the grid see Methods of the manuscript.

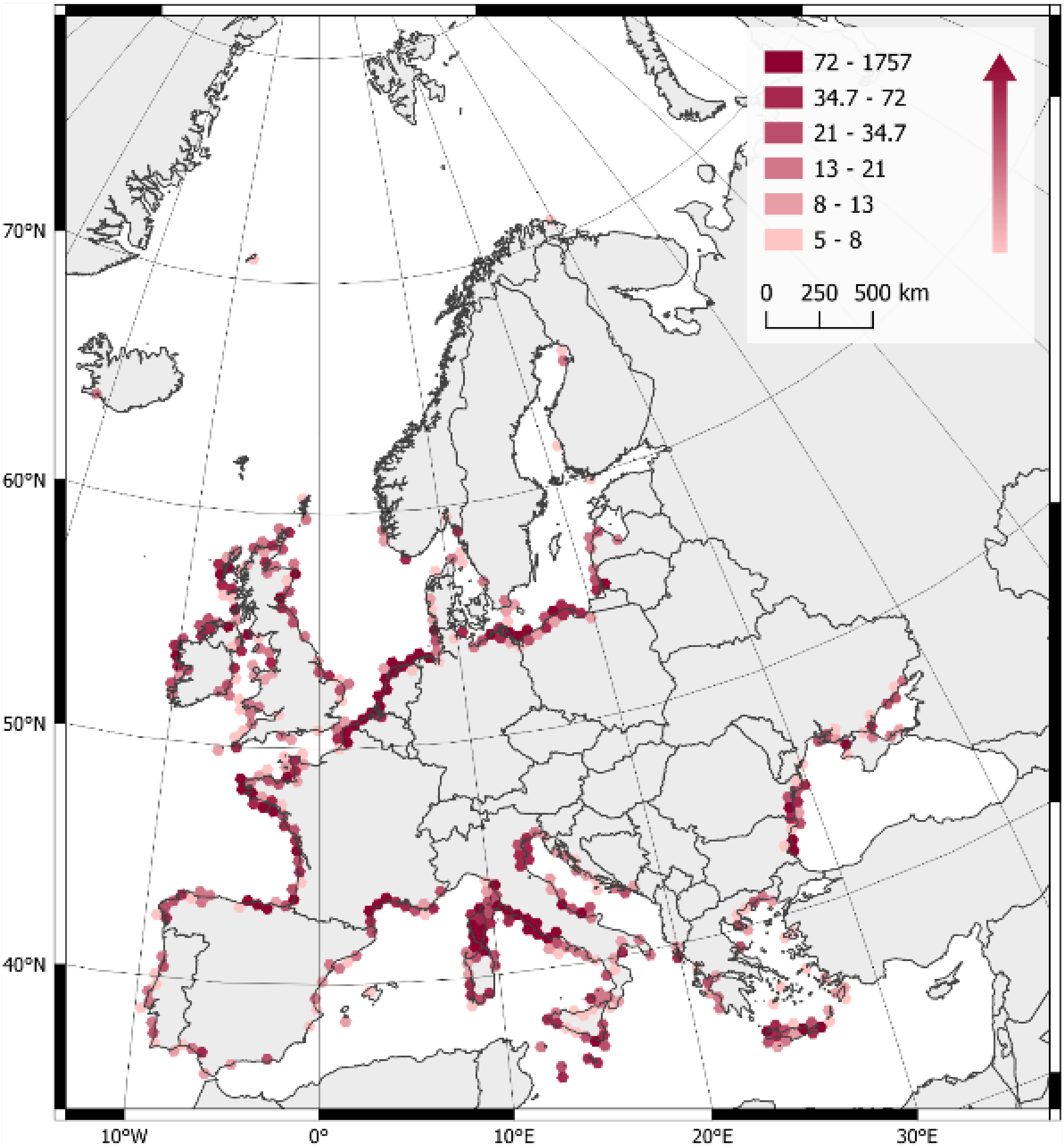

Fig. S1.2. Number of vegetation plots in the final data set for EUNIS habitat type N Coastal Sand and Cliff Habitats. For the definition of the grid see Methods of the manuscript.

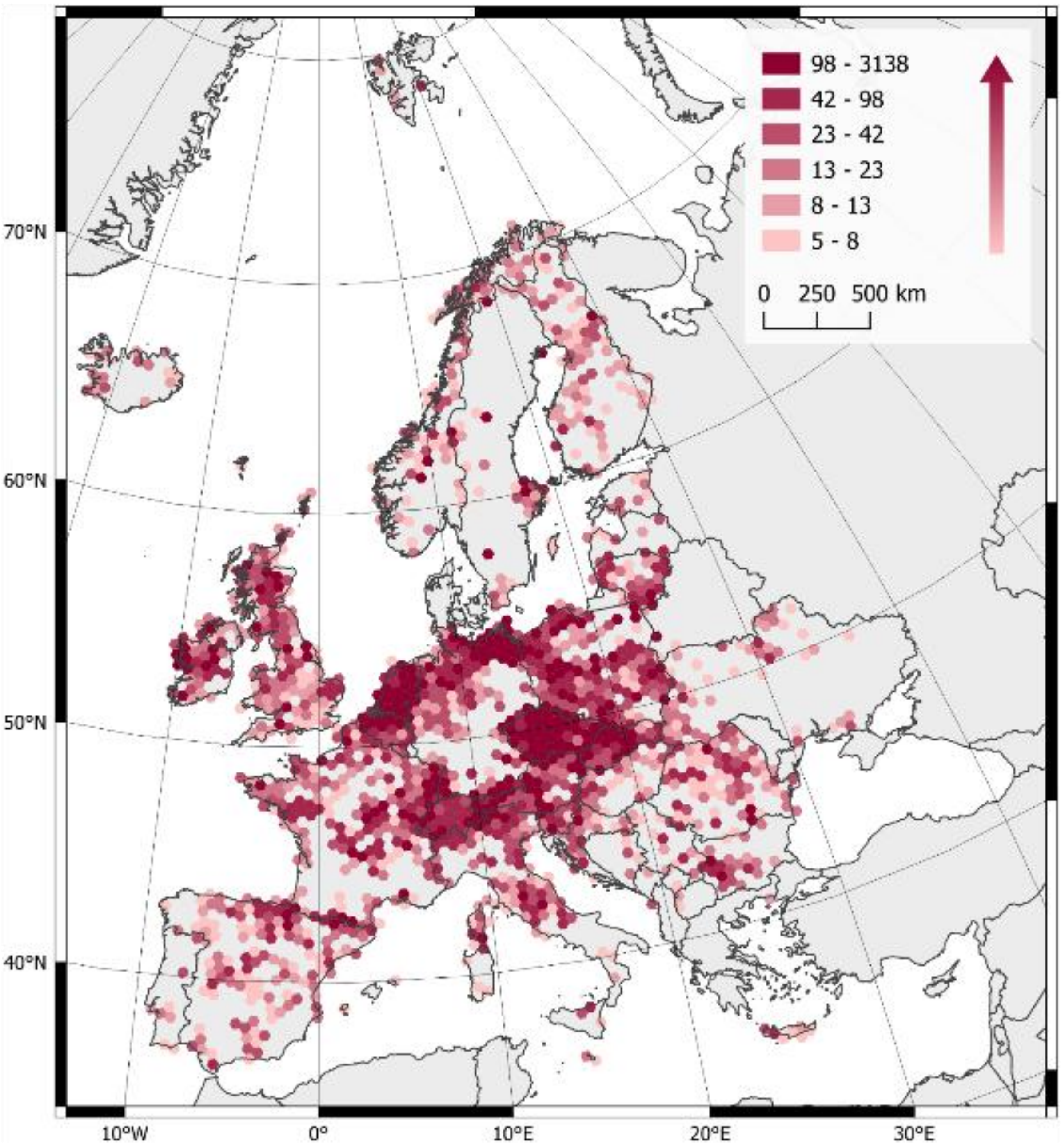

Fig. S1.3. Number of vegetation plots in the final data set for EUNIS habitat type Q Wetlands. For the definition of the grid see Methods of the manuscript.

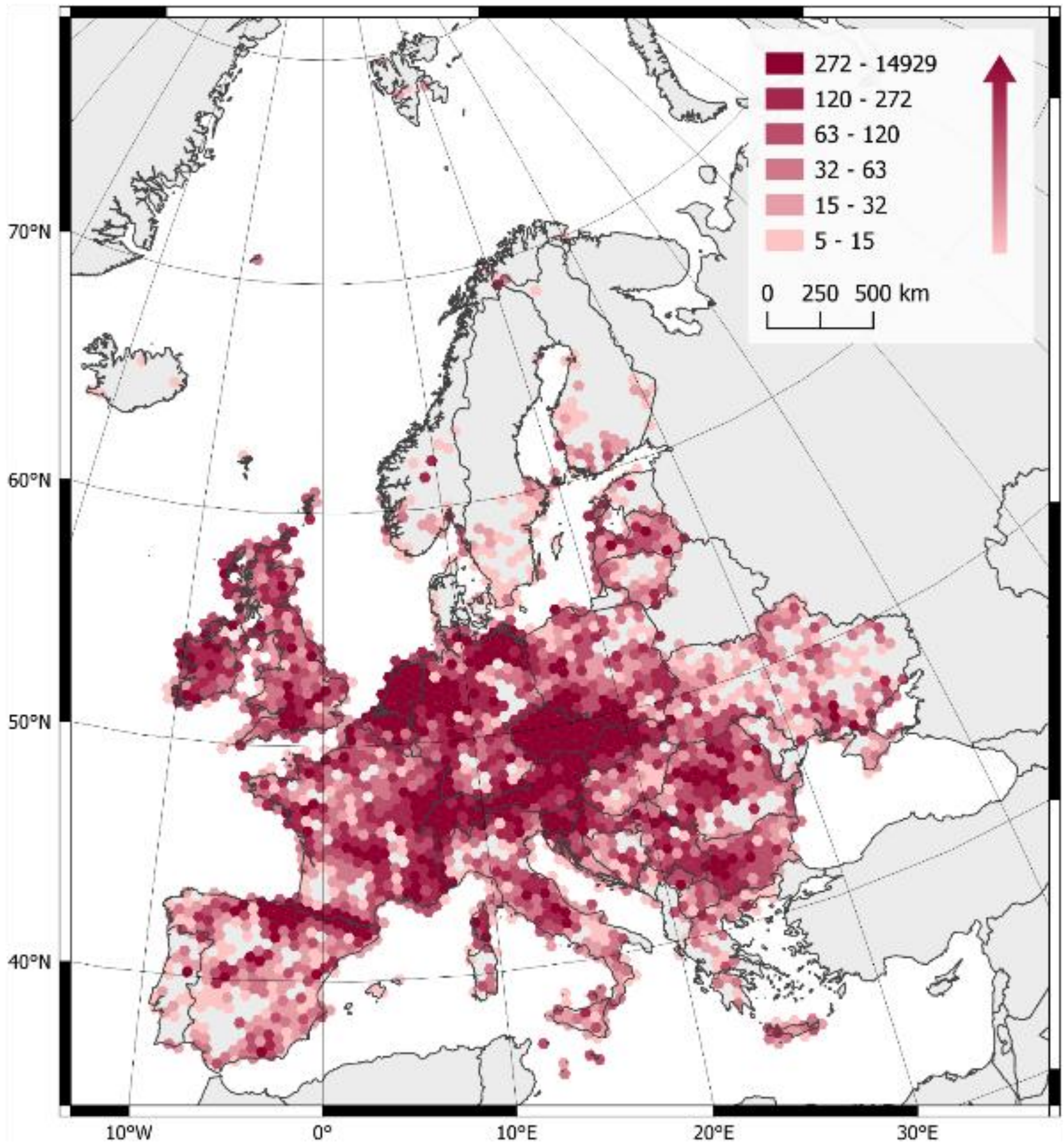

Fig. S1.4. Number of vegetation plots in the final data set for EUNIS habitat type R Grasslands. For the definition of the grid see Methods of the manuscript.

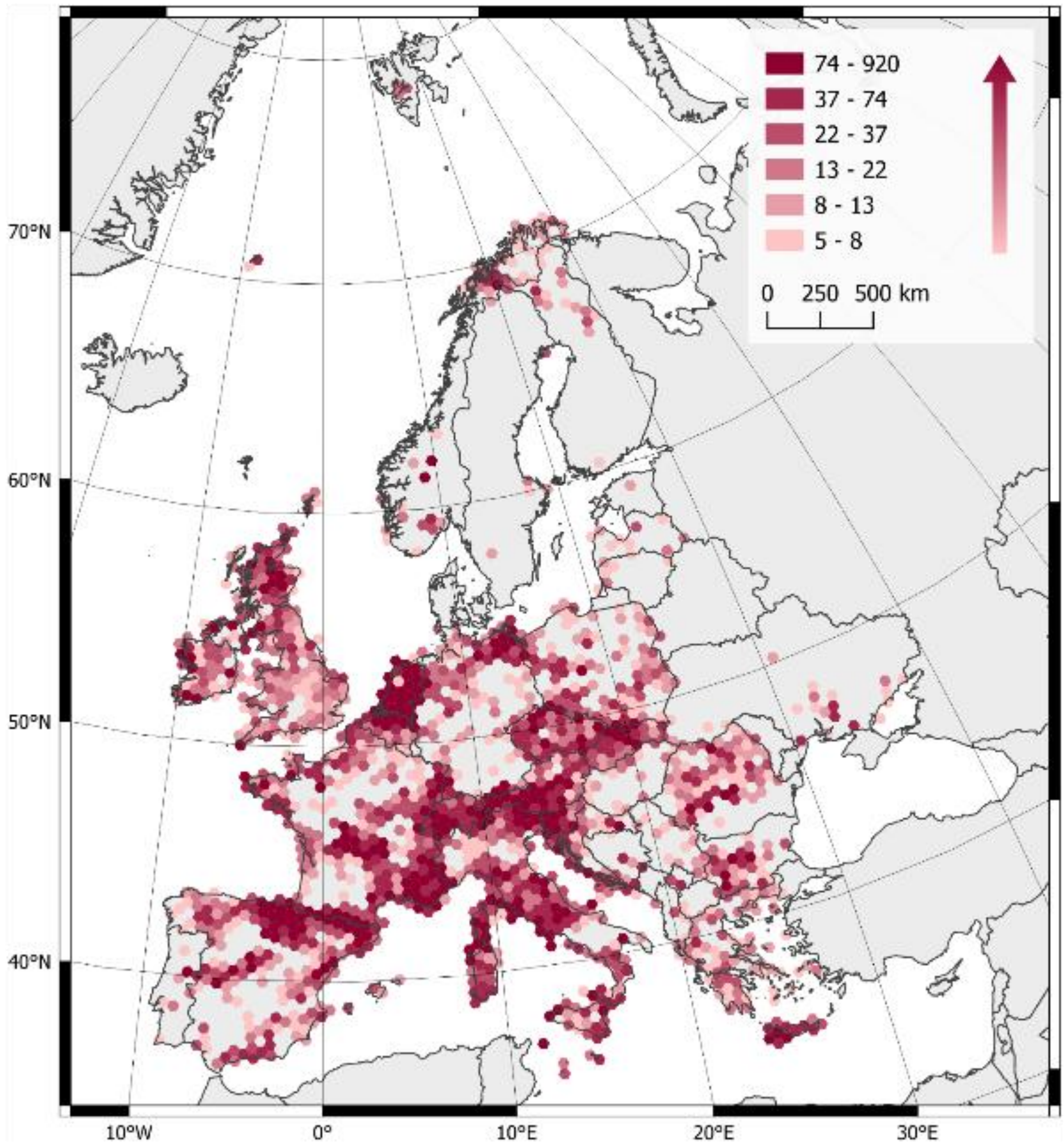

Fig. S1.5. Number of vegetation plots in the final data set for EUNIS habitat type S Heathlands, Scrub and Tundra. For the definition of the grid see Methods of the manuscript.

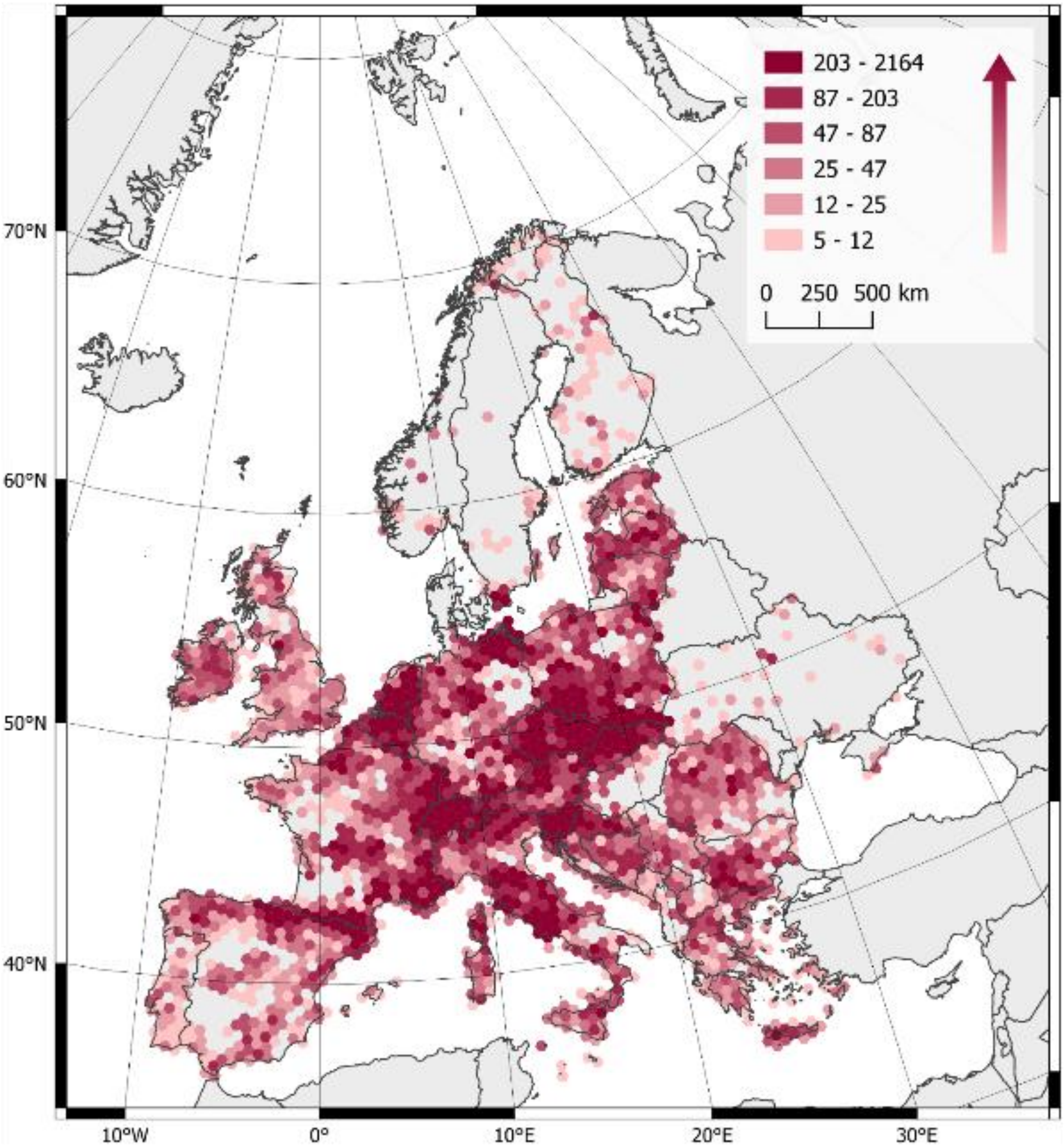

Fig. S1.6. Number of vegetation plots in the final data set for EUNIS habitat type T Forests. For the definition of the grid see Methods of the manuscript.

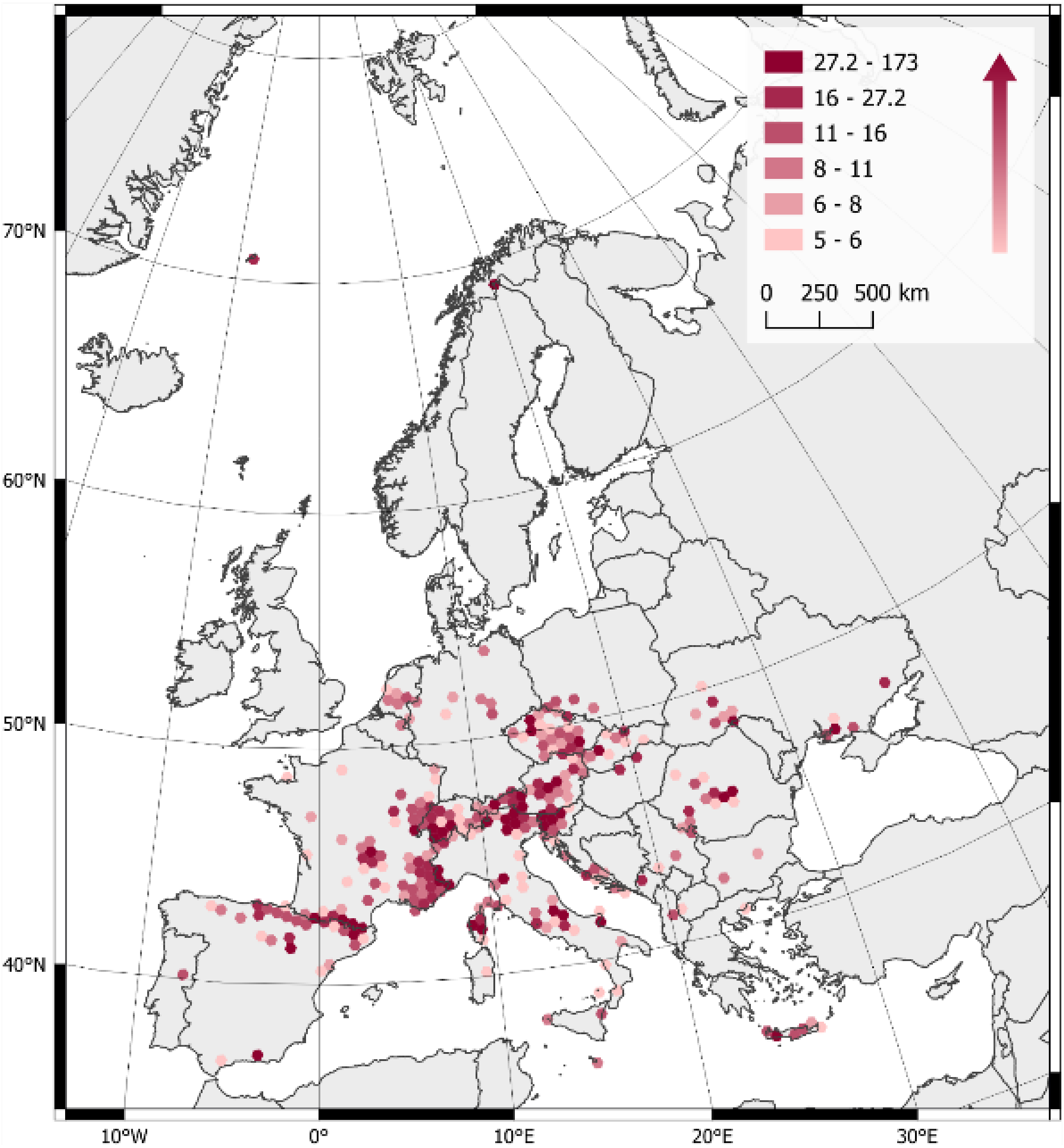

Fig. S1.7. Number of vegetation plots in the final data set for EUNIS habitat type U Inland Sparsely Vegetated Habitats. For the definition of the grid see Methods of the manuscript.
