# Supplementary material for: Parasitic plants in Europe: ecological niches and spatial patterns
Source: Plant Biol (Stuttg). 2025 Sep 18;27(7):1285–99. doi: 10.1111/plb.70099 (PMC12631522; doi:10.1111/plb.70099)
Supplement: Supplementary file 4 — Appendix S4. Additional information for functional types of parasitic plants and their occurrences in the species pool. [file PLB-27-1285-s006.pdf]

APPENDIX S4. Additional information for parasitic functional types and their occurrences in the species pool

Obligate root parasites	Euphytoid hemiparasites	Parasitic vines	Mistletoes

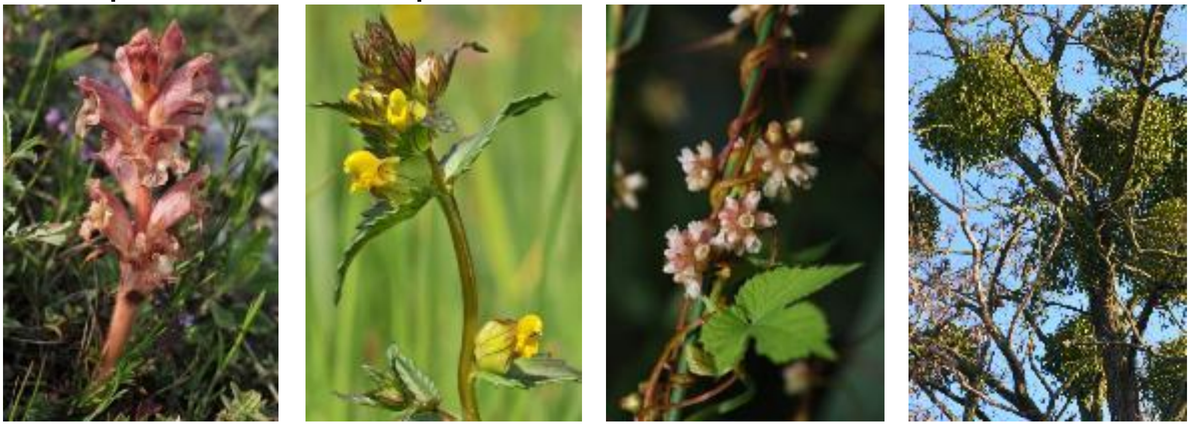

Orobanche alba	*Rhinanthus minor*	*Cuscuta* sp.	*Viscum album*

Fig. S4.1. Examples of the different parasitic plant functional types distinguished in the study. Photos: Jakub Těšitel.

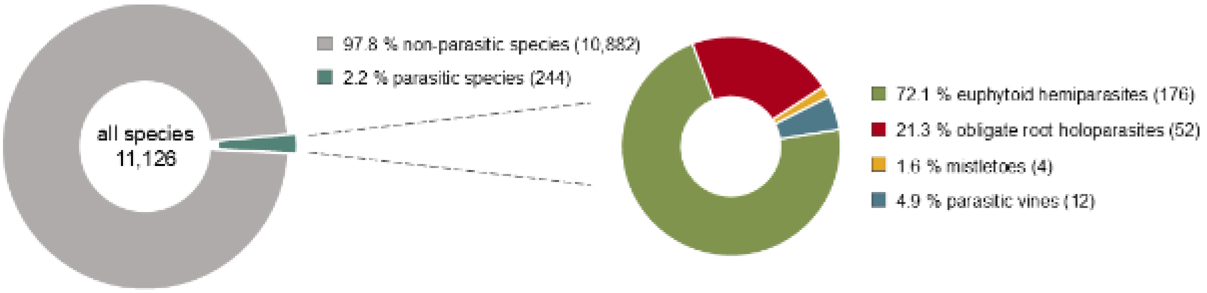

Fig. S4.2. The total and relative share of parasitic plants and the different functional types in the species pool of the final dataset. Percentage values do not add up to 100%, due to rounding.

Table S4.1. Direct comparison of the total and relative number of species of the parasitic plant functional types in our dataset to the occurrence in the FloraVeg database for Europe (Chytrý et al. 2024) and to the worldwide estimates according to Teixeira-Costa & Davis (2021).

	**Total n (%) in our dataset**	**Total n (%) in Europe (according to FloraVeg, 11/10/2024)**	**Total, n (%) worldwide**
**Euphytoid hemiparasites**	176 (72.1%)	330 (61.3%)	2469 (51.4%)
**Obligate root parasites**	51 (20.9%)	164 (30.5%)	371 (7.7%)
**Endoparasites**	1 (0.4%)	2 (0.4%)	78 (1.6%)
**Parasitic vines**	12 (4.9%)	34 (6.3%)	235 (5%)
**Mistletoes**	4 (1.6%)	8 (1.5%)	1647 (34.3%)
