# Supplementary material for: Parasitic plants in Europe: ecological niches and spatial patterns
Source: Plant Biol (Stuttg). 2025 Sep 18;27(7):1285–99. doi: 10.1111/plb.70099 (PMC12631522; doi:10.1111/plb.70099)
Supplement: Supplementary file 5 — Appendix S5. Results of the RDA forward selection. [file PLB-27-1285-s010.pdf]

APPENDIX S5. Results of the RDA forward selection

We performed an RDA forward selection for the selection of climatic variables to use in our analysis (bio1-19, pet_penman_mean). Variables were log-transformed according to TABLE S3.4. We used the climatic values of the plots of the whole dataset for which all variables were available as response variables. We performed forward selection using the *'vegan'* package in R, version 4.3.0 (2023-04-21; R Core Team., 2023). We used standardised scaling within the *rda* function. Mean daily mean air temperatures of the warmest quarter (bio10), Temperature seasonality (bio4), Annual precipitation amount (bio12), Precipitation seasonality (bio15), Mean diurnal air temperature range (bio2), Mean monthly precipitation amount of the warmest quarter (bio18), and Mean monthly potential evapotranspiration (pen_penman) were selected based on their AIC value, while we prioritised those that best represented conditions during the main growing season for most plant species if two or more variables showed a similar value. The constrained axis explained 95.6% of the total variability in the data (R^2adj).

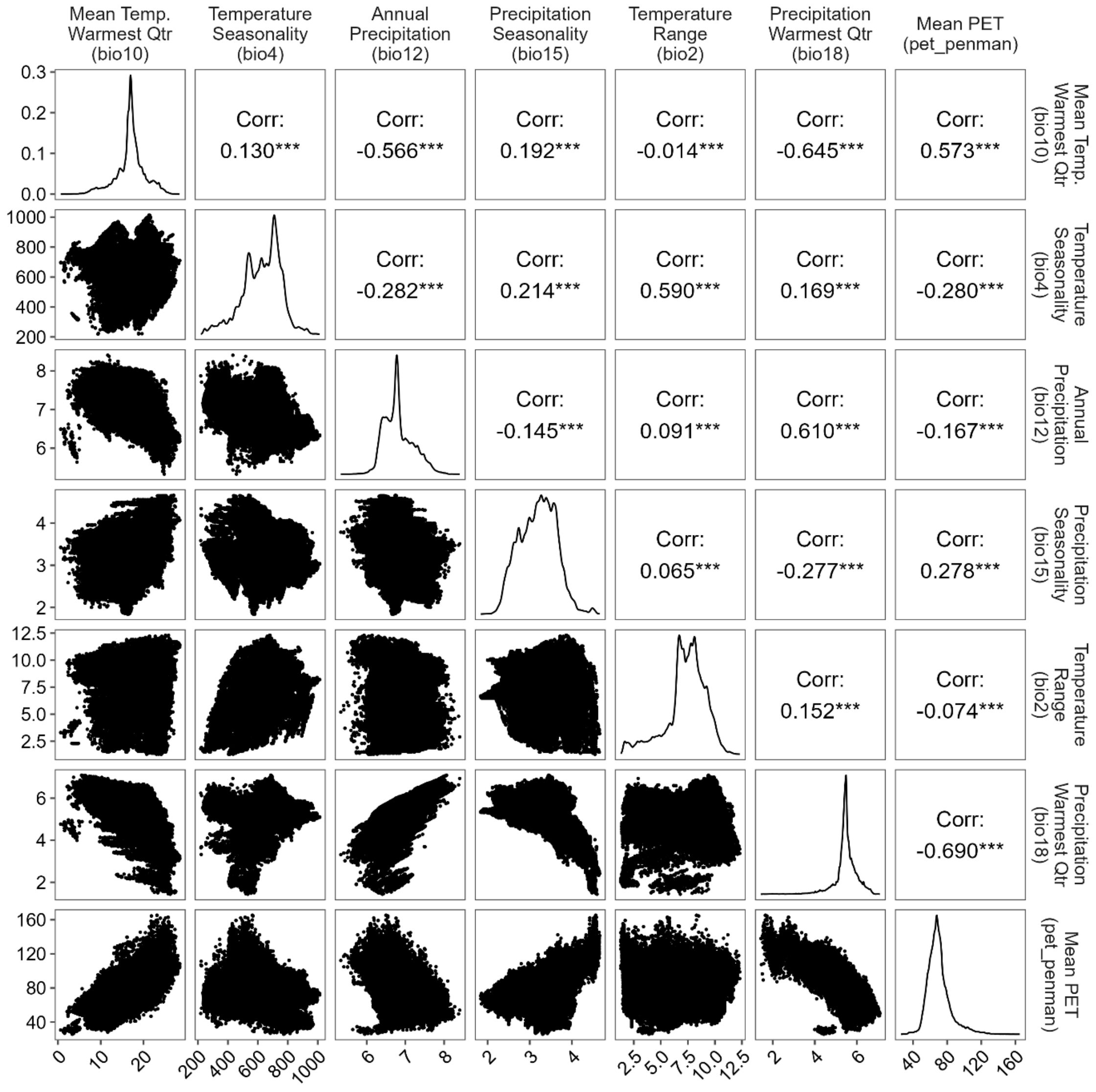

Fig. S5.1. Pairwise relationships between climatic variables used in the analysis. The plot presents scatterplots in the lower triangle (including smoothed trend lines (LOESS), density plots (diagonal panels) using LOESS smoothing, and Pearson correlation coefficients (upper triangle, scaled from -1 to 1) for selected climatic variables (see Table. S3.4 for more information).
