# Supplementary material for: Parasitic plants in Europe: ecological niches and spatial patterns
Source: Plant Biol (Stuttg). 2025 Sep 18;27(7):1285–99. doi: 10.1111/plb.70099 (PMC12631522; doi:10.1111/plb.70099)
Supplement: Supplementary file 6 — Appendix S6. Maps for the mean relative cover of parasitic functional types in EUNIS habitat types. [file PLB-27-1285-s007.pdf]

APPENDIX S6. Maps for the mean relative cover of parasitic functional types in EUNIS habitat types

Figures S6.1 to S6.21 show the mean relative cover of parasitic plants in Europe according to the functional type and the EUNIS habitat type.

Fig. S6.1. Euphytoid hemiparasites in M Coastal Saltmarshes
Fig. S6.2. Euphytoid hemiparasites in N Coastal Sand and Cliff habitats
Fig. S6.3. Euphytoid hemiparasites in Q Wetlands
Fig. S6.4. Euphytoid hemiparasites in R Grasslands
Fig. S6.5. Euphytoid hemiparasites in S Heathlands, Scrub and Tundra
Fig. S6.6. Euphytoid hemiparasites in T Forests
Fig. S6.7. Euphytoid hemiparasites in U Inland Sparsely Vegetated habitat types
Fig. S6.8. Obligate root parasites in M Coastal Saltmarshes
Fig. S6.9. Obligate root parasites in N Coastal Sand and Cliff habitats
Fig. S6.10. Obligate root parasites in Q Wetlands
Fig. S6.11. Obligate root parasites in R Grasslands
Fig. S6.12. Obligate root parasites in S Heathlands, Scrub and Tundra
Fig. S6.13. Obligate root parasites in T Forests
Fig. S6.14. Obligate root parasites in U Inland Sparsely Vegetated habitat types
Fig. S6.15. Parasitic vines in M Coastal Saltmarshes
Fig. S6.16. Parasitic vines in N Coastal Sand and Cliff habitats
Fig. S6.17. Parasitic vines in Q Wetlands
Fig. S6.18. Parasitic vines in R Grasslands
Fig. S6.19. Parasitic vines in S Heathlands, Scrub and Tundra
Fig. S6.20. Parasitic vines in T Forests
Fig. S6.21. Parasitic vines in U Inland Sparsely Vegetated habitat types

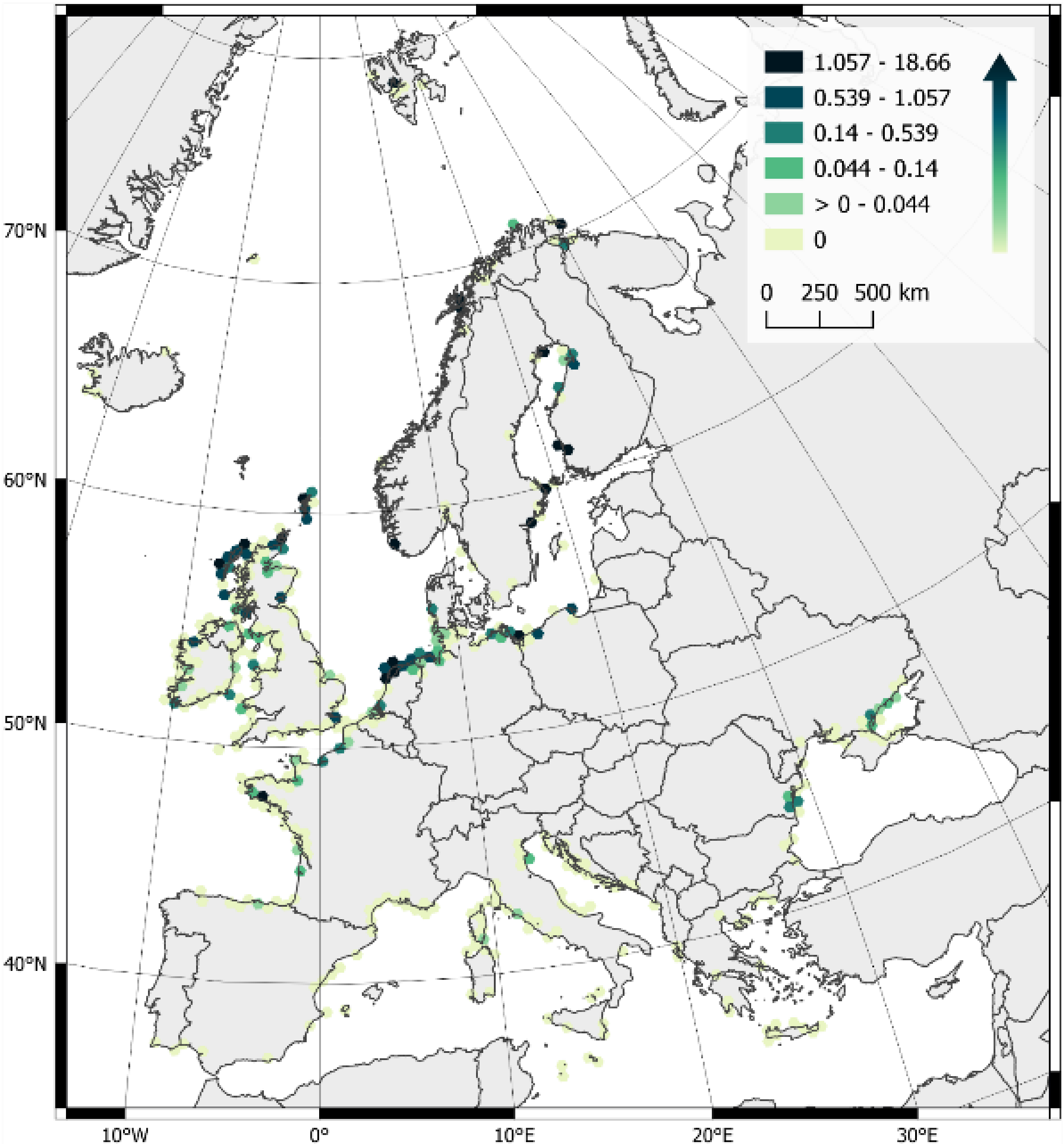

Fig. S6.1. Mean relative cover of euphytoid hemiparasites per grid cell for the EUNIS habitat type M Coastal Saltmarshes. Grid cells are 50 km in latitudinal extent. Values are only calculated for grid cells containing at least 5 plots.

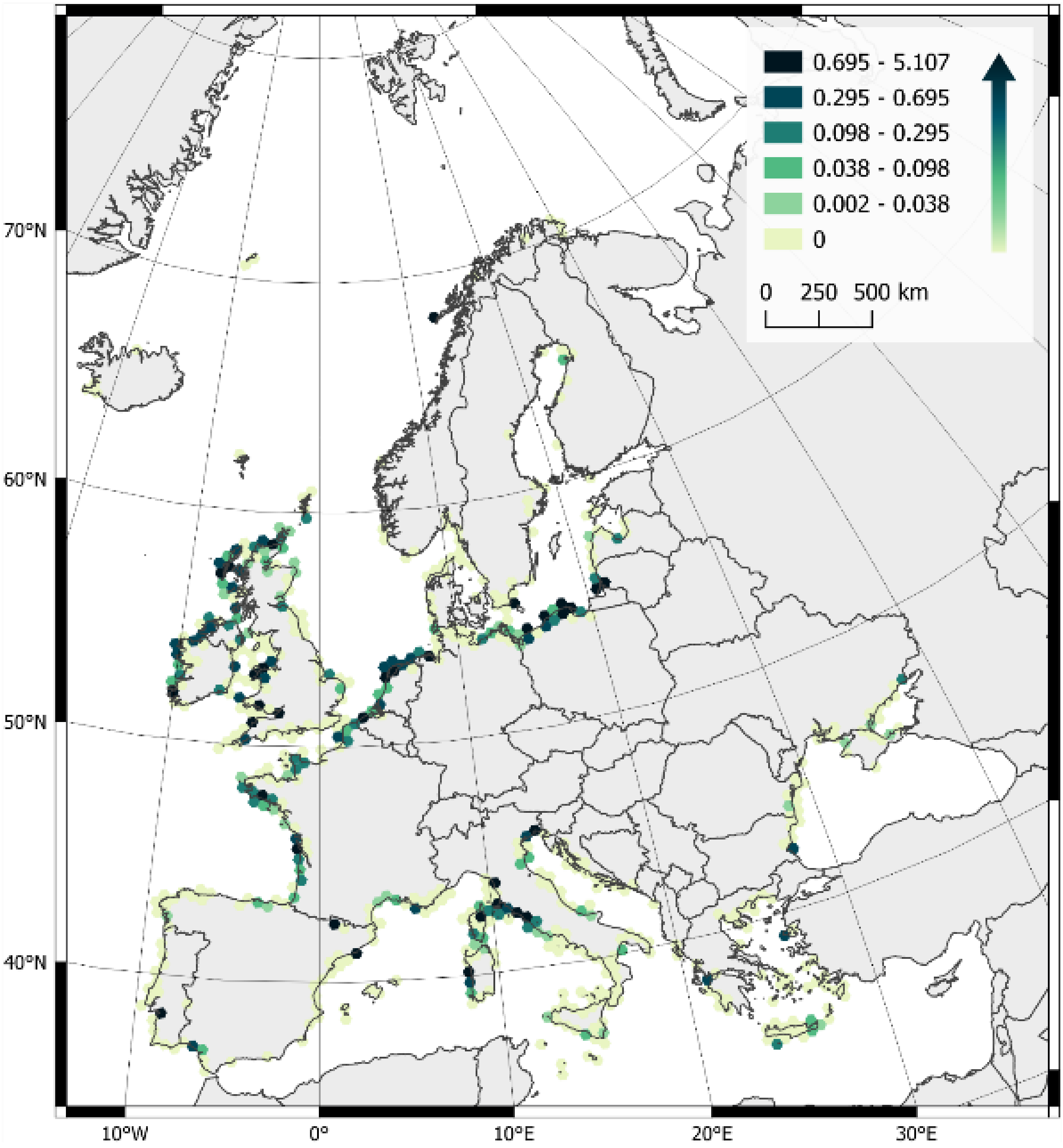

Fig. S6.2. Mean relative cover of euphytoid hemiparasites per grid cell for the EUNIS habitat type N Coastal Sand and Cliff habitats. Grid cells are 50 km in latitudinal extent. Values are only calculated for grid cells containing at least 5 plots.

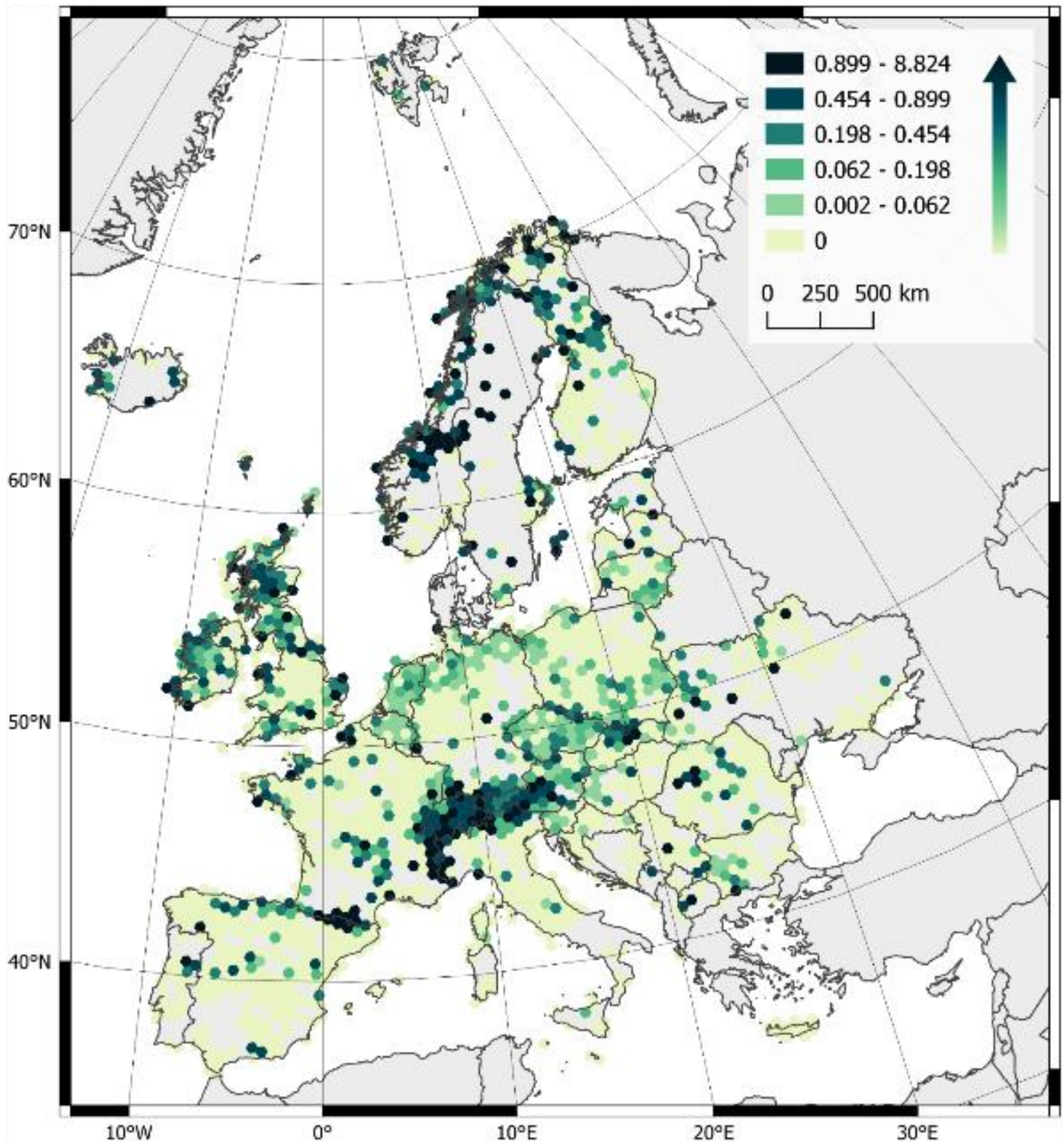

Fig. S6.3. Mean relative cover of euphytoid hemiparasites per grid cell for the EUNIS habitat type Q Wetlands. Grid cells are 50 km in latitudinal extent. Values are only calculated for grid cells containing at least 5 plots.

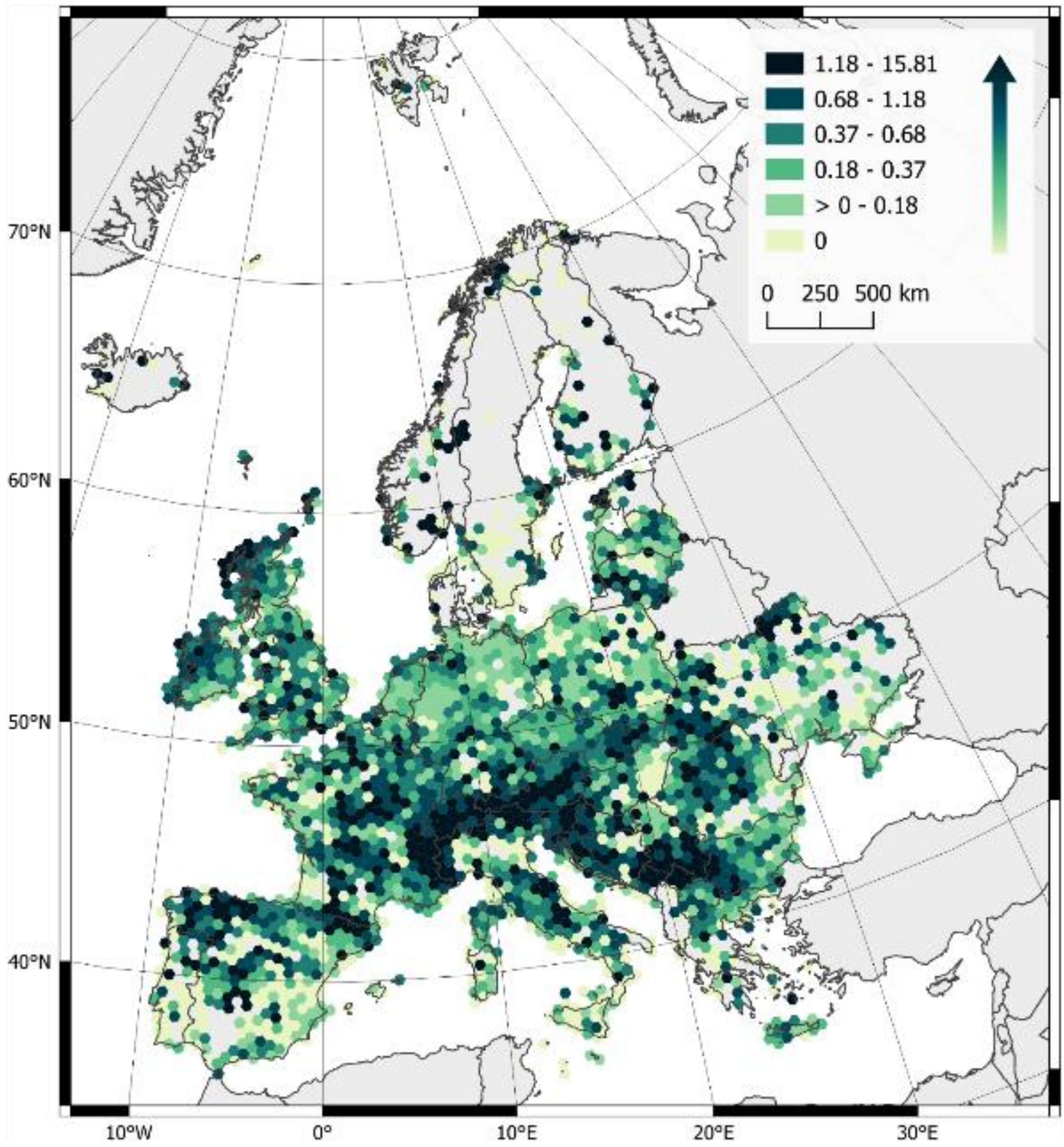

Fig. S6.4. Mean relative cover of euphytoid hemiparasites per grid cell for the EUNIS habitat type R Grasslands. Grid cells are 50 km in latitudinal extent. Values are only calculated for grid cells containing at least 5 plots.

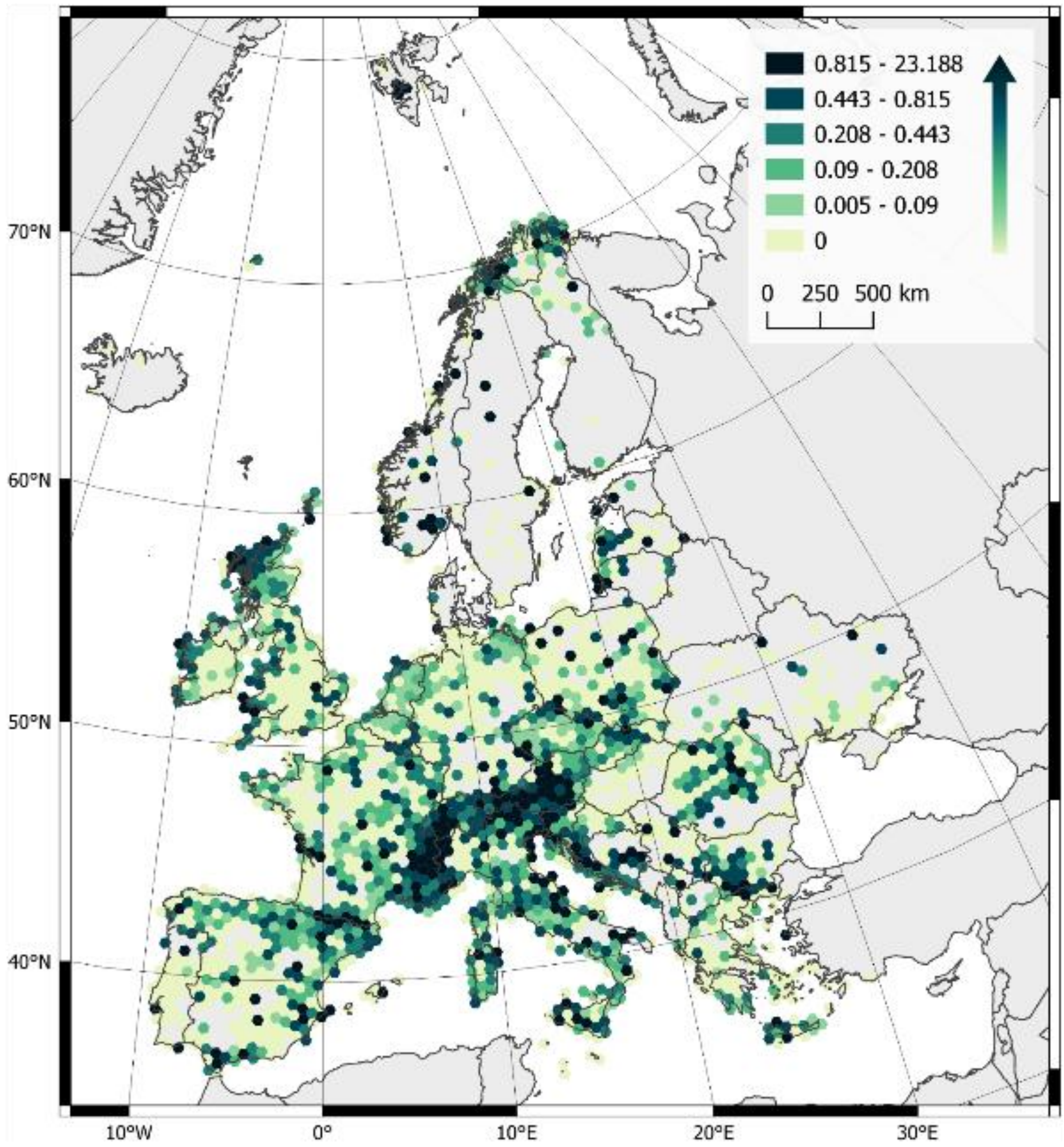

Fig. S6.5. Mean relative cover of euphytoid hemiparasites per grid cell for the EUNIS habitat type S Heathlands, Scrub and Tundra. Grid cells are 50 km in latitudinal extent. Values are only calculated for grid cells containing at least 5 plots.

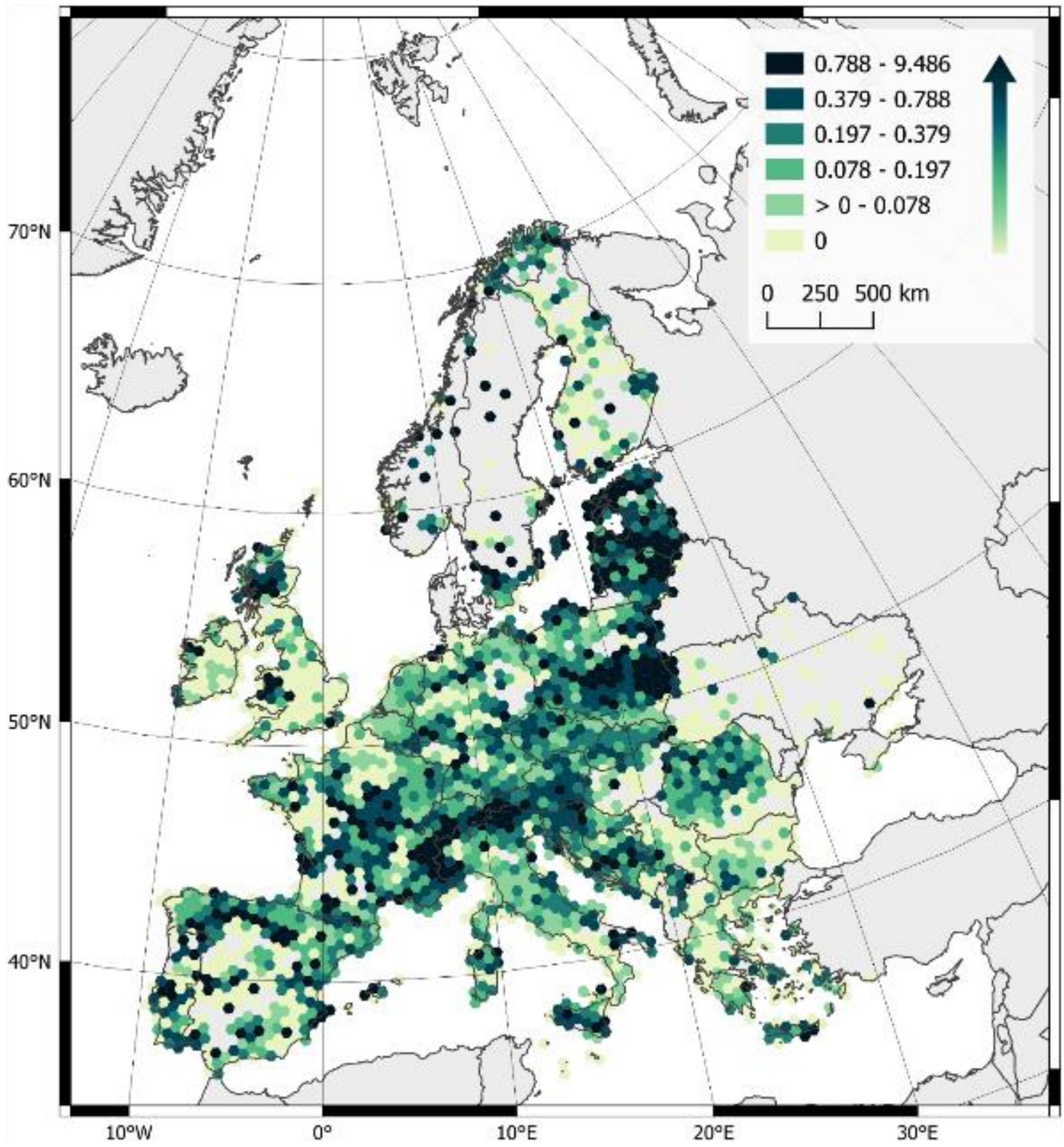

Fig. S6.6. Mean relative cover of euphytoid hemiparasites per grid cell for the EUNIS habitat type T Forests. Grid cells are 50 km in latitudinal extent. Values are only calculated for grid cells containing at least 5 plots.

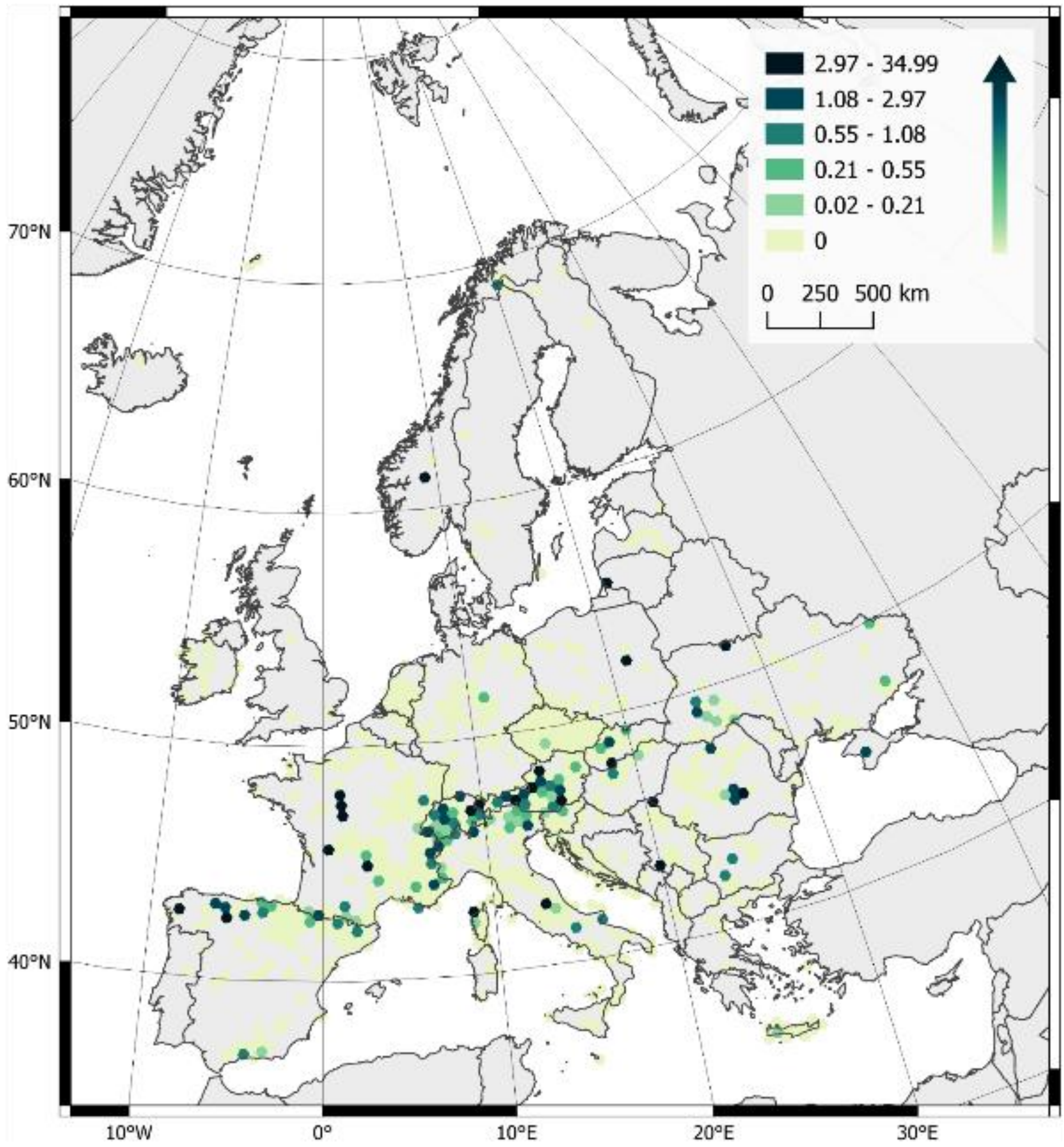

Fig. S6.7. Mean relative cover of euphytoid hemiparasites per grid cell for the EUNIS habitat type U Inland Sparsely Vegetated habitat types. Grid cells are 50 km in latitudinal extent. Values are only calculated for grid cells containing at least 5 plots.

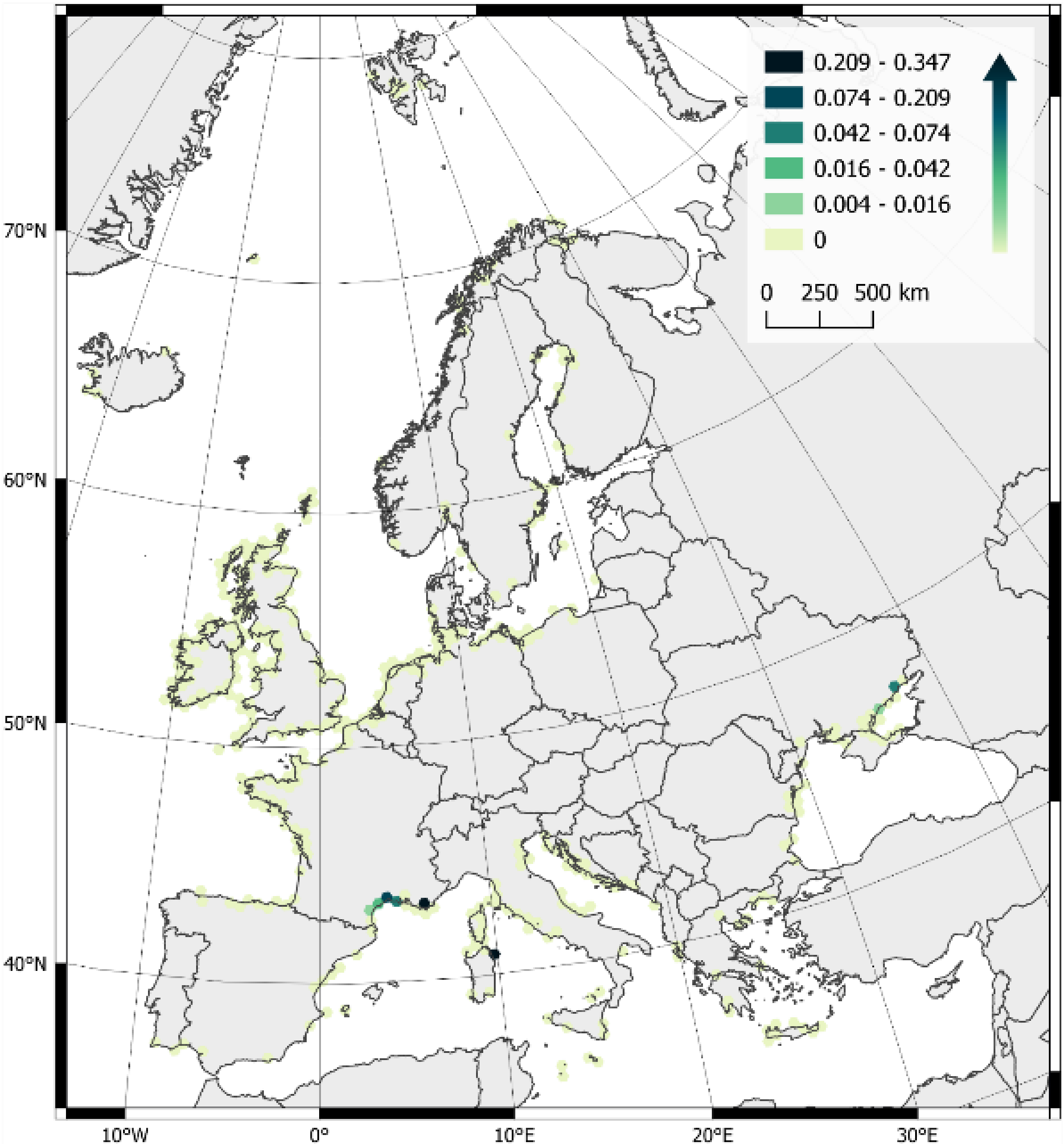

Fig. S6.8. Mean relative cover of obligate root parasites per grid cell for the EUNIS habitat type M Coastal Saltmarshes. Grid cells are 50 km in latitudinal extent. Values are only calculated for grid cells containing at least 5 plots.

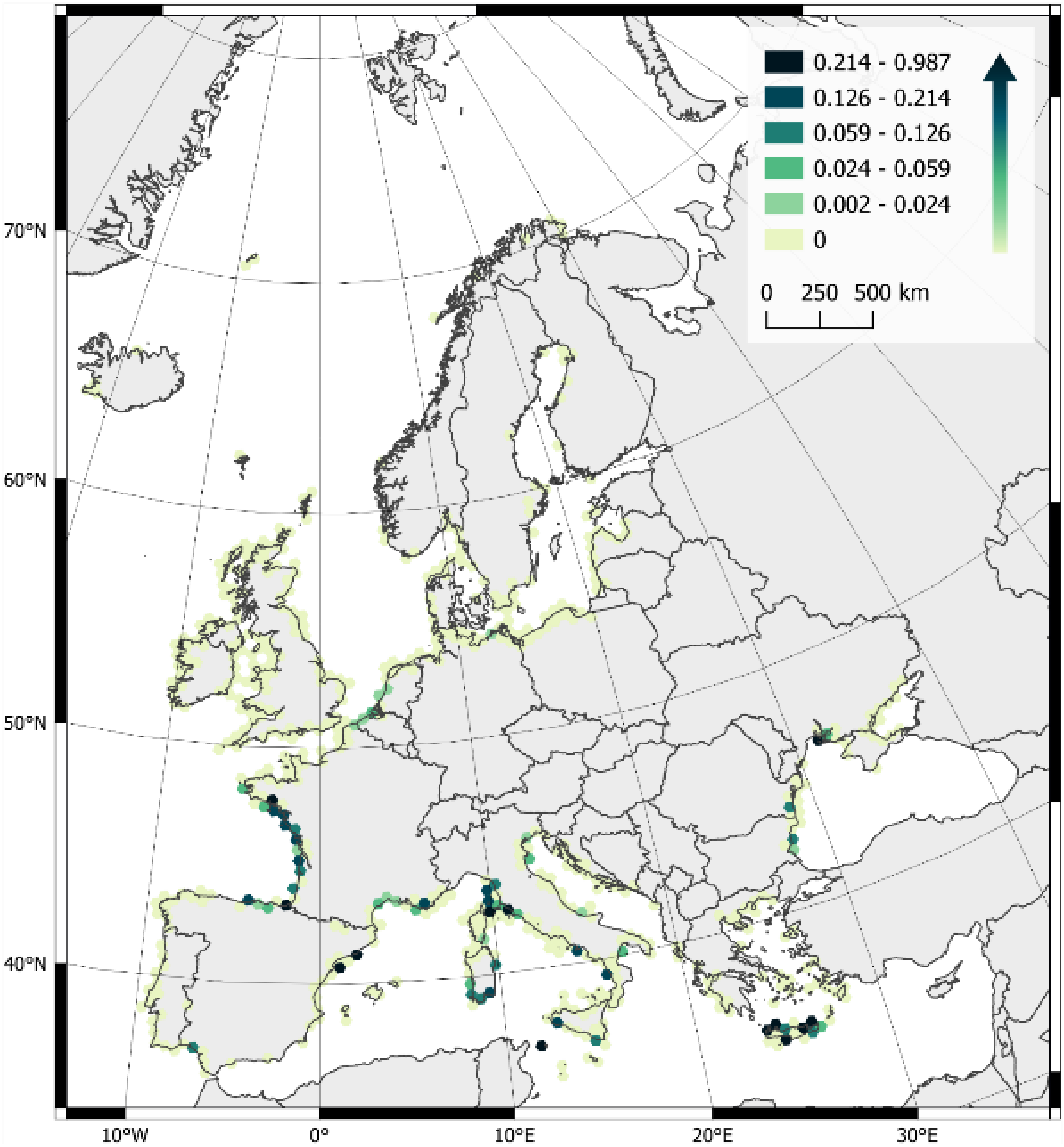

Fig. S6.9. Mean relative cover of obligate root parasites per grid cell for the EUNIS habitat type N Coastal Sand and Cliff habitats. Grid cells are 50 km in latitudinal extent. Values are only calculated for grid cells containing at least 5 plots.

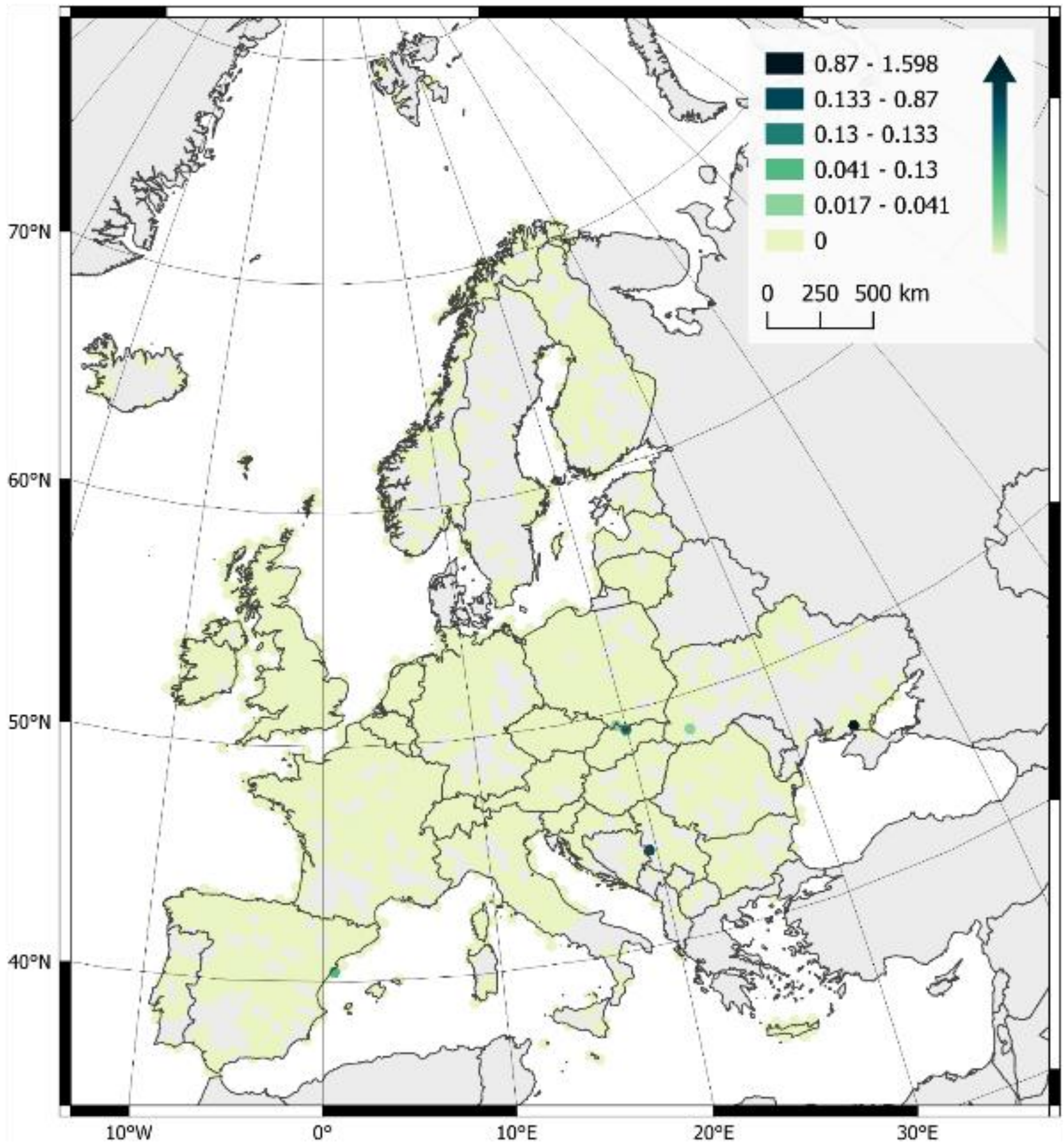

Fig. S6.10. Mean relative cover of obligate root parasites per grid cell for the EUNIS habitat type Q Wetlands. Grid cells are 50 km in latitudinal extent. Values are only calculated for grid cells containing at least 5 plots.

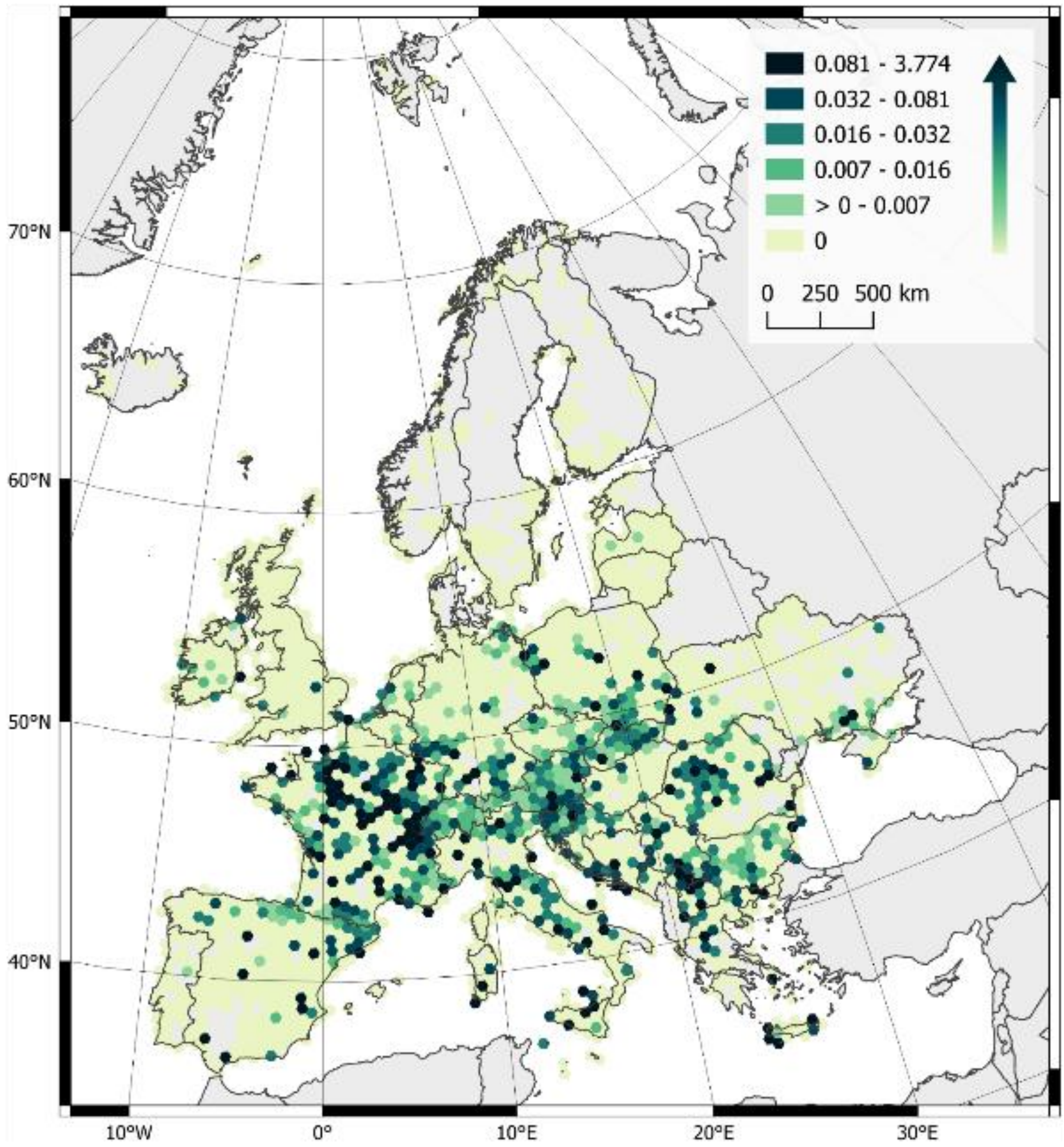

Fig. S6.11. Mean relative cover of obligate root parasites per grid cell for the EUNIS habitat type R Grasslands. Grid cells are 50 km in latitudinal extent. Values are only calculated for grid cells containing at least 5 plots.

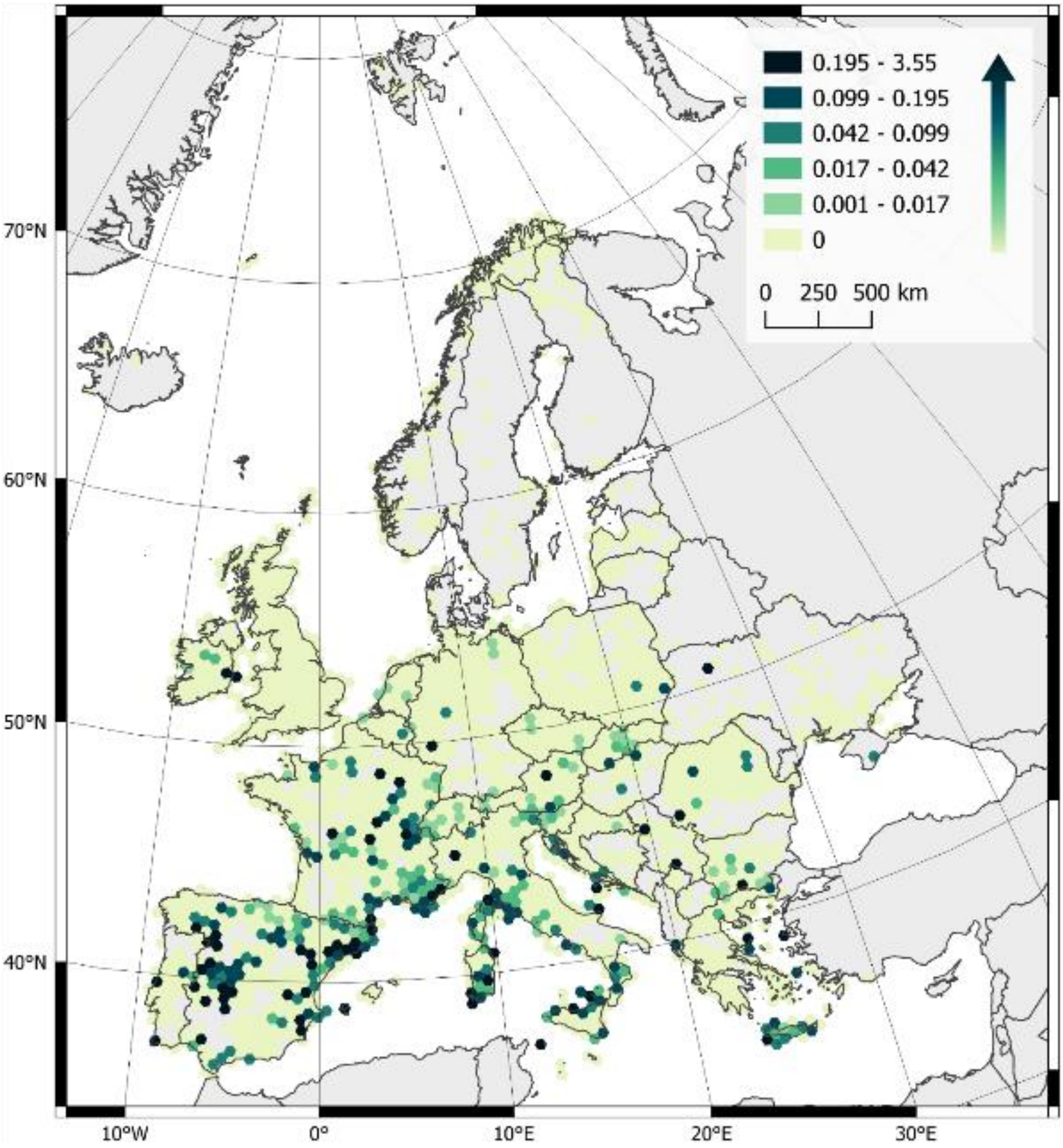

Fig. S6.12. Mean relative cover of obligate root parasites per grid cell for the EUNIS habitat type S Heathlands, Scrub and Tundra. Grid cells are 50 km in latitudinal extent. Values are only calculated for grid cells containing at least 5 plots.

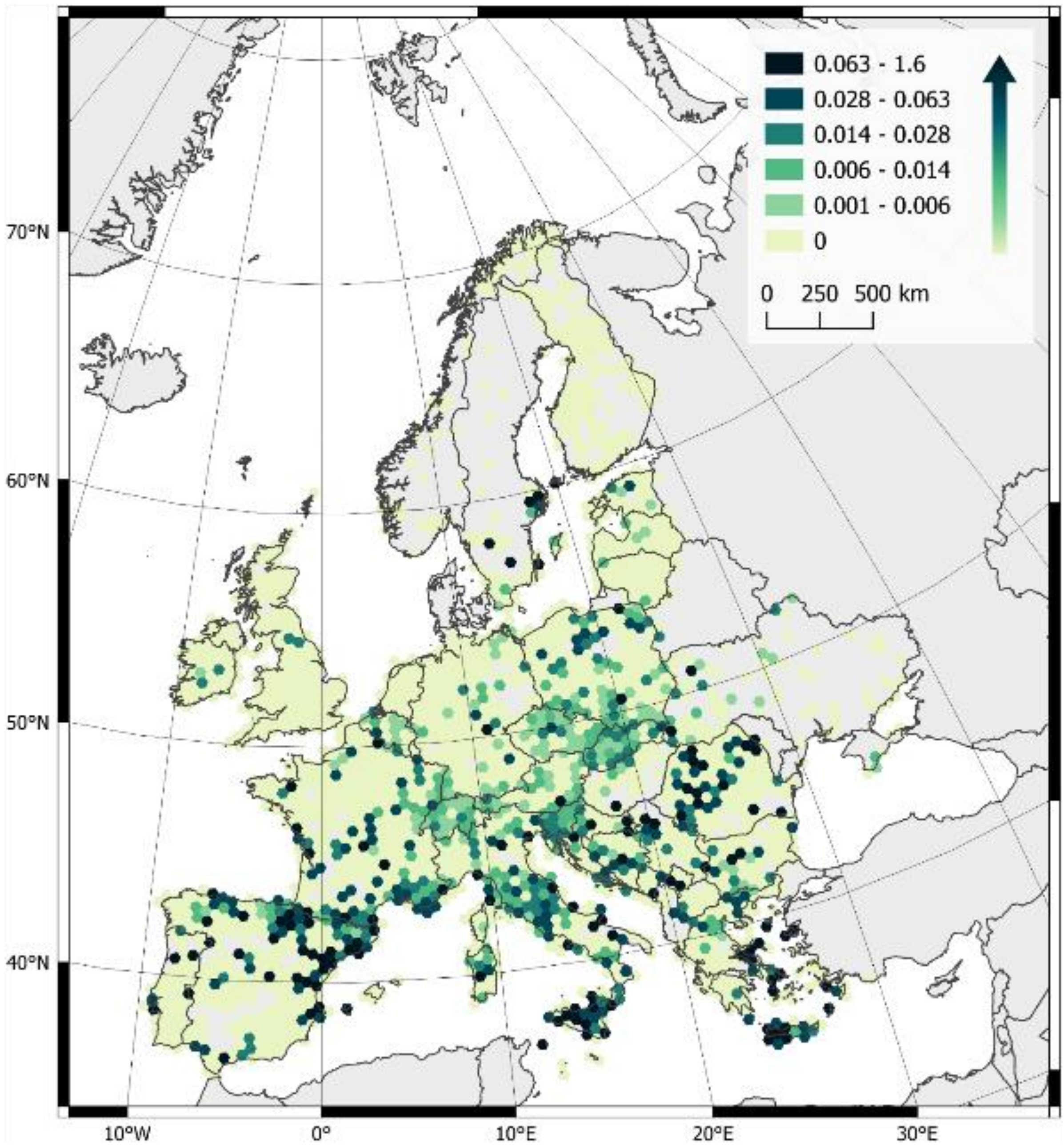

Fig. S6.13. Mean relative cover of obligate root parasites per grid cell for the EUNIS habitat type T Forests. Grid cells are 50 km in latitudinal extent. Values are only calculated for grid cells containing at least 5 plots.

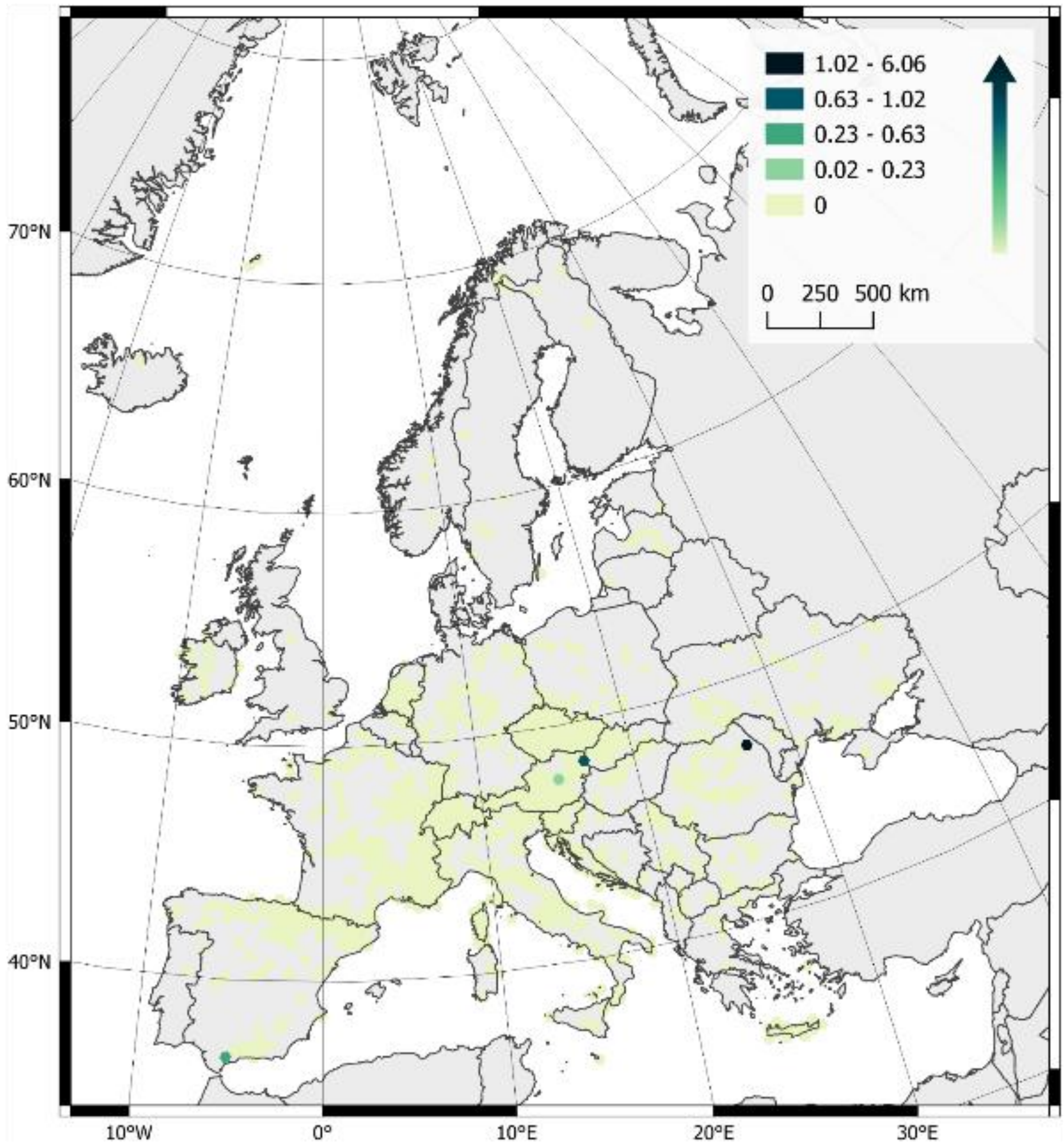

Fig. S6.14. Mean relative cover of obligate root parasites per grid cell for the EUNIS habitat type U Inland Sparsely Vegetated habitat types. Grid cells are 50 km in latitudinal extent. Values are only calculated for grid cells containing at least 5 plots.

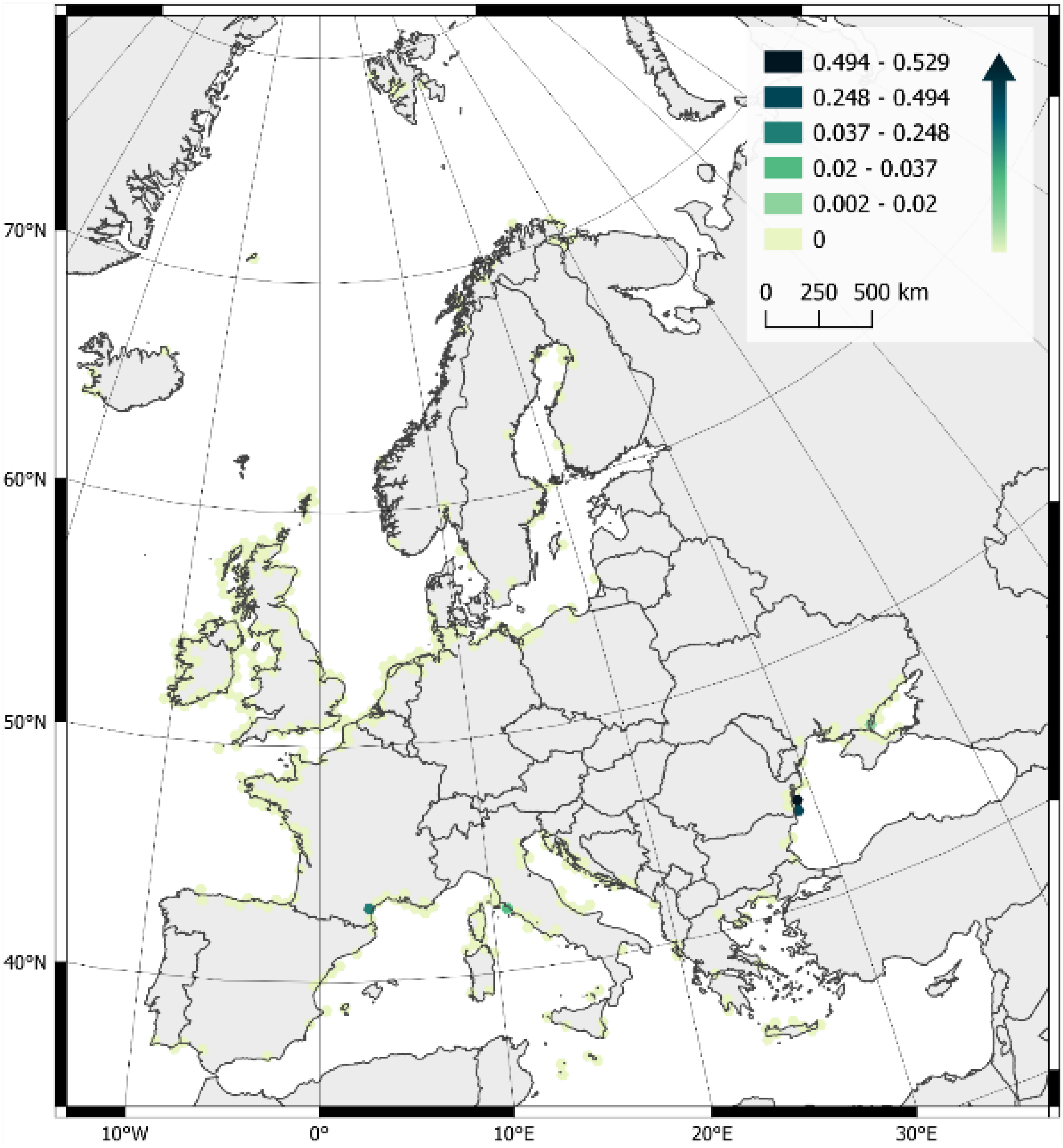

Fig. S6.15. Mean relative cover of parasitic vines per grid cell for the EUNIS habitat type M Coastal Saltmarshes. Grid cells are 50 km in latitudinal extent. Values are only calculated for grid cells containing at least 5 plots.

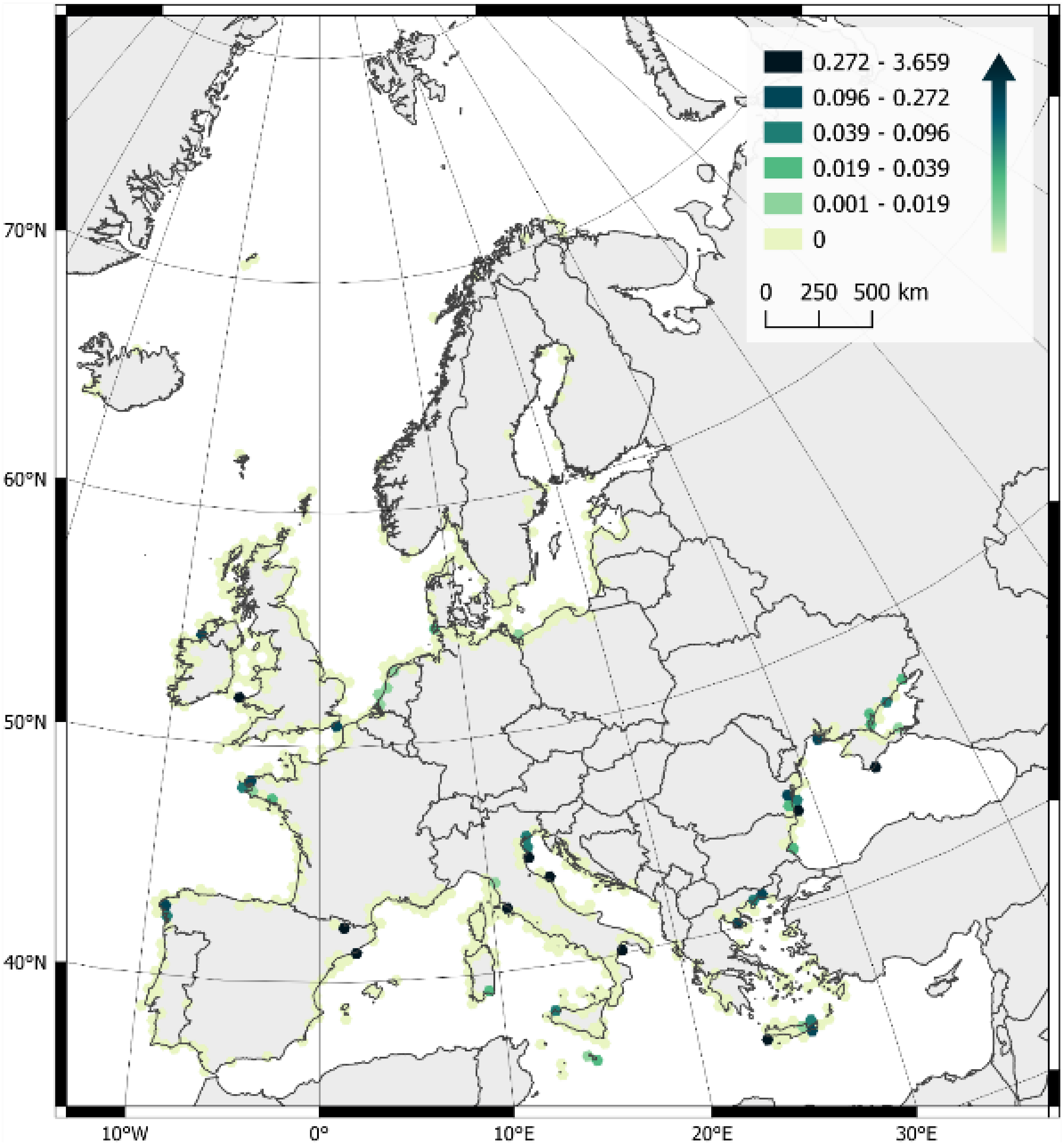

Fig. S6.16. Mean relative cover of parasitic vines per grid cell for the EUNIS habitat type N Coastal Sand and Cliff habitats. Grid cells are 50 km in latitudinal extent. Values are only calculated for grid cells containing at least 5 plots.

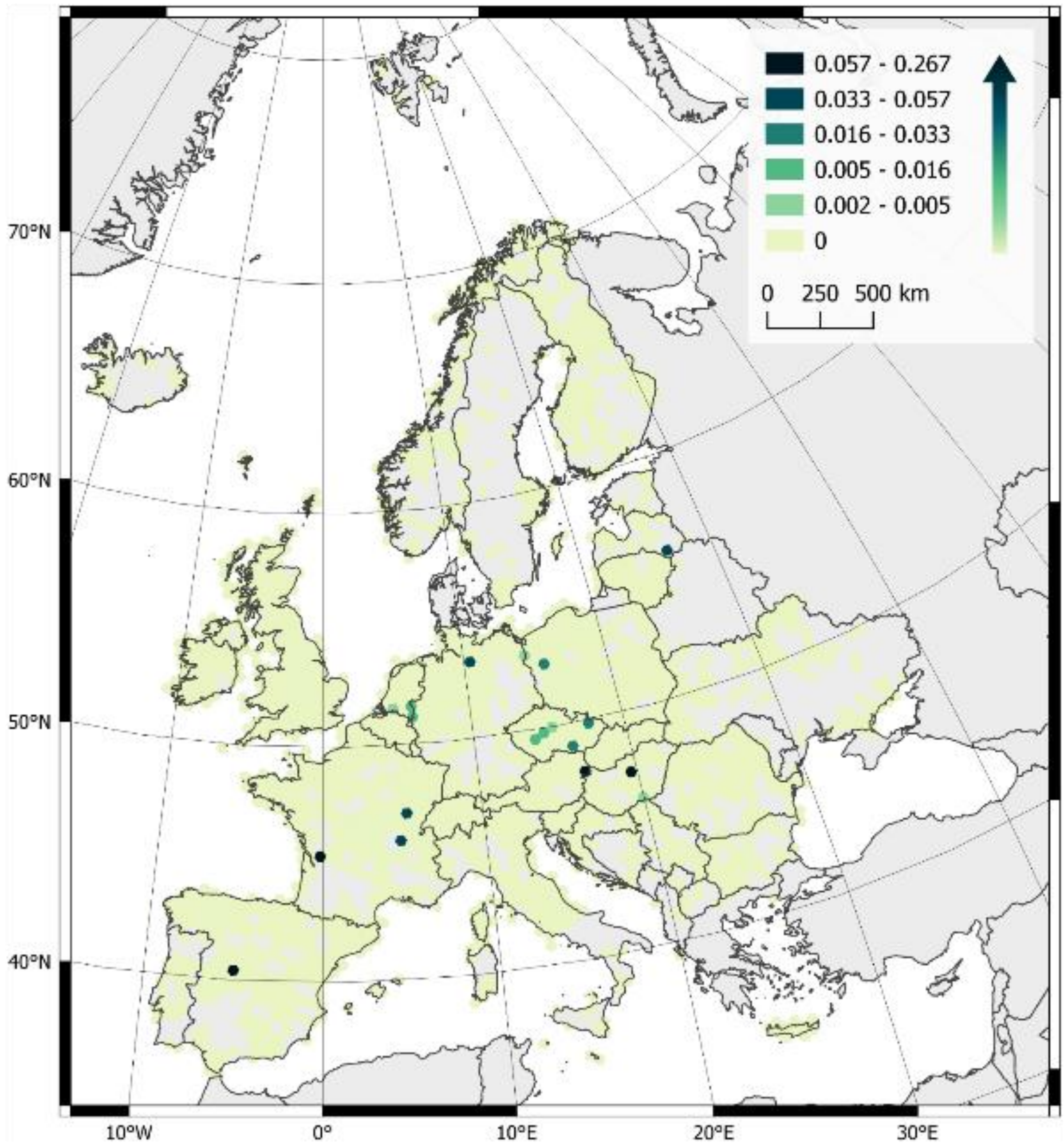

Fig. S6.17. Mean relative cover of parasitic vines per grid cell for the EUNIS habitat type Q Wetlands. Grid cells are 50 km in latitudinal extent. Values are only calculated for grid cells containing at least 5 plots.

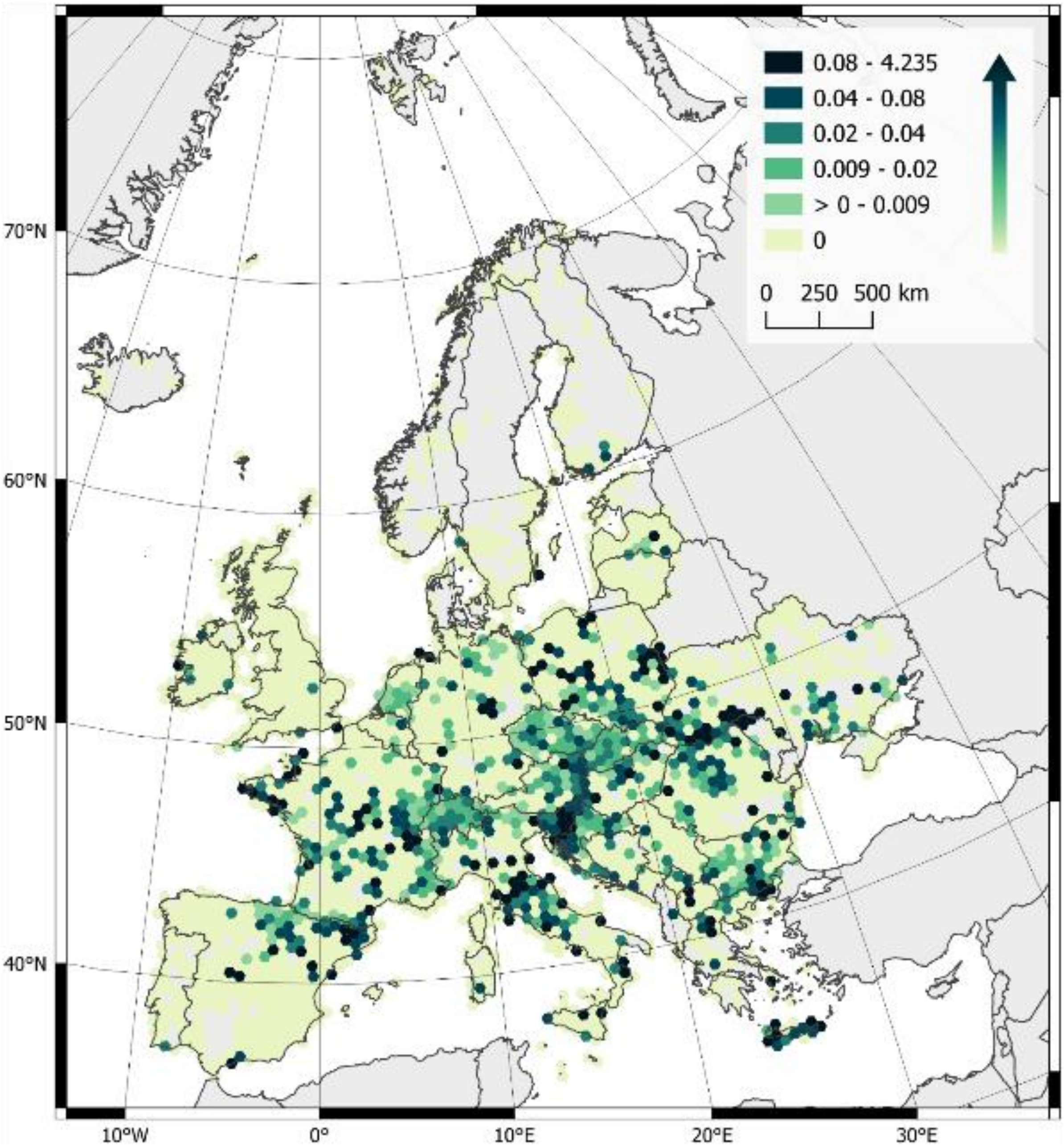

Fig. S6.18. Mean relative cover of parasitic vines per grid cell for the EUNIS habitat type R Grasslands. Grid cells are 50 km in latitudinal extent. Values are only calculated for grid cells containing at least 5 plots.

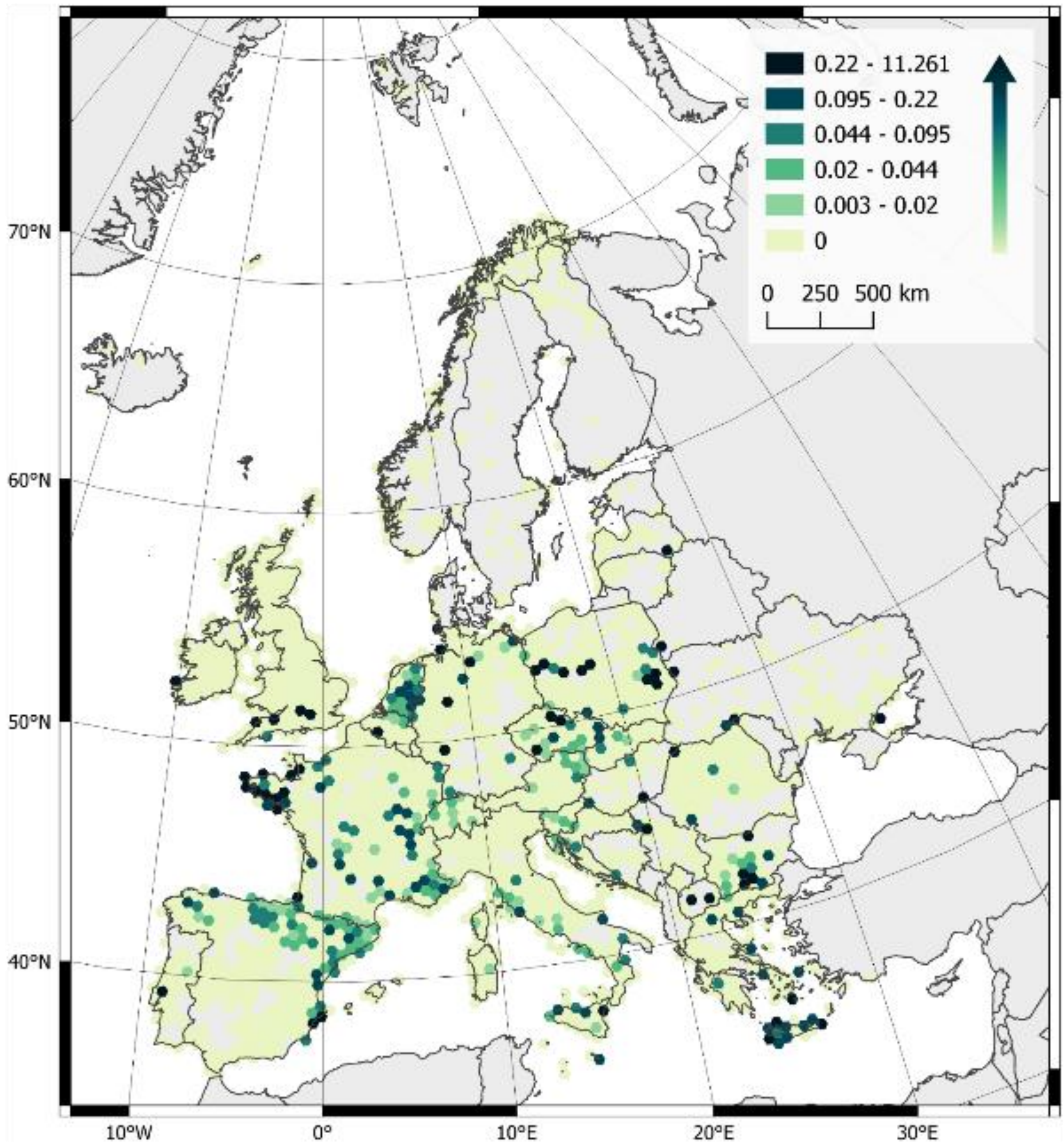

Fig. S6.19. Mean relative cover of parasitic vines per grid cell for the EUNIS habitat type S Heathlands, Scrub and Tundra. Grid cells are 50 km in latitudinal extent. Values are only calculated for grid cells containing at least 5 plots.

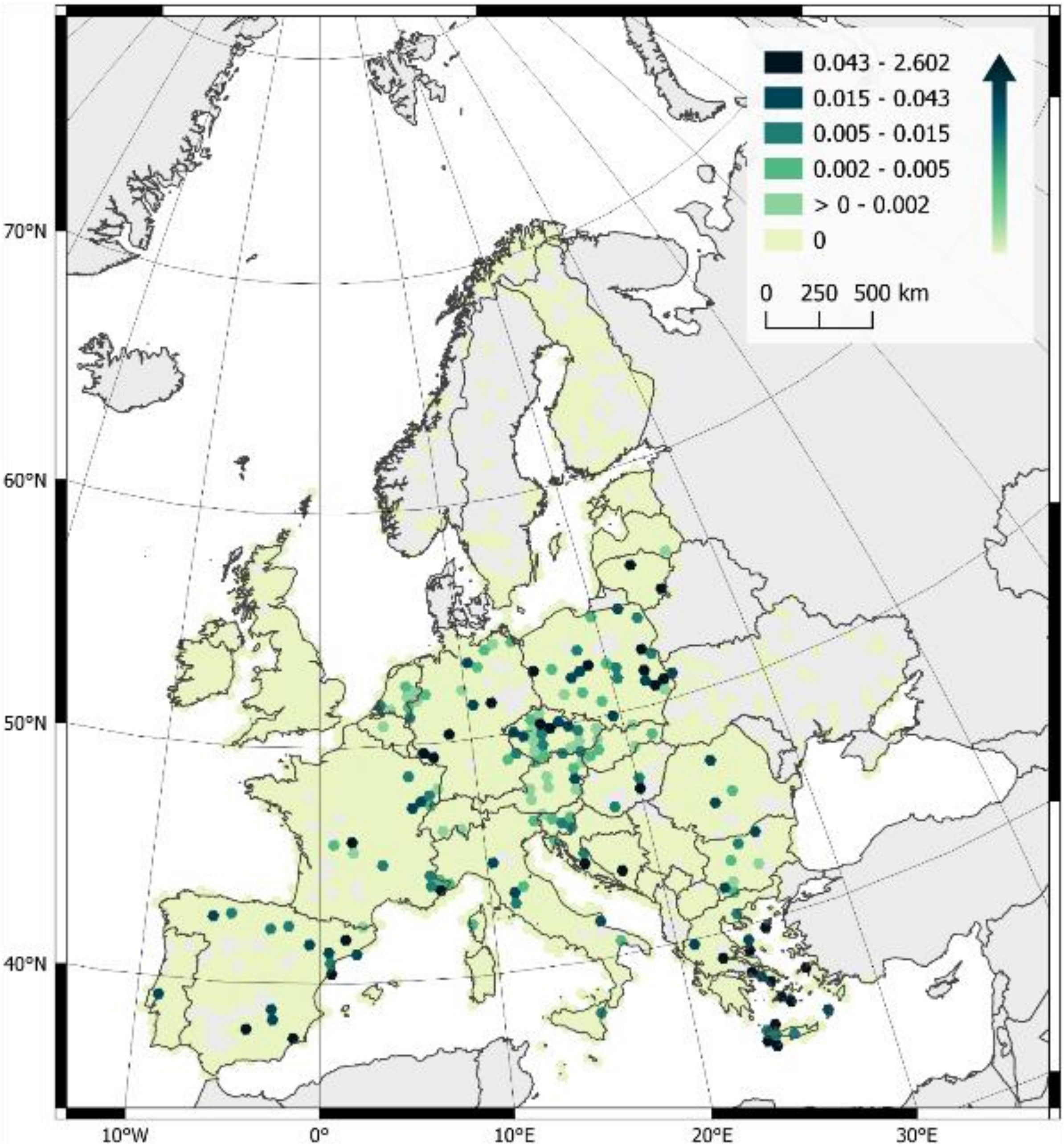

Fig. S6.20. Mean relative cover of parasitic vines per grid cell for the EUNIS habitat type T Forests. Grid cells are 50 km in latitudinal extent. Values are only calculated for grid cells containing at least 5 plots.

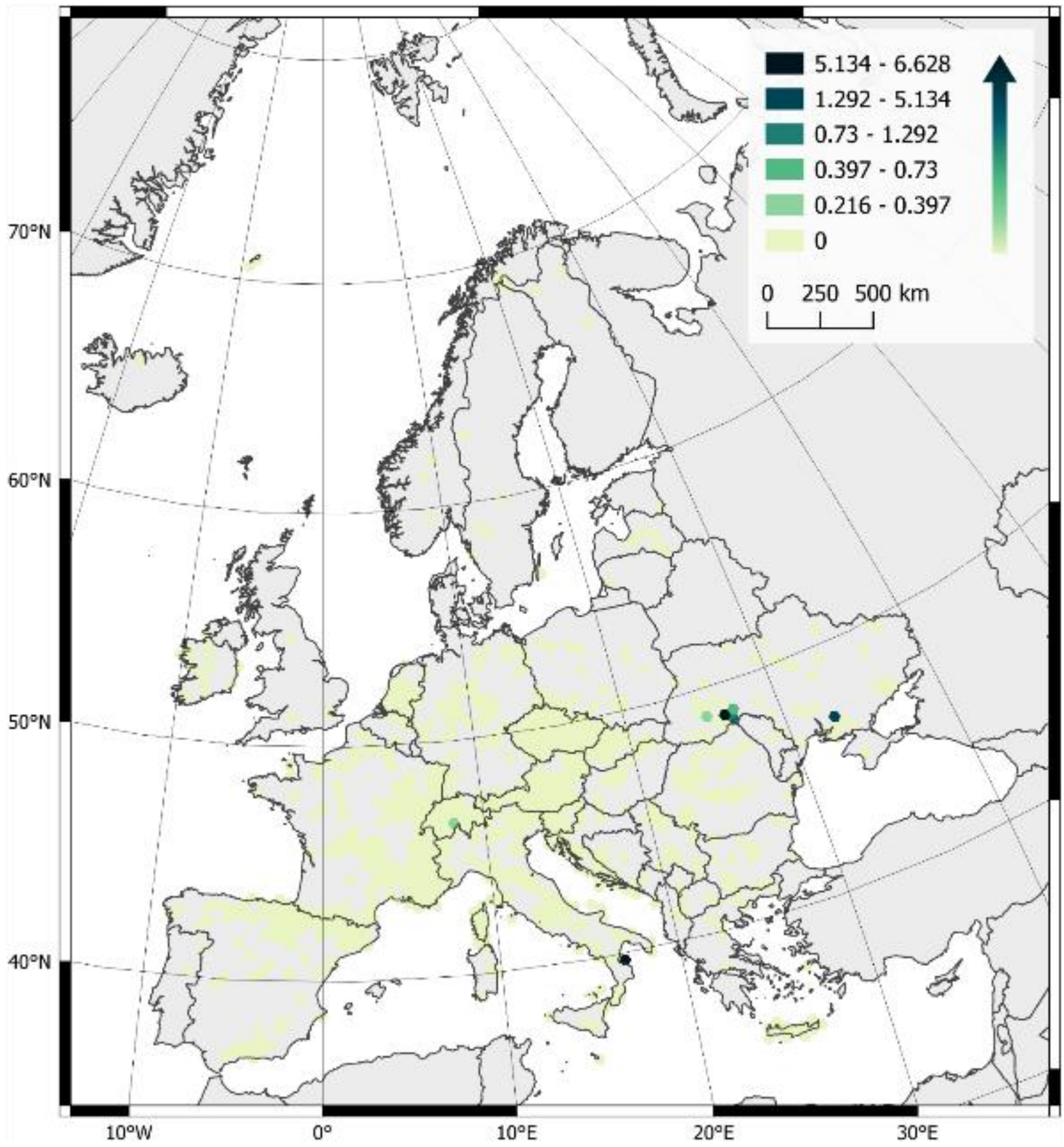

Fig. S6.21. Mean relative cover of parasitic vines per grid cell for the EUNIS habitat type U Inland Sparsely Vegetated habitat types. Grid cells are 50 km in latitudinal extent. Values are only calculated for grid cells containing at least 5 plots.
