# Supplementary material for: Parasitic plants in Europe: ecological niches and spatial patterns
Source: Plant Biol (Stuttg). 2025 Sep 18;27(7):1285–99. doi: 10.1111/plb.70099 (PMC12631522; doi:10.1111/plb.70099)
Supplement: Supplementary file 7 — Appendix S7. Maps for the environmental variables used in the study. [file PLB-27-1285-s002.pdf]

APPENDIX S7. Maps for the environmental variables used in the study

In the same way that we prepared maps for the distribution of parasitic plants, we also prepared maps to give an overview of the predictors used in this study. We used the same grid as for the other maps and calculated the mean values for each environmental variable over all plots in one grid cell. Divergent from the original method we did not exclude cells with less than 5 plots. The colour scale follows the Equal Count (Quantile) method implemented in QGIS Desktop 3.32.1.

Fig. S7.1. Mean diurnal air temperature range (°C)
Fig. S7.2. Temperature seasonality (°C/100)
Fig. S7.3. Mean daily mean air temperatures of the warmest quarter (°C)
Fig. S7.4. Annual precipitation amount (mm)
Fig. S7.5. Precipitation seasonality (mm)
Fig. S7.6. Mean monthly precipitation amount of the warmest quarter (mm)
Fig. S7.7. Mean monthly potential evapotranspiration ($kg/m^2/month$)
Fig. S7.8. Terrain Ruggedness Index (m)

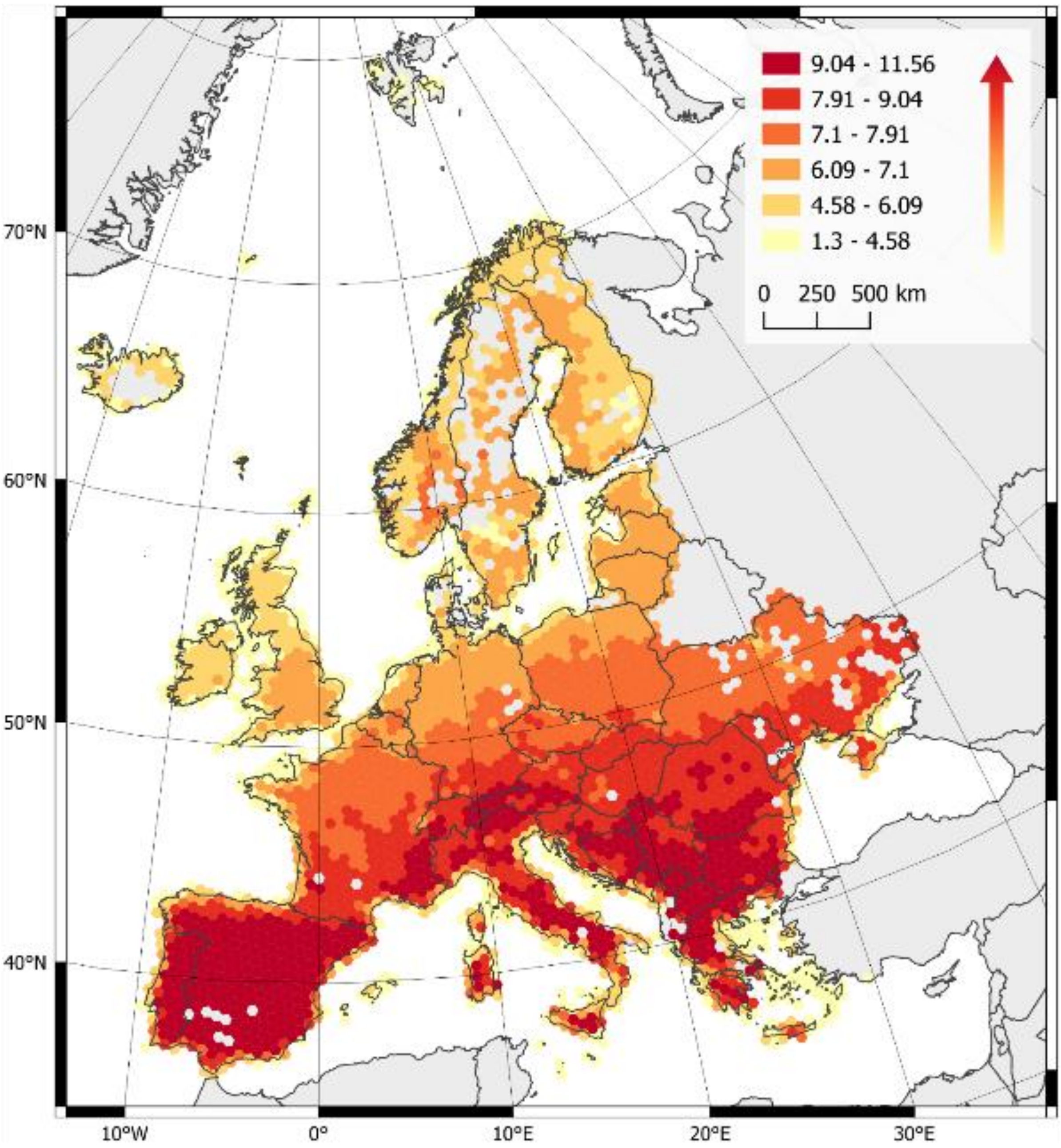

Fig. S7.1. Mean values of the Mean diurnal air temperature range in °C per grid cell. Grid cells are 50 km in latitudinal extent. For more information on the environmental predictors see Appendix S3.

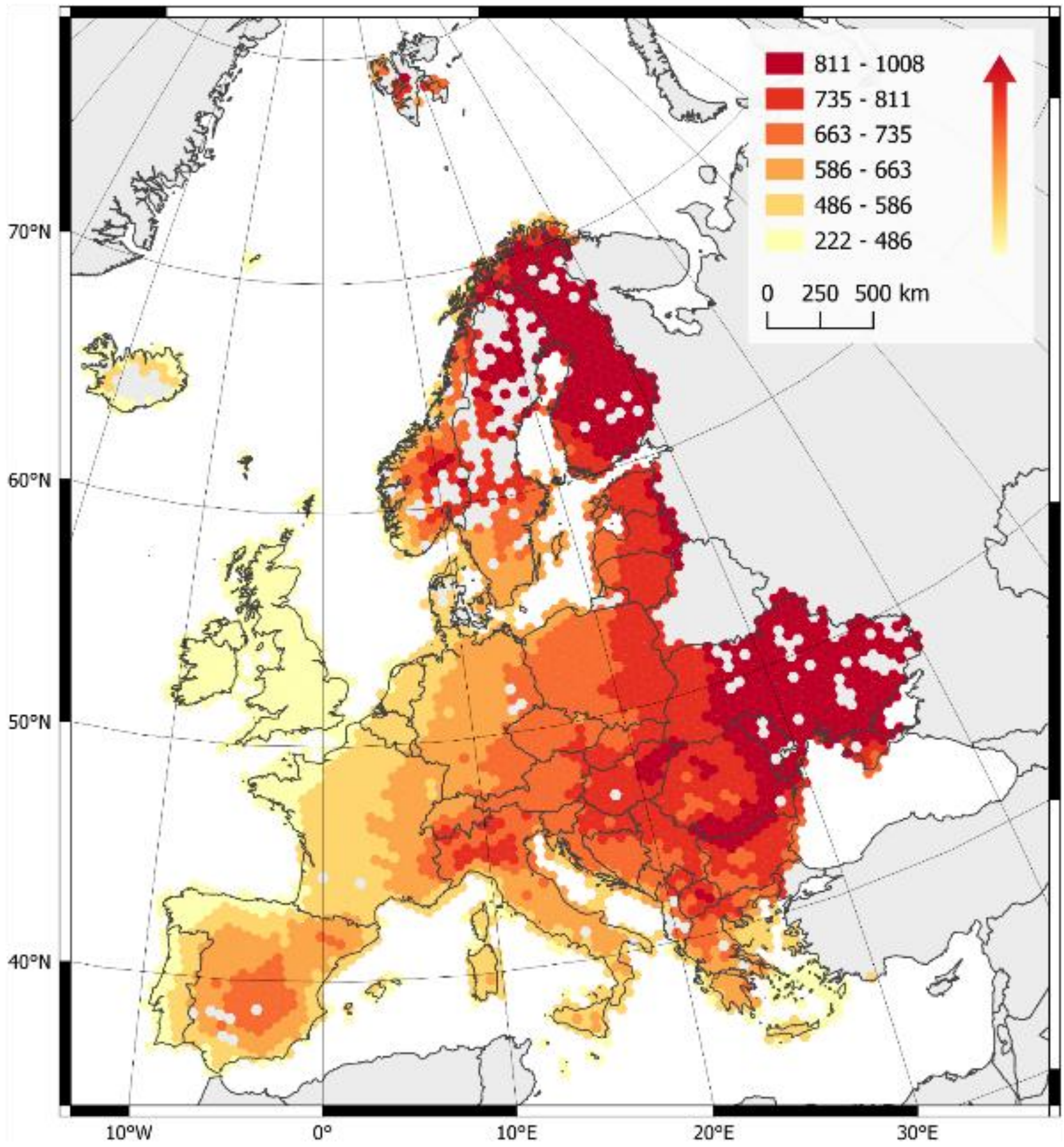

Fig. S7.2. Mean values of Temperature seasonality in °C/100 per grid cell. Grid cells are 50 km in latitudinal extent. For more information on the environmental predictors see Appendix S3.

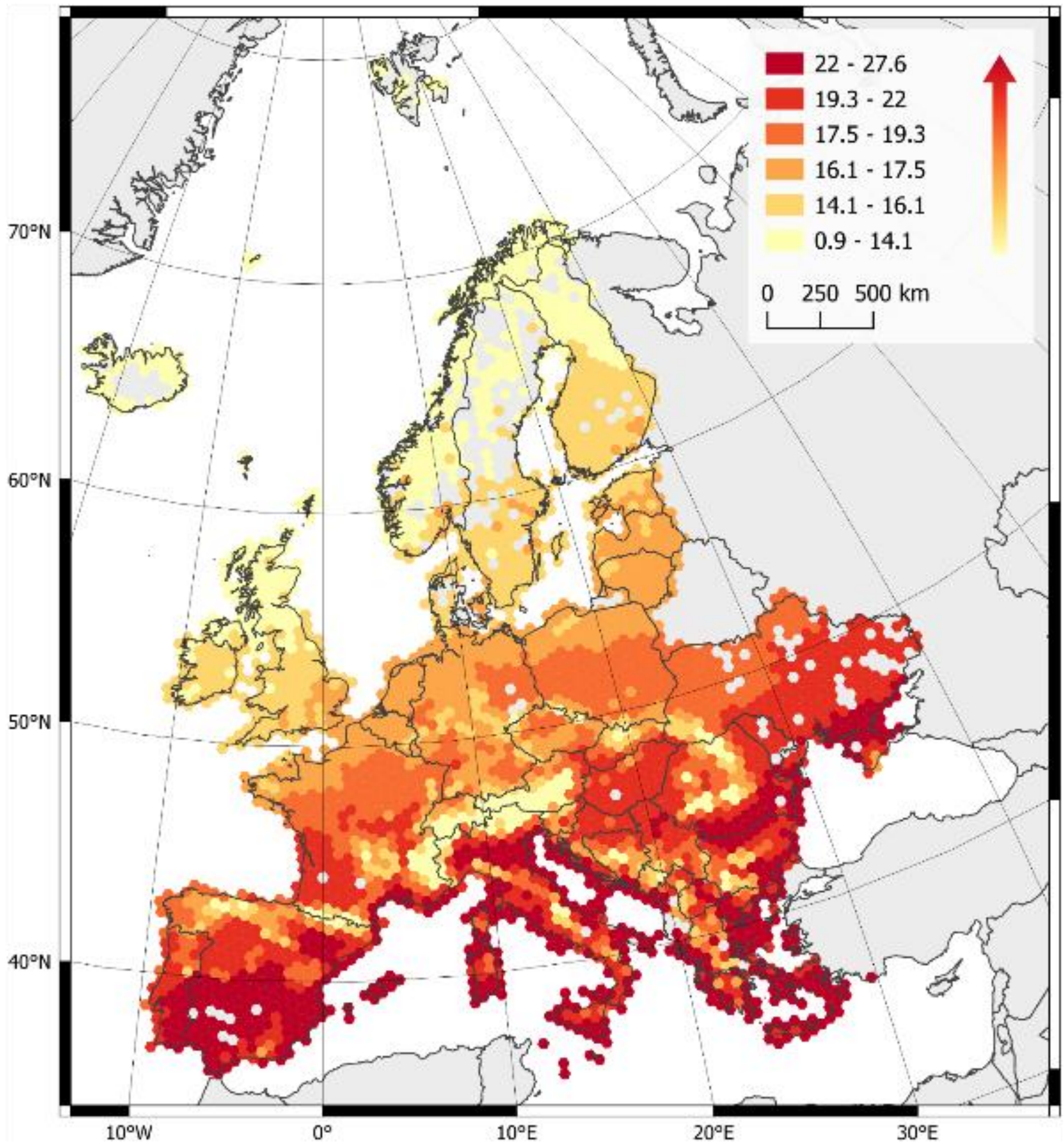

Fig. S7.3. Mean values of the Mean daily mean air temperatures of the warmest quarter in °C per grid cell. Grid cells are 50 km in latitudinal extent. For more information on the environmental predictors see Appendix S3.

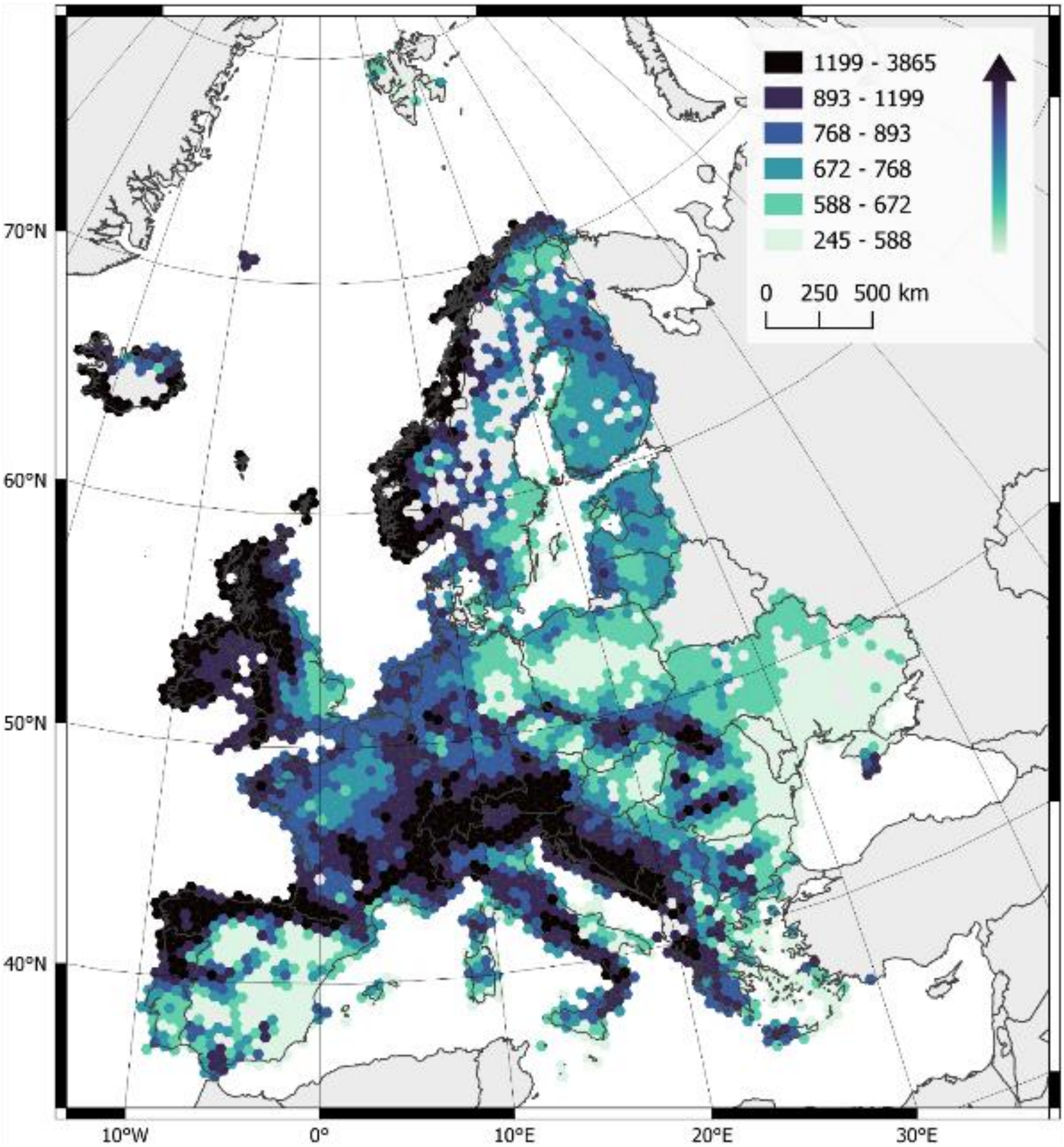

Fig. S7.4. Mean values of the Annual precipitation amount in mm/a per grid cell. Grid cells are 50 km in latitudinal extent. For more information on the environmental predictors see Appendix S3.

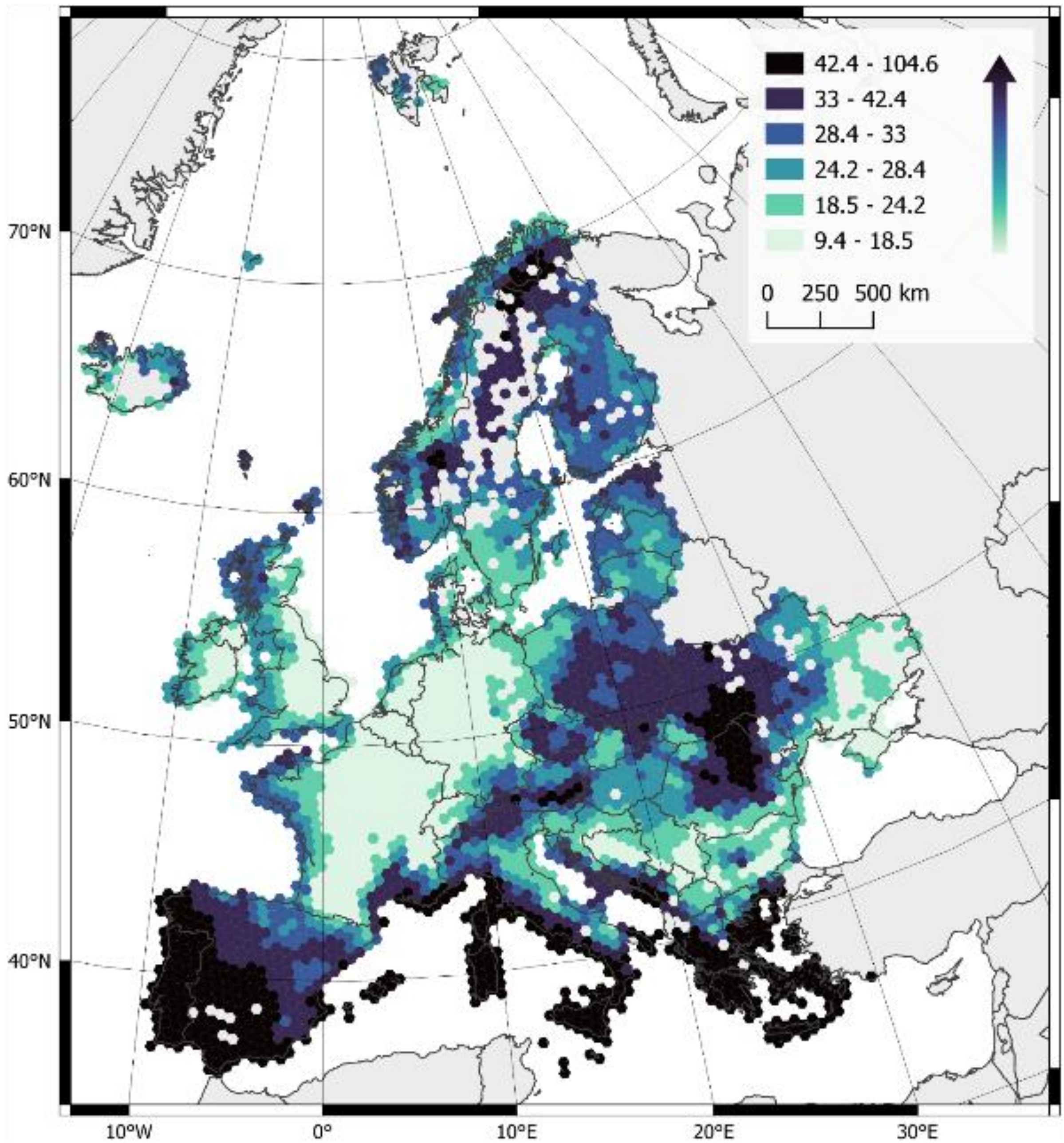

Fig. S7.5. Mean values of the Precipitation seasonality in mm per grid cell. Grid cells are 50 km in latitudinal extent. For more information on the environmental predictors see Appendix S3.

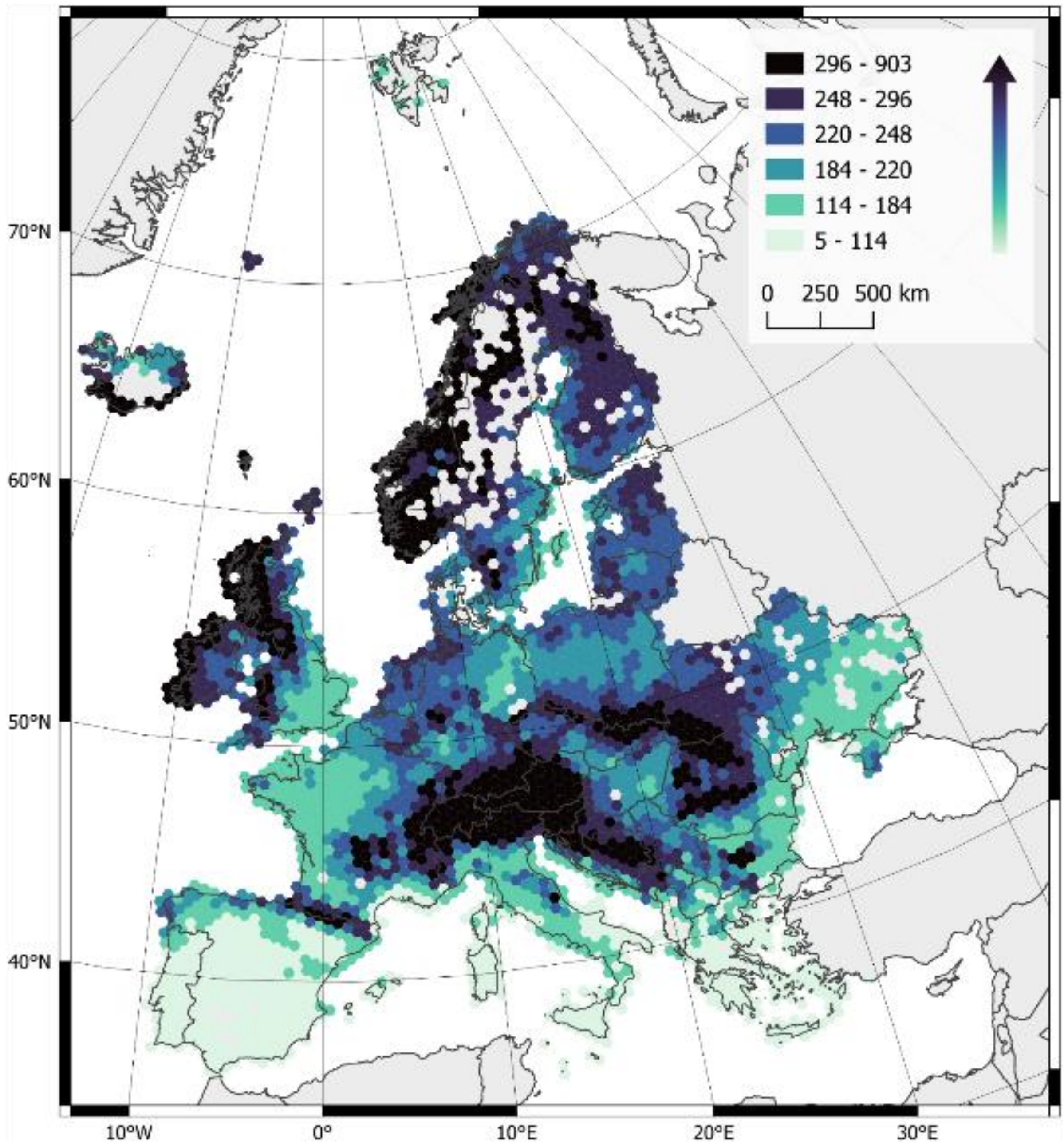

Fig. S7.6. Mean values of the Mean monthly precipitation amount of the warmest quarter mm/month per grid cell. Grid cells are 50 km in latitudinal extent. For more information on the environmental predictors see Appendix S3.

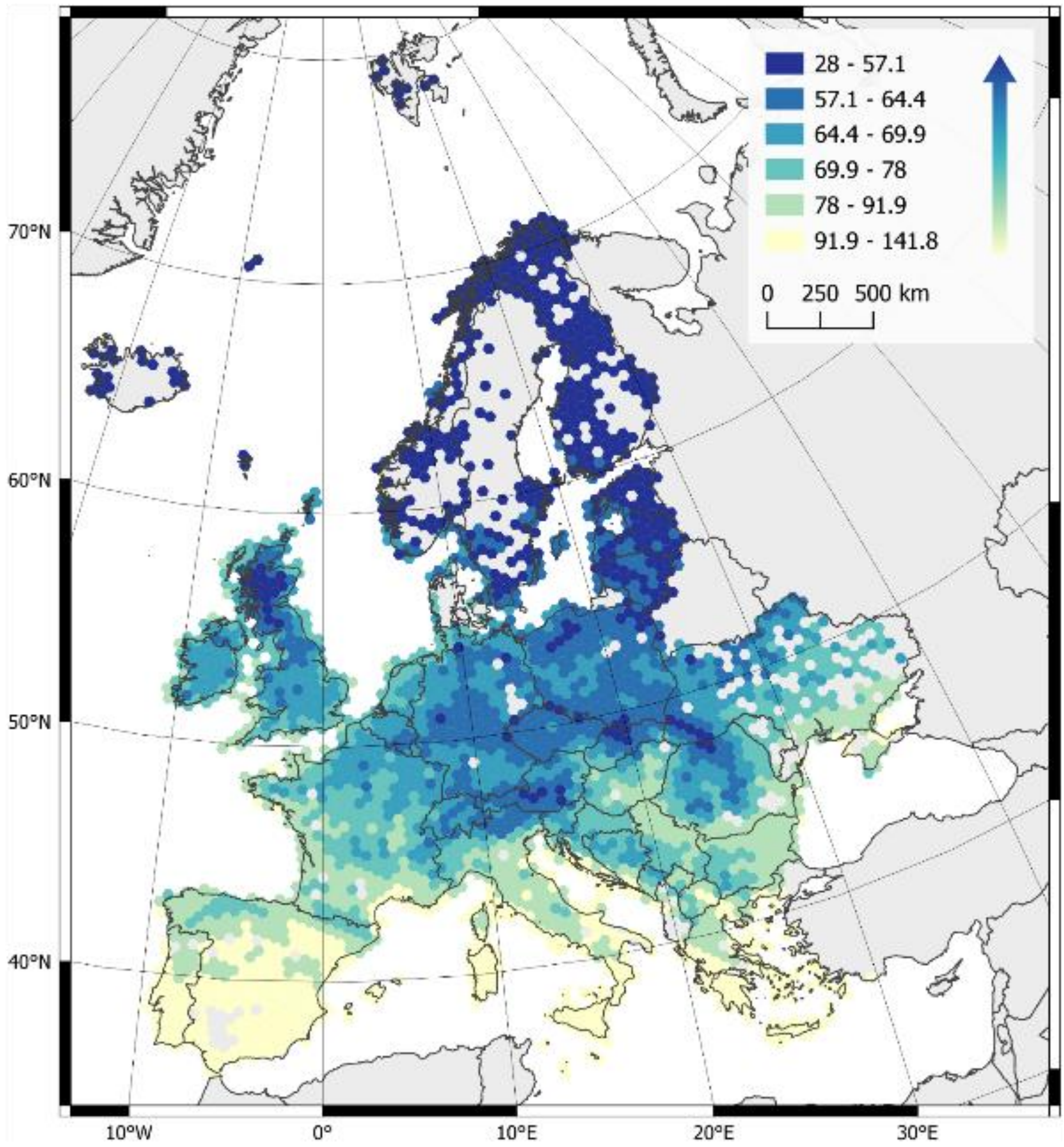

Fig. S7.7. Mean values of Mean monthly potential evapotranspiration in kg/m^2/month per grid cell. Grid cells are 50 km in latitudinal extent. For more information on the environmental predictors see Appendix S3.

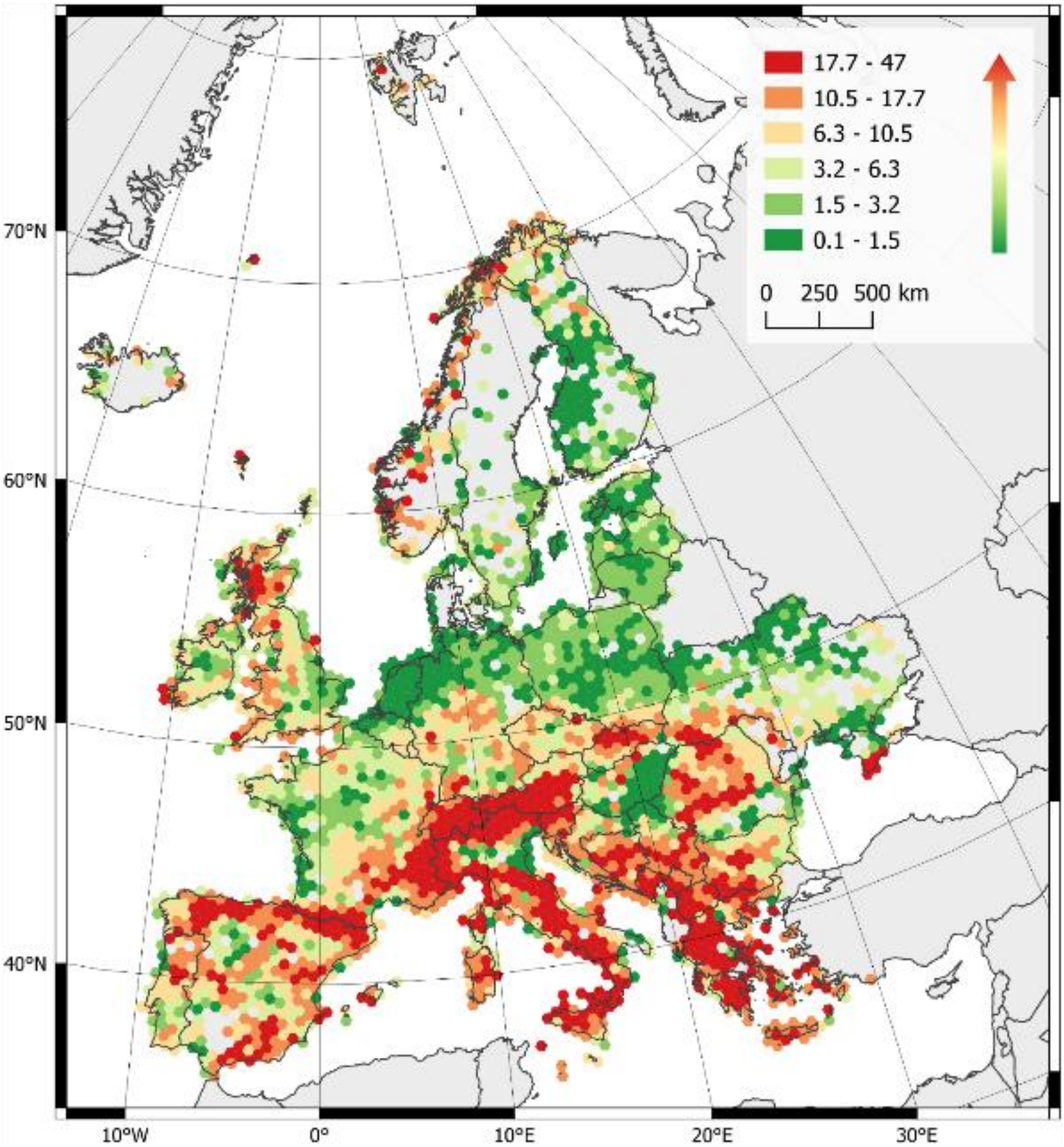

Fig. S7.8. Mean values of Terrain Ruggedness (in m) per grid cell. Grid cells are 50 km in latitudinal extent. For more information on the environmental predictors see Appendix S3.
