# Supplementary material for: Parasitic plants in Europe: ecological niches and spatial patterns
Source: Plant Biol (Stuttg). 2025 Sep 18;27(7):1285–99. doi: 10.1111/plb.70099 (PMC12631522; doi:10.1111/plb.70099)
Supplement: Supplementary file 8 — Appendix S8. Details on fitting and evaluation of the boosted regression tree analysis. [file PLB-27-1285-s001.pdf]

**APPENDIX S8.** Details on the fitting and evaluation of the boosted regression tree analysis presented in the manuscript and additional information

In Fig. S8.1 we present all the partial dependence plots from the boosted regression tree model. The results were obtained as described in the Methods section of the manuscript.

Fig. S8.2 shows the process of the selection of the optimal number of trees, which is performed in the model calculation. The standard error (SE) was calculated (i.e. 1 SE calculated as measured on the excluded folds of the cross-validation according to Elith et al., 2008) but could not be displayed in the plots as the difference to the cross-validation error line was too little to be visible in the plots. For the fitting of each tree, 50% of the full training dataset was randomly selected, with the remaining 50% used to evaluate the model's performance, promoting model robustness. A low learning rate of 0.001 was used to ensure that each tree's contribution to the model was small, and a tree complexity of 5 was chosen to capture interactions up to the fifth order, achieving high predictive power while maintaining reasonable computation times. The optimal number of regression trees was identified through a 10-fold cross-validation procedure.

To test for spatial autocorrelation using Moran's *I*, we randomly selected 200 plots and calculated the spatial correlogram for all plots located within 2° (~225 km) of the target plot selected from a random subset of 20% of plots from our dataset. The resulting correlograms were then summarised using loess fitting.

The evaluation statistics in Tab. S8.1 are extracted from the 'gbm' object produced with the *gbm.step* in the *'dismo'* package following Elith et al. 2008 as described in the Methods section of the manuscript and extracted using the *ggPerformance* function in the *'ggBRT'* package (Jouffray et al., 2019). Statistics are fold-based, meaning they are calculated at the identified optimal number of trees, which is calculated on the mean change in predictive deviance of the model over all folds (Fig. S8.2).

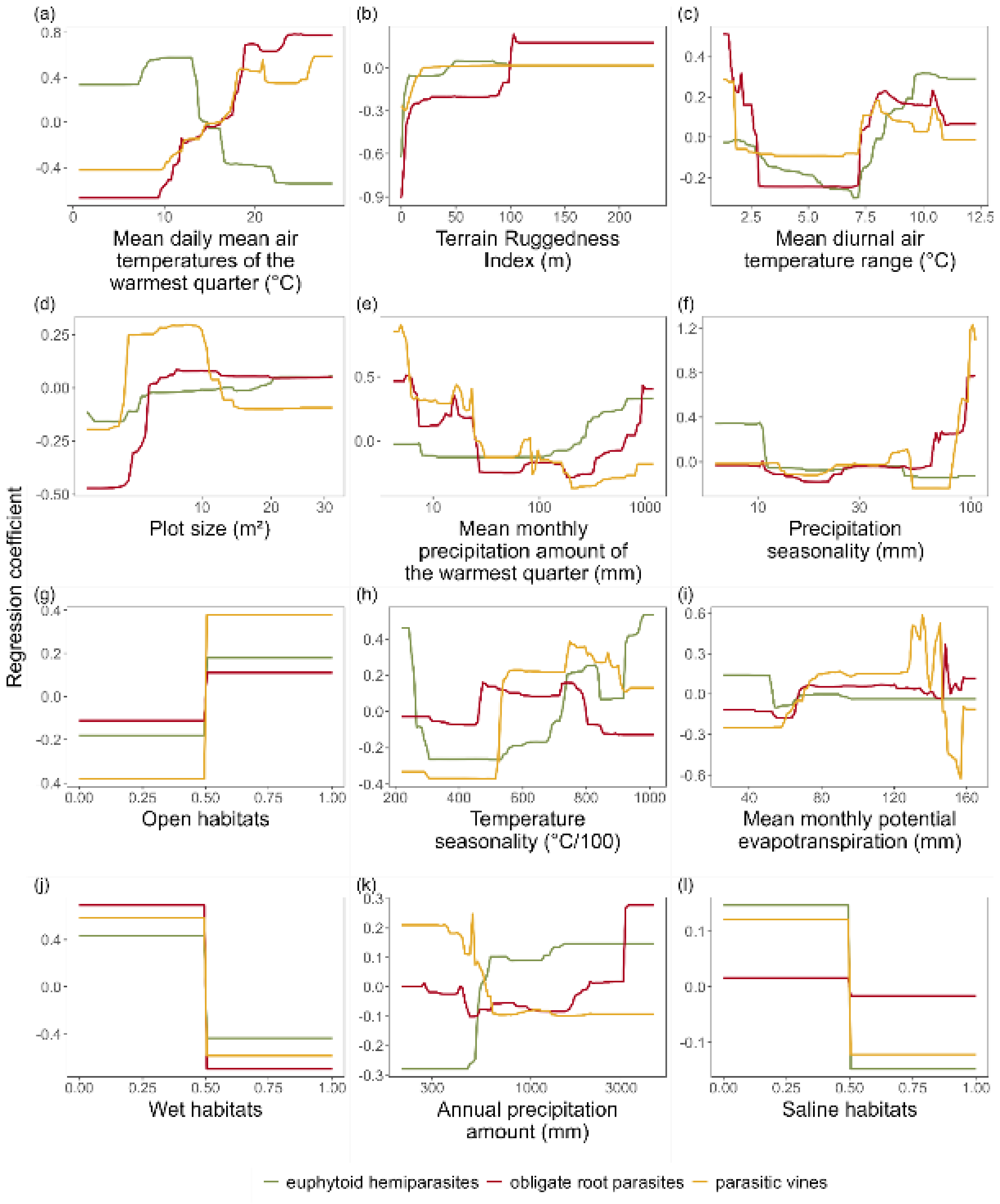


**Fig. S8.1.** The partial dependence plots from the boosted regression tree models (BRT) show the effect of environmental variables (a-l) on the abundance of parasitic functional types in Europe. The lines show the curves of the fitted functions. Values of precipitation (e, f, j, l) are shown on log-scaled axes, (d) on a sqrt-transformed axis.

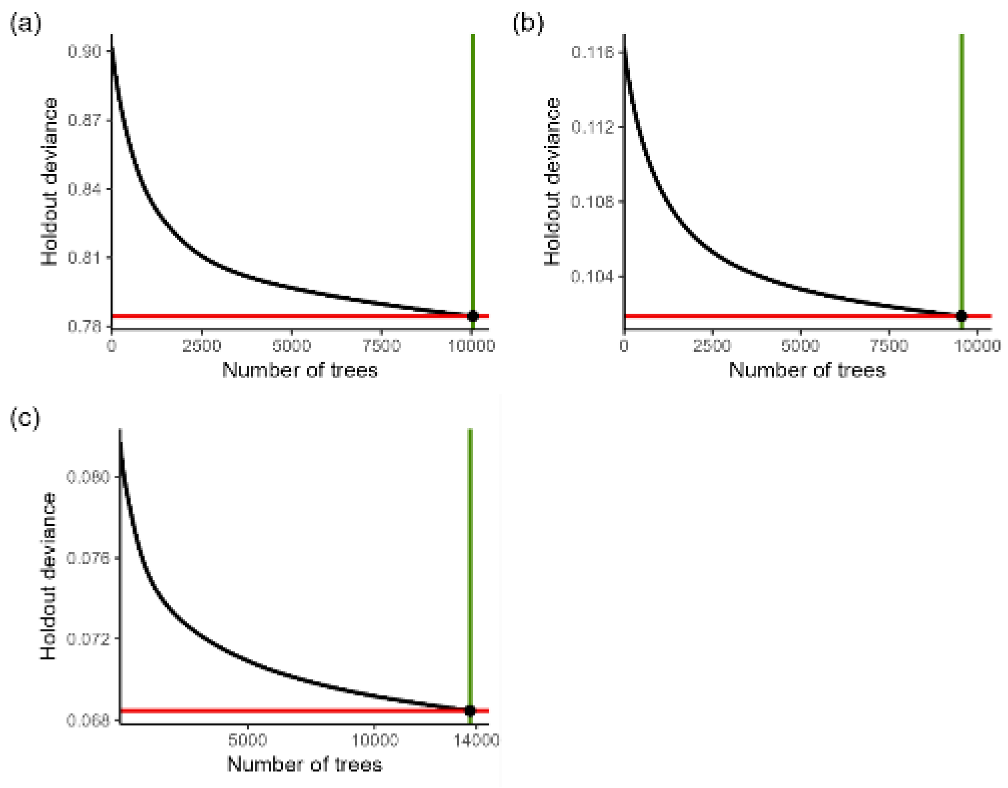


**Fig. S8.2.** Selections of the optimal number of regression trees in the boosted regression tree models for each functional type: a) euphytoid hemiparasites; b) obligate root parasites; c) parasitic vines. The black line shows the decrease in the mean of the cross-validation error with the increased number of trees added. The red line shows the minimum of the mean of the cross-validation error, and the green line is the number of trees at which it occurs (i.e., the number of trees used to build the final model).

**Table S8.1.** Evaluation statistics and plot numbers of the boosted regression tree models for each functional type.

| Model | No. of | | Deviance | | | | CV Correlation | | CV AUC | |
|---|---|---|---|---|---|---|---|---|---|---|
| | plots | trees | Mean Total | Mean Residual | SE | Percent explained ($D^2$) | Cor | SE | AUC | SE |
| Euph. hemiparasites | 565063 | 10050 | 0.902 | 0.783 | 0.001 | 13.142 | 0.355 | 0.001 | 0.756 | 0.001 |
| Obl. root parasites | 565063 | 9550 | 0.116 | 0.100 | <0.001 | 13.746 | 0.164 | 0.005 | 0.807 | 0.004 |
| Parasitic vines | 565063 | 13750 | 0.082 | 0.067 | <0.001 | 16.179 | 0.183 | 0.007 | 0.854 | 0.003 |

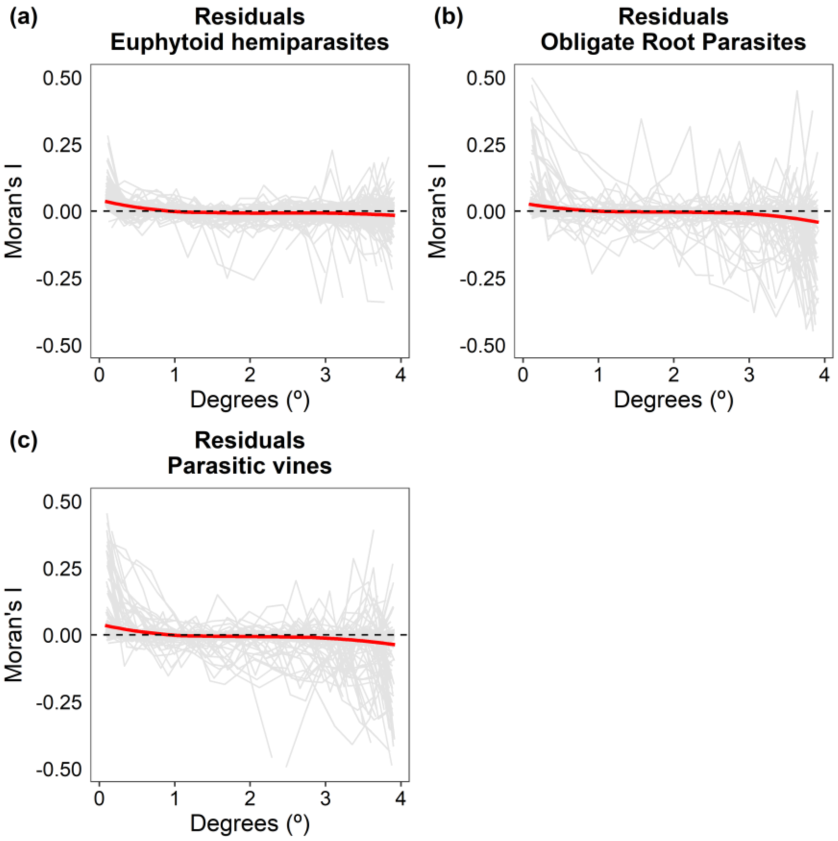


**Fig. S8.3.** Spatial correlograms of model residuals for each parasitic plant functional type (response variable) from the boosted regression tree model using environmental variables and habitat information on the fine level (dataset c). We calculated Moran's I statistic for plots within a 2º radius (~225 km) of 200 randomly selected plots, randomly distributed across the sampled regions, using a random subset of 20% of plots from our dataset. Red lines summarise overall average estimates across all repetitions using loess regression.

REFERENCES

Jouffray, J.-B., Wedding, L. M., Norström, A. V., Donovan, M. K., Williams, G. J., Crowder, L. B., Erickson, A. L., Friedlander, A. M., Graham, N. A. J., Gove, J. M., Kappel, C. V., Kittinger, J. N., Lecky, J., Oleson, K. L. L., Selkoe, K. A., White, C., Williams, I. D., & Nyström, M. (2019). Parsing human and biophysical drivers of coral reef regimes. *Proceedings of the Royal Society B: Biological Sciences*, *286*(1896), 20182544. https://doi.org/10.1098/rspb.2018.2544
