# Supplementary material for: Parasitic plants in Europe: ecological niches and spatial patterns
Source: Plant Biol (Stuttg). 2025 Sep 18;27(7):1285–99. doi: 10.1111/plb.70099 (PMC12631522; doi:10.1111/plb.70099)
Supplement: Supplementary file 9 — Appendix S9. Additional information for the modified permutation test for EIVE values. [file PLB-27-1285-s008.pdf]

APPENDIX S9. Additional information for the modified permutation test for EIVE values

Results of the modified permutation test procedure (Zeleny & Schaffers, 2012), testing the difference between Environmental Indicator Values for Europe (EIVE values) for plots where parasitic plants are present and absent. The red dashed line displays the actual difference, while the grey histogram represents the results of the 1000 permutations of species EIVE values.

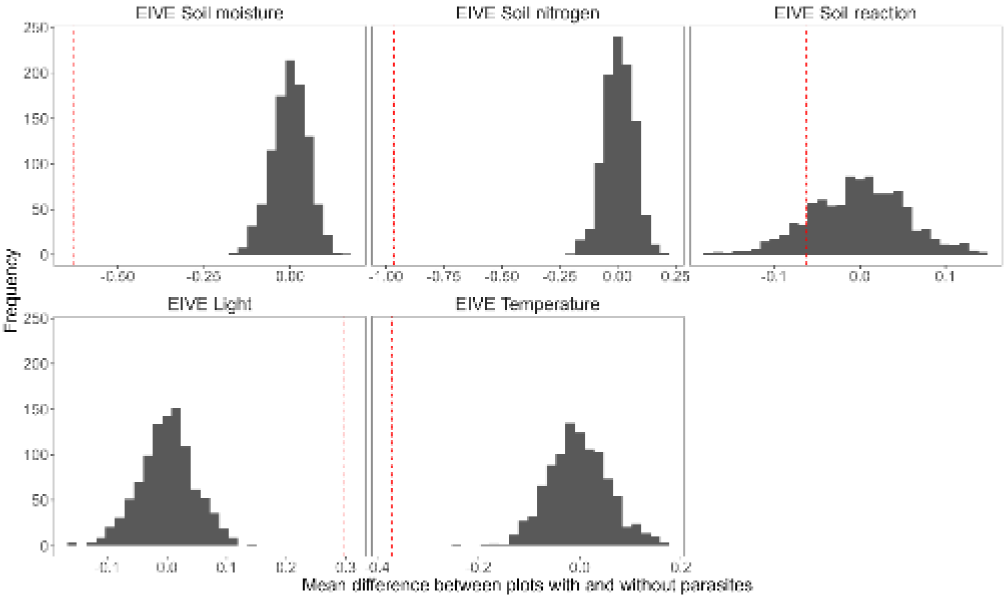

Fig. S9.1. Distribution of the differences between plots with and without euphytoid hemiparasites, generated by calculating EIVE plot values after permutation of species values. We used 1000 permutations. The red dashed line shows the actual difference between plots with and without euphytoid hemiparasites.

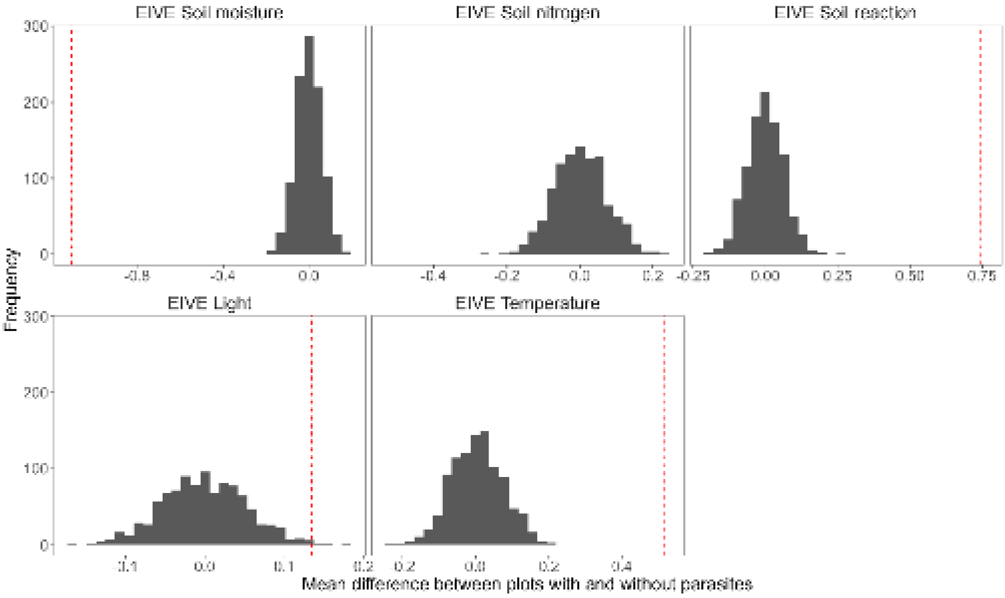

Fig. S9.2. Distribution of the differences between plots with and without obligate root parasites, generated by calculating EIVE plot values after permutation of species values. We used 1000 permutations. The red dashed line shows the actual difference between plots with and without obligate root parasites.

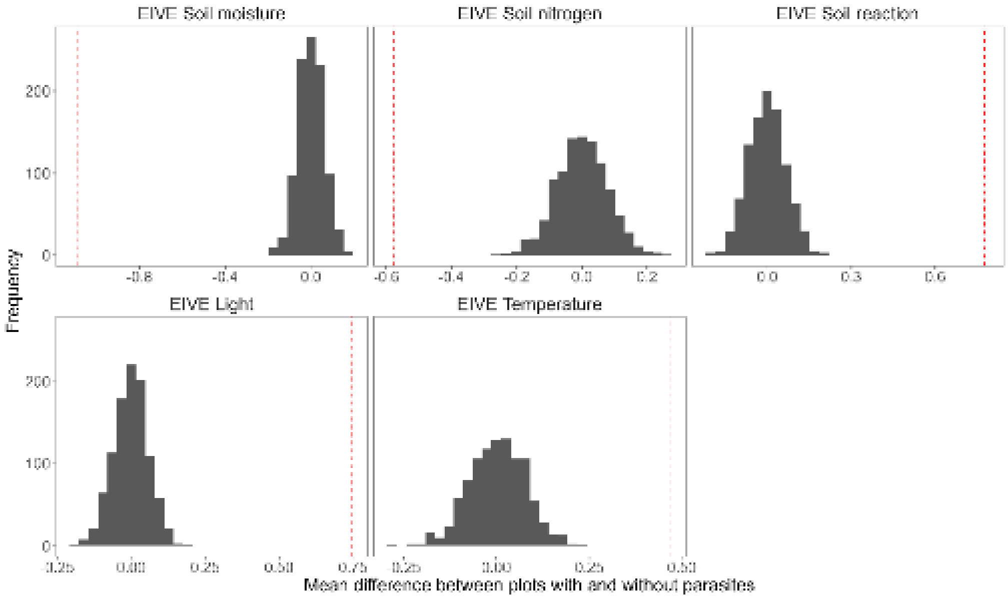

Fig. S9.3. Distribution of the differences between plots with and without parasitic vines, generated by calculating EIVE plot values after permutation of species values. We used 1000 permutations. The red dashed line shows the actual difference between plots with and without parasitic vines.
