# Supplementary material for: Parasitic plants in Europe: ecological niches and spatial patterns
Source: Plant Biol (Stuttg). 2025 Sep 18;27(7):1285–99. doi: 10.1111/plb.70099 (PMC12631522; doi:10.1111/plb.70099)
Supplement: Supplementary file 10 — Appendix S10. Additional results for analysis of niches along all environmental variables. [file PLB-27-1285-s004.pdf]

APPENDIX S10. Additional results for the analysis of niches along all environmental variables

Niches were calculated as described in the Methods of the manuscript. Here we present the results for all environmental variables. Diverging from the described methods, results are not presented according to phylogeny but sorted alphabetically for all species having at least 50 occurrences in the final dataset.

Fig. S10.1. Mean diurnal air temperature range (°C)
Fig. S10.2. Temperature seasonality (°C/100)
Fig. S10.3. Mean daily mean air temperatures of the warmest quarter (°C)
Fig. S10.4. Annual precipitation amount (mm)
Fig. S10.5. Precipitation seasonality (mm)
Fig. S10.6. Mean monthly precipitation amount of the warmest quarter (mm)
Fig. S10.7. Mean monthly potential evapotranspiration (kg/m^2/month)
Fig. S10.8. Terrain Ruggedness Index (m)
Fig. S10.9. Wet habitats
Fig. S10.10. Open habitats
Fig. S10.11. Saline habitats
Fig. S10.12. EIVE Soil moisture
Fig. S10.13. EIVE Light
Fig. S10.14. EIVE Soil nitrogen
Fig. S10.15. EIVE Soil reaction
Fig. S10.16. Temperature

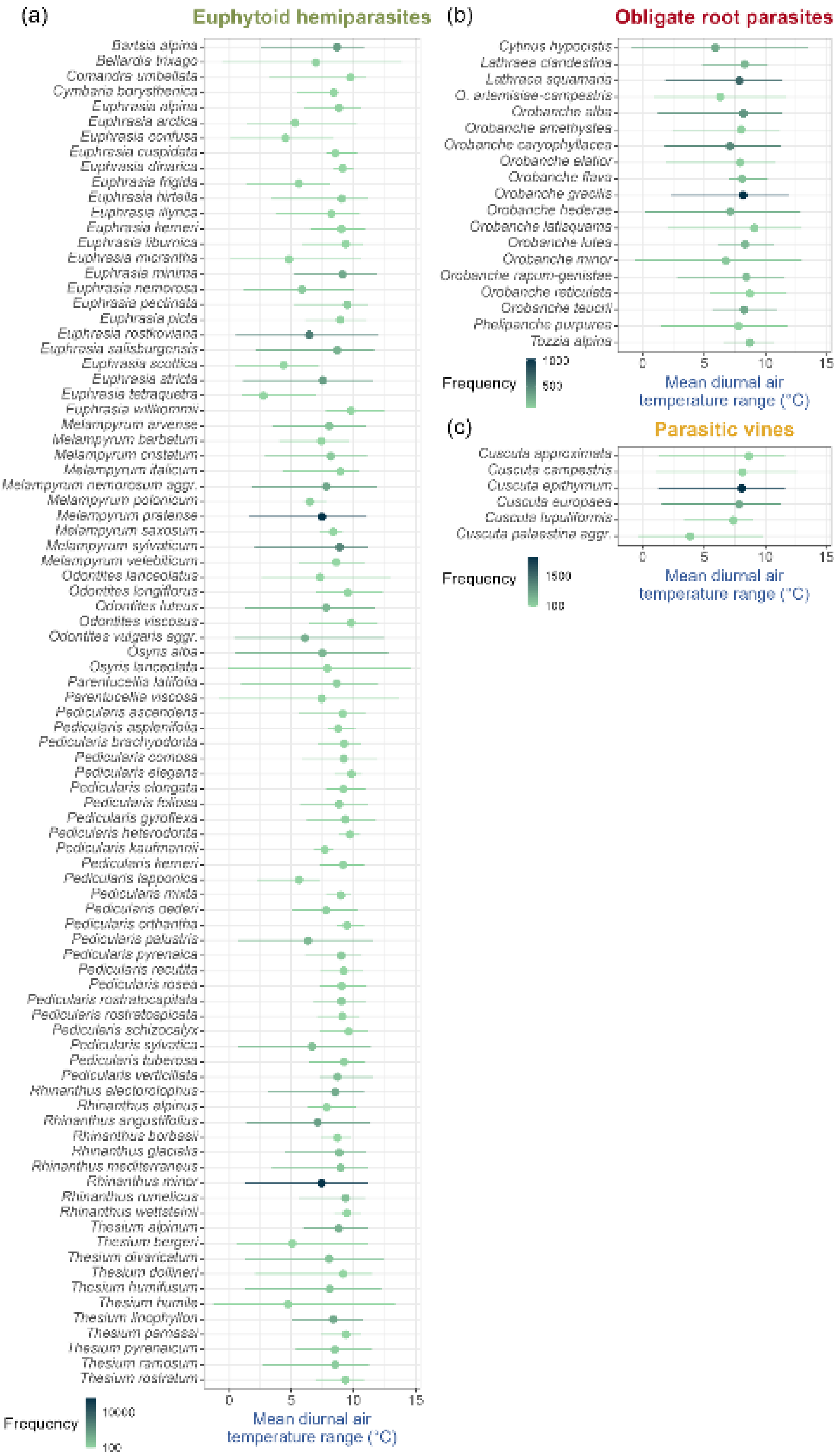

Fig. S10.1. Ranges (lines) and optima (full points) of parasitic plant species along the Mean diurnal air temperature range (°C) for a) euphytoid hemiparasites, b) obligate root parasites, and c) parasitic vines. The ranges were defined as intervals covering 95% of cover-weighted presences of given species while the optima were defined as cover-weighted average.

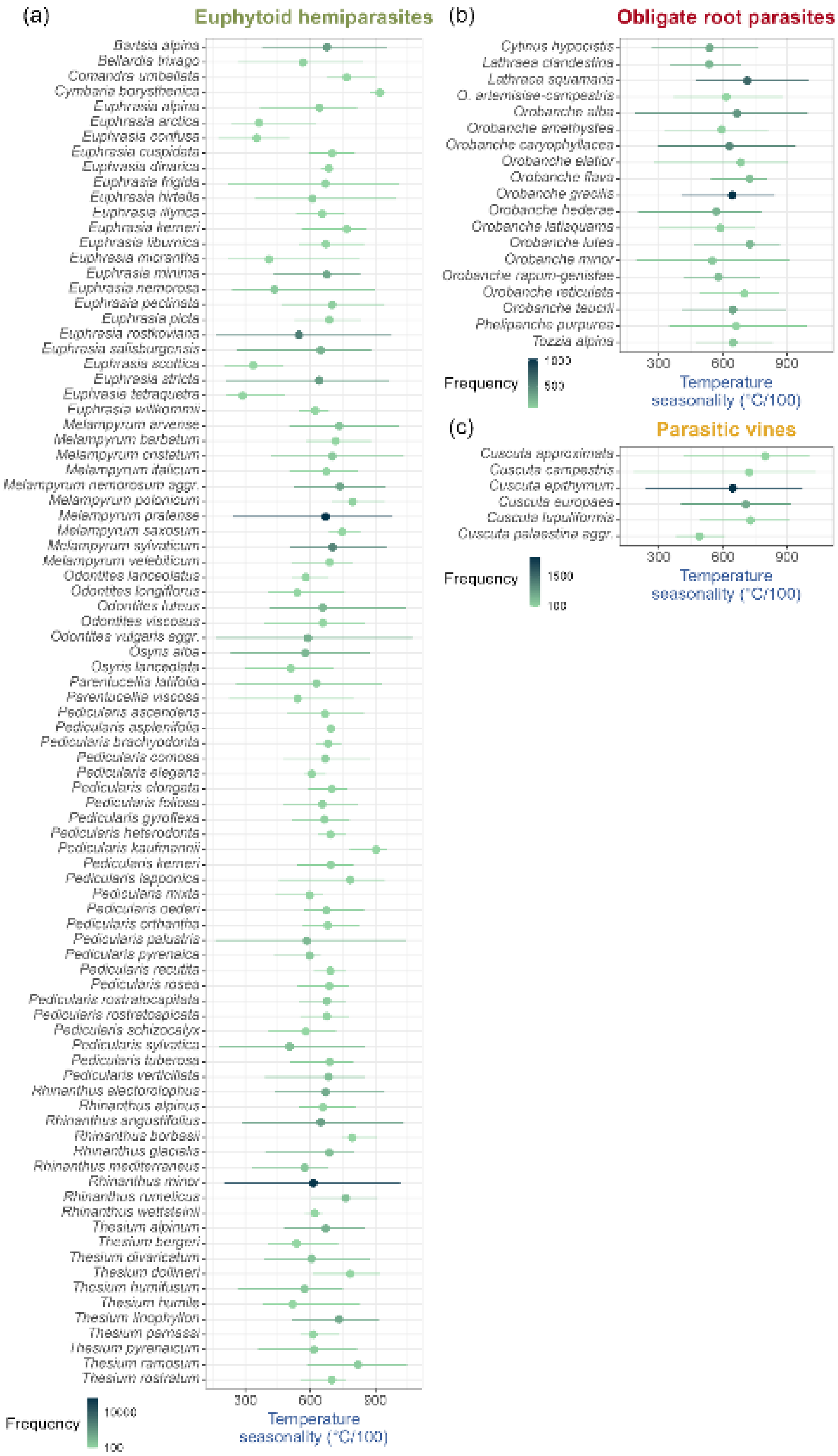

Fig. S10.2. Ranges (lines) and optima (full points) of parasitic plant species along the Temperature seasonality (°C/100) for a) euphytoid hemiparasites, b) obligate root parasites, and c) parasitic vines. The ranges were defined as intervals covering 95% of cover-weighted presences of given species while the optima were defined as cover-weighted average.

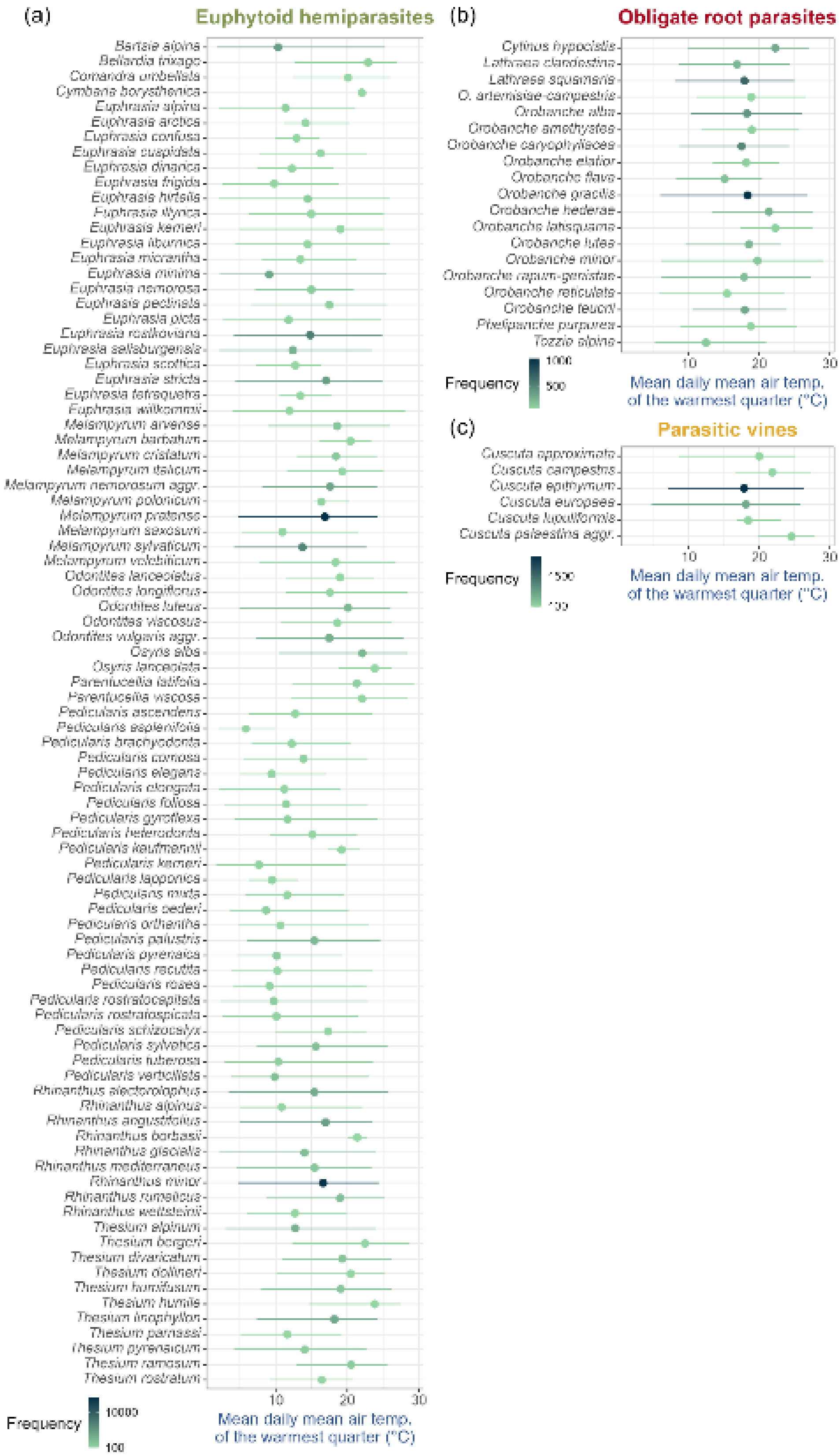

Fig. S10.3. Ranges (lines) and optima (full points) of parasitic plant species along the Mean daily mean air temperatures of the warmest quarter (°C) for a) euphytoid hemiparasites, b) obligate root parasites, and c) parasitic vines. The ranges were defined as intervals covering 95% of cover-weighted presences of given species while the optima were defined as cover-weighted average.

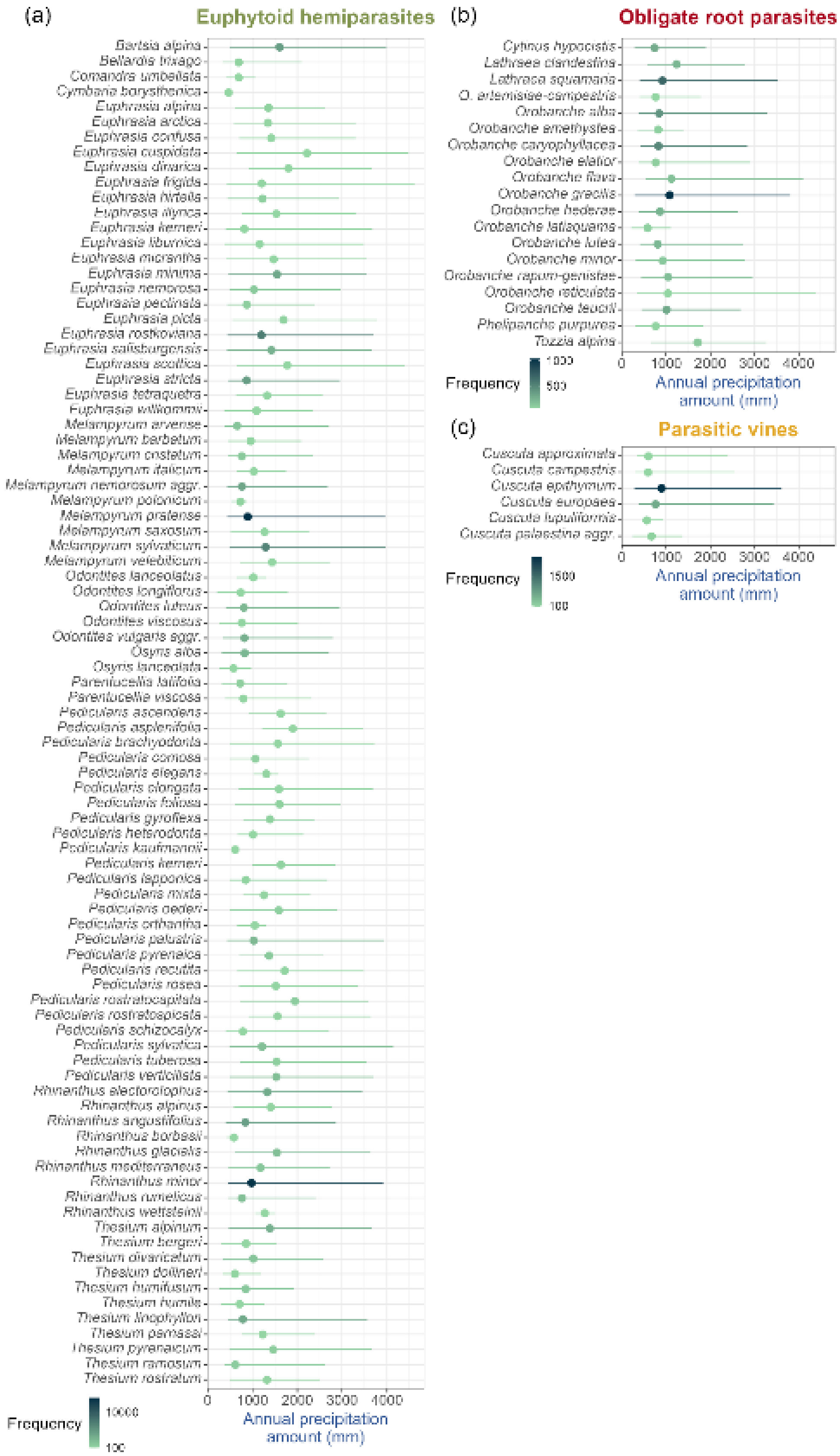

Fig. S10.4. Ranges (lines) and optima (full points) of parasitic plant species along the Annual Annual precipitation amount (mm) for a) euphytoid hemiparasites, b) obligate root parasites, and c) parasitic vines. The ranges were defined as intervals covering 95% of cover-weighted presences of given species while the optima were defined as cover-weighted average.

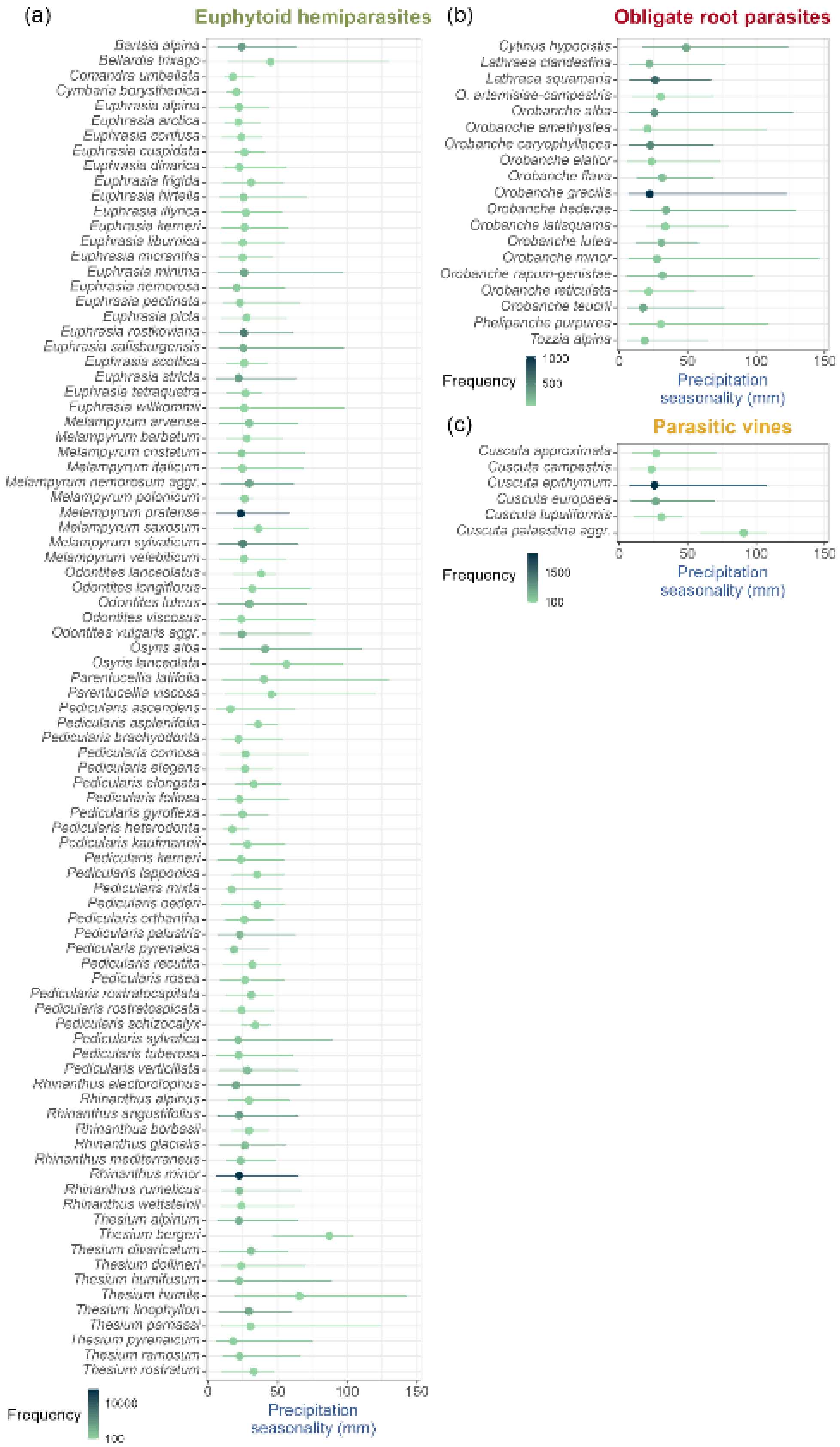

Fig. S10.5. Ranges (lines) and optima (full points) of parasitic plant species along Precipitation seasonality (mm) for a) euphytoid hemiparasites, b) obligate root parasites, and c) parasitic vines. The ranges were defined as intervals covering 95% of cover-weighted presences of given species while the optima were defined as cover-weighted average.

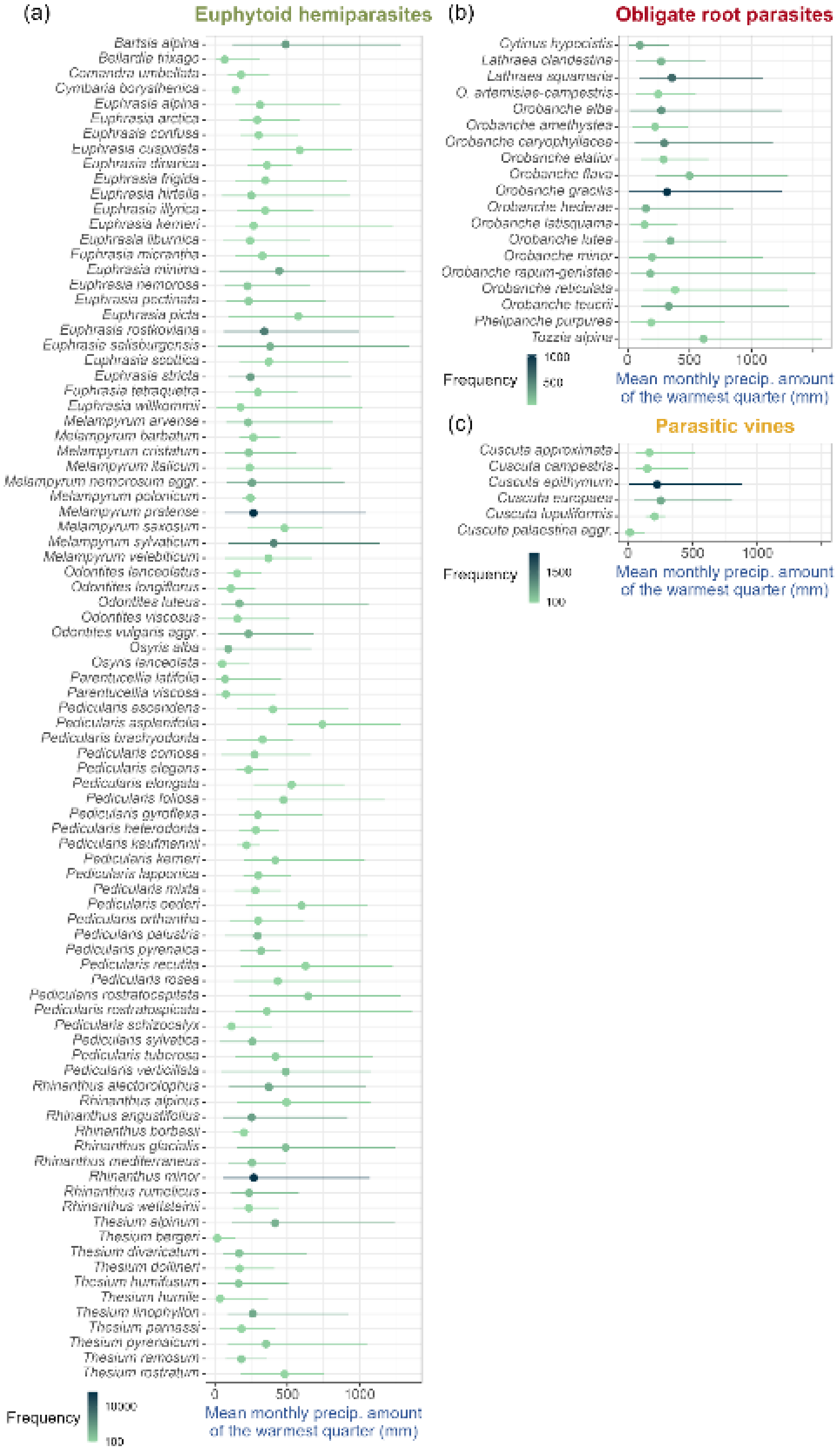

Fig. S10.6. Ranges (lines) and optima (full points) of parasitic plant species along the Mean monthly precipitation amount of the warmest quarter (mm) for a) euphytoid hemiparasites, b) obligate root parasites, and c) parasitic vines. The ranges were defined as intervals covering 95% of cover-weighted presences of given species while the optima were defined as cover-weighted average.

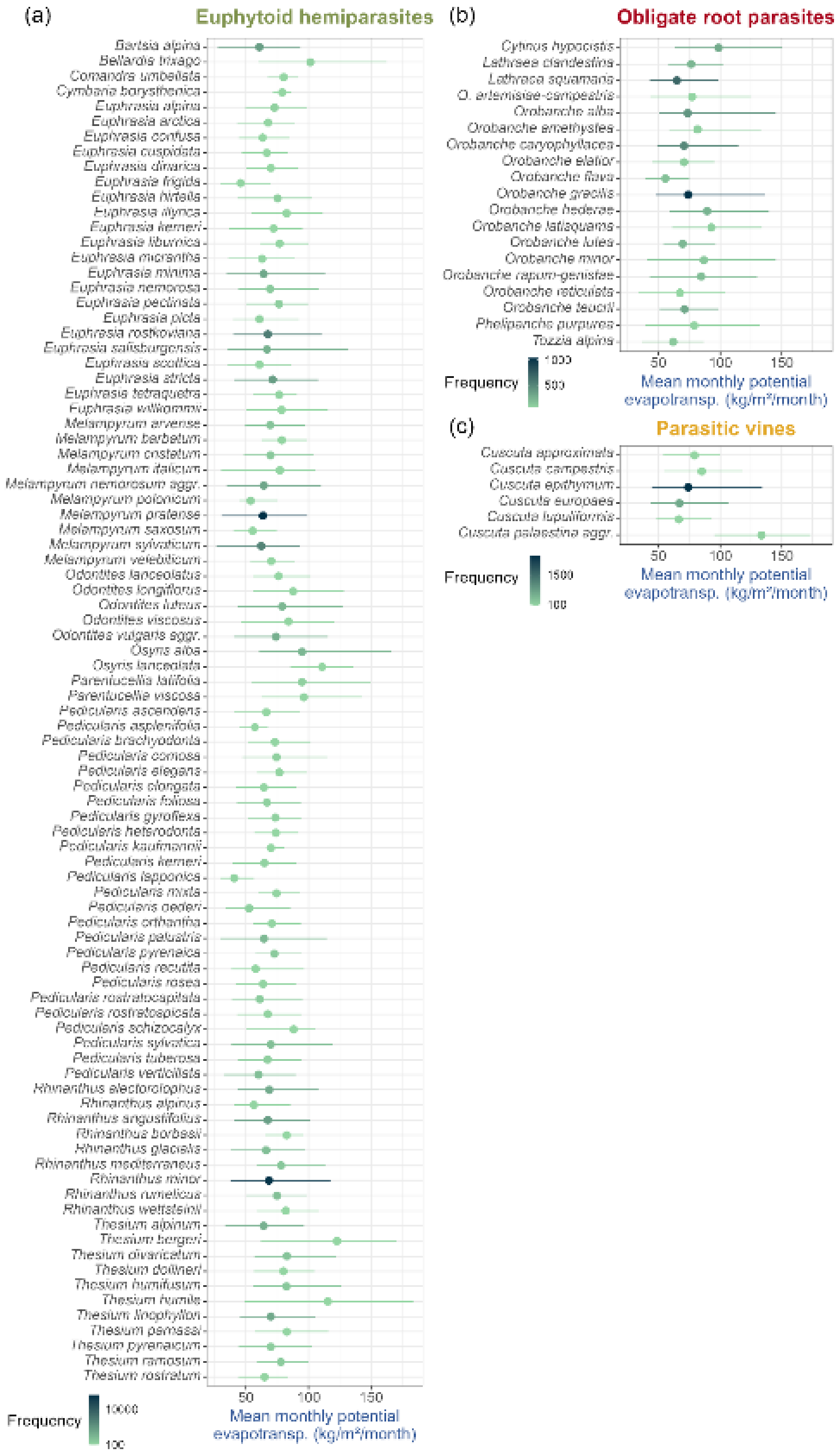

Fig. S10.7. Ranges (lines) and optima (full points) of parasitic plant species along the Mean monthly potential evapotranspiration (kg/m^2/month) for a) euphytoid hemiparasites, b) obligate root parasites, and c) parasitic vines. The ranges were defined as intervals covering 95% of cover-weighted presences of given species while the optima were defined as cover-weighted average.

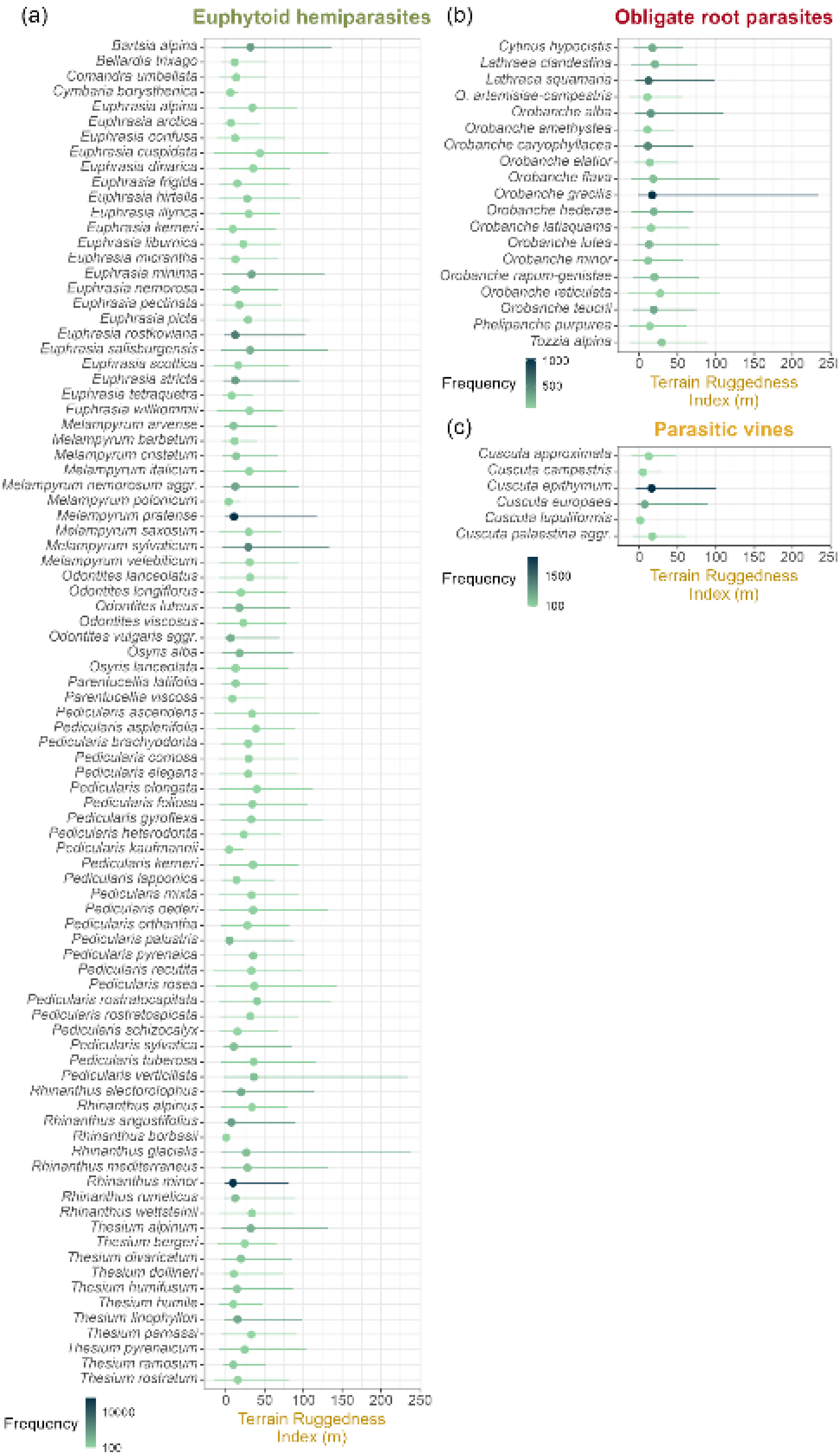

Fig. S10.8. Ranges (lines) and optima (full points) of parasitic plant species along the Terrain Ruggedness Index (m) for a) euphytoid hemiparasites, b) obligate root parasites, and c) parasitic vines. The ranges were defined as intervals covering 95% of cover-weighted presences of given species while the optima were defined as cover-weighted average.

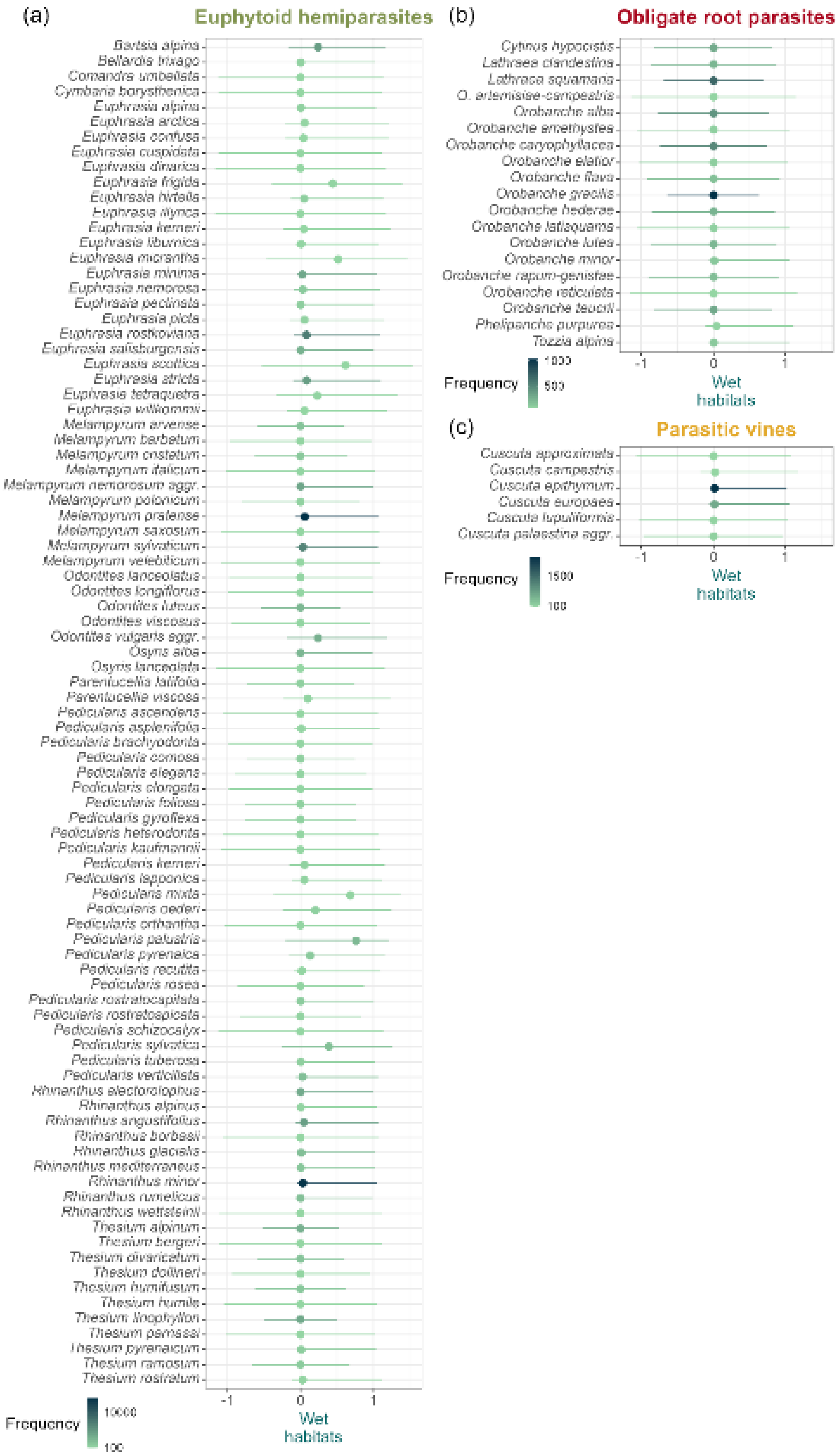

Fig. S10.9. Ranges (lines) and optima (full points) of parasitic plant species along Wet habitats for a) euphytoid hemiparasites, b) obligate root parasites, and c) parasitic vines. The ranges were defined as intervals covering 95% of cover-weighted presences of given species while the optima were defined as cover-weighted average.

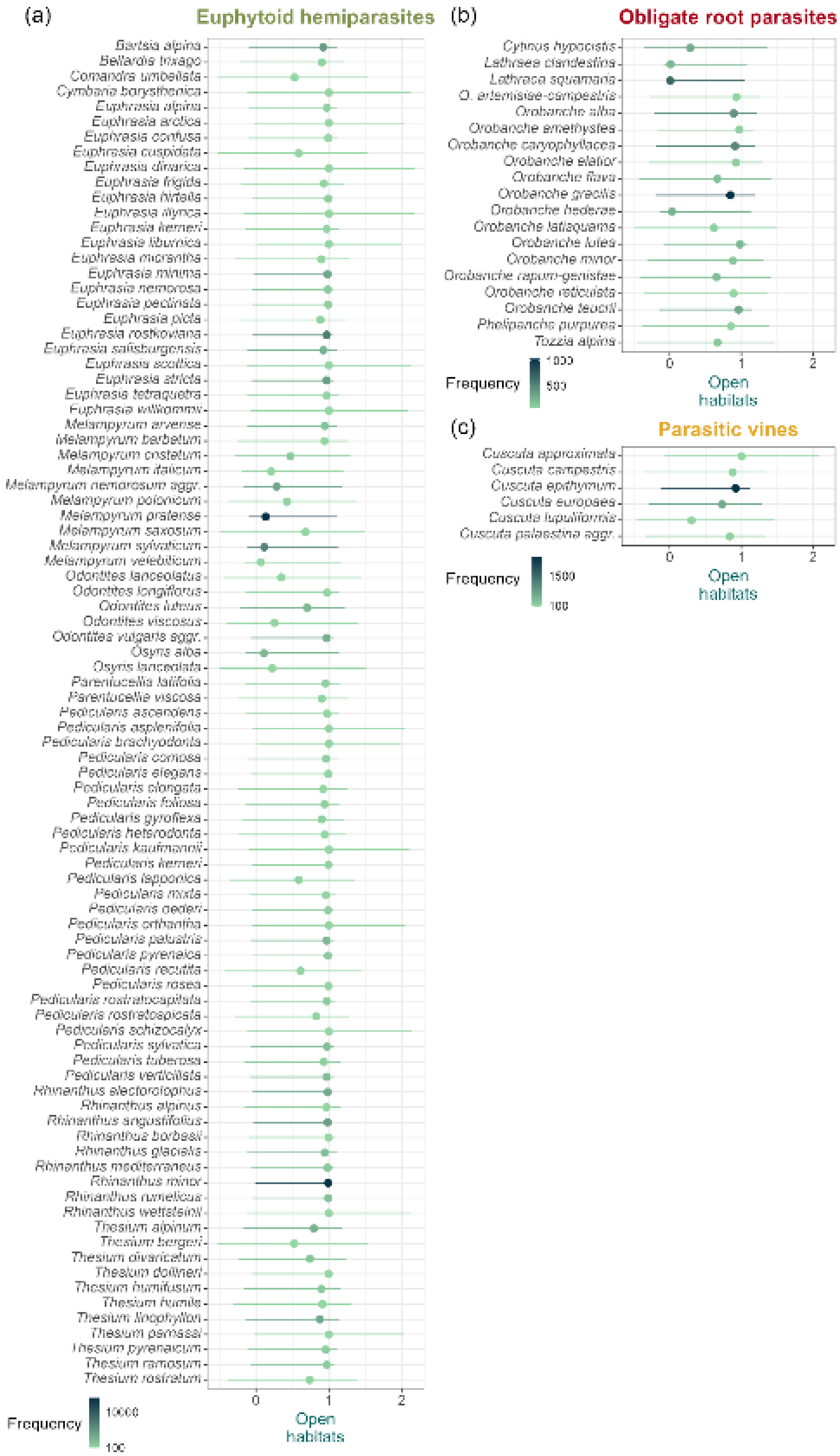

Fig. S7.10. Ranges (lines) and optima (full points) of parasitic plant species along Open habitats for a) euphytoid hemiparasites, b) obligate root parasites, and c) parasitic vines. The ranges were defined as intervals covering 95% of cover-weighted presences of given species while the optima were defined as cover-weighted average.

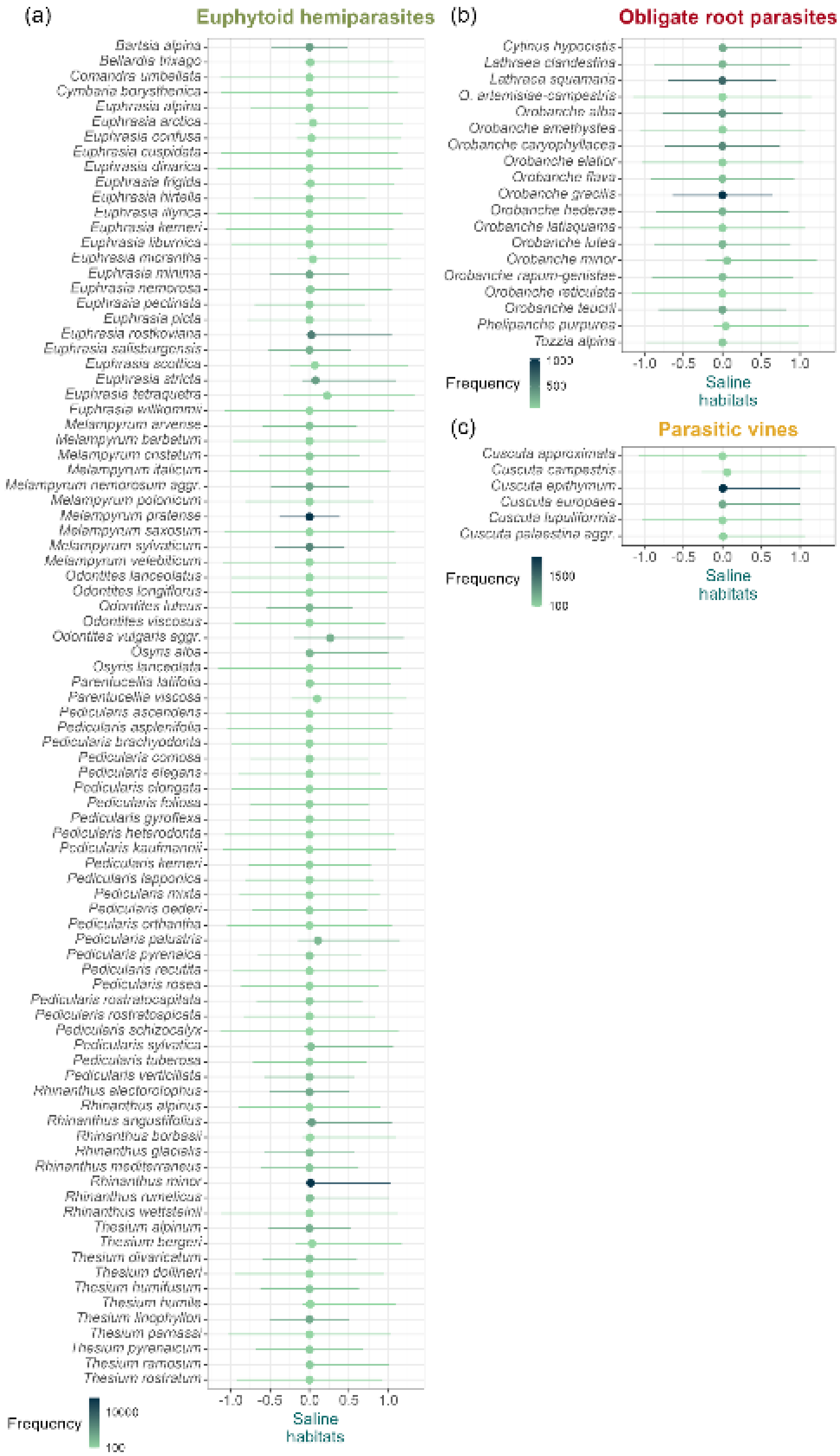

Fig. S10.11. Ranges (lines) and optima (full points) of parasitic plant species along Saline Habitats for a) euphytoid hemiparasites, b) obligate root parasites, and c) parasitic vines. The ranges were defined as intervals covering 95% of cover-weighted presences of given species while the optima were defined as cover-weighted average.

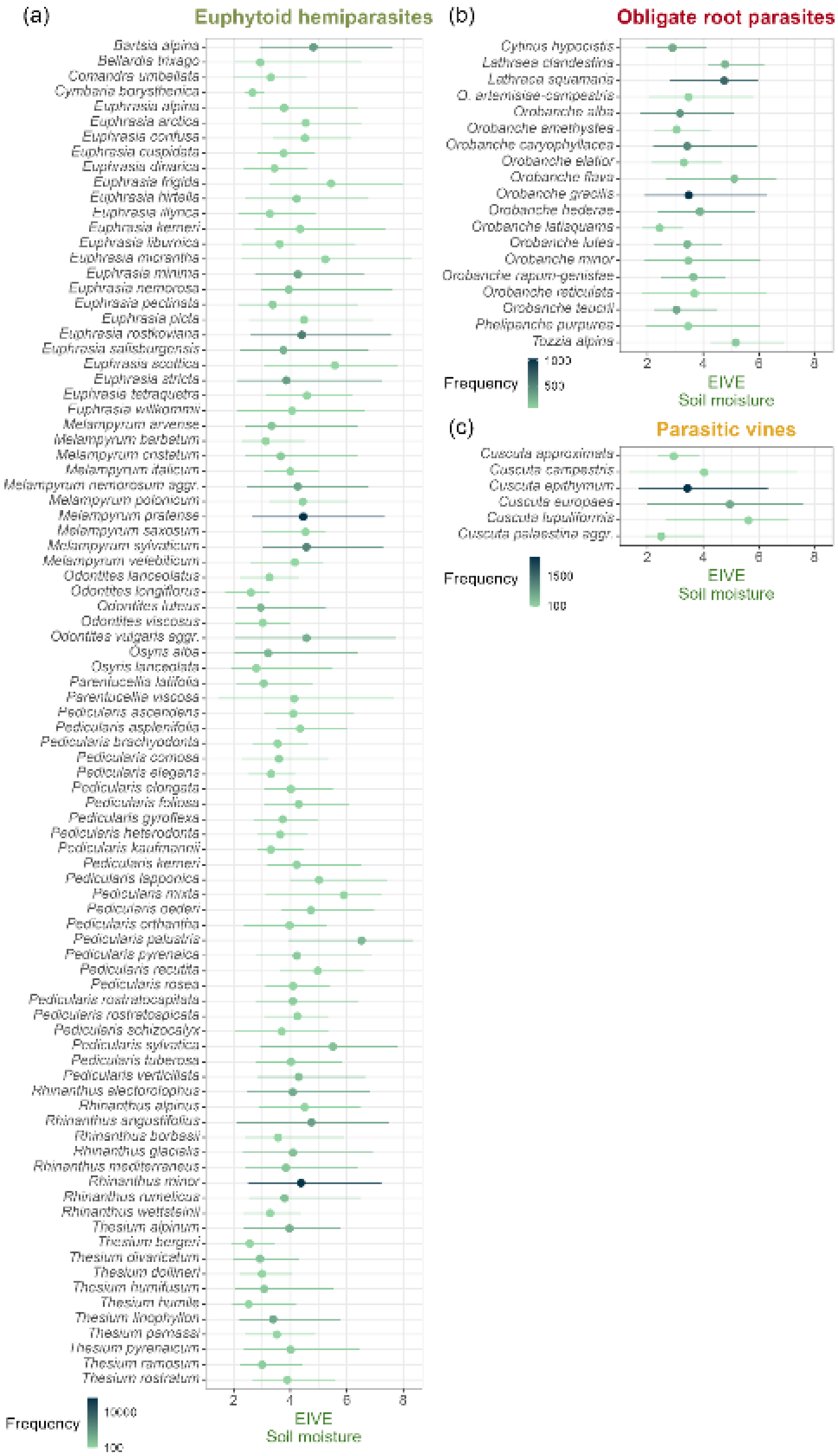

Fig. S10.12. Ranges (lines) and optima (full points) of parasitic plant species along the EIVE Soil moisture for a) euphytoid hemiparasites, b) obligate root parasites, and c) parasitic vines. The ranges were defined as intervals covering 95% of cover-weighted presences of given species while the optima were defined as cover-weighted average.

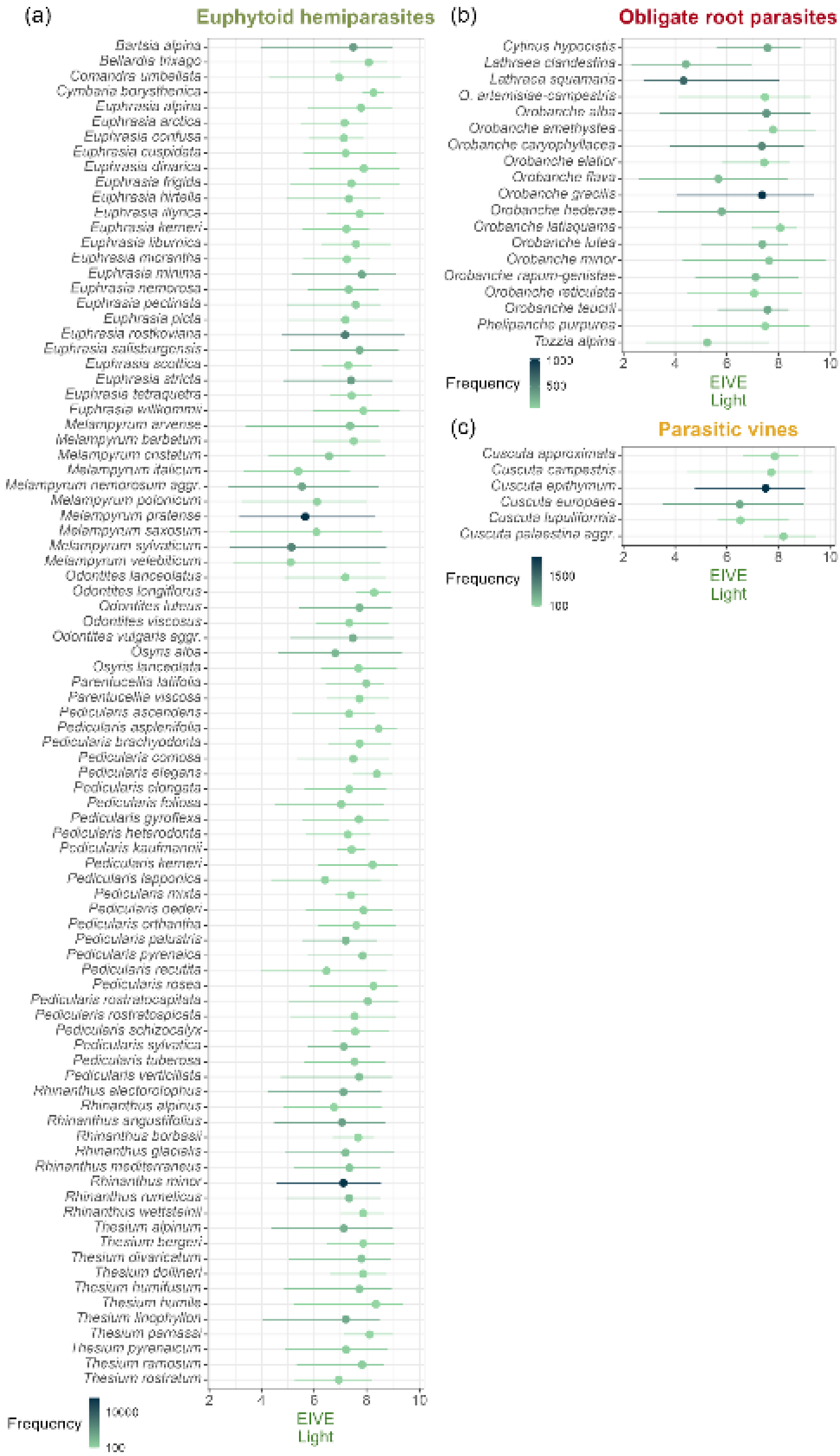

Fig. S10.13. Ranges (lines) and optima (full points) of parasitic plant species along the EIVE Light for a) euphytoid hemiparasites, b) obligate root parasites, and c) parasitic vines. The ranges were defined as intervals covering 95% of cover-weighted presences of given species while the optima were defined as cover-weighted average.

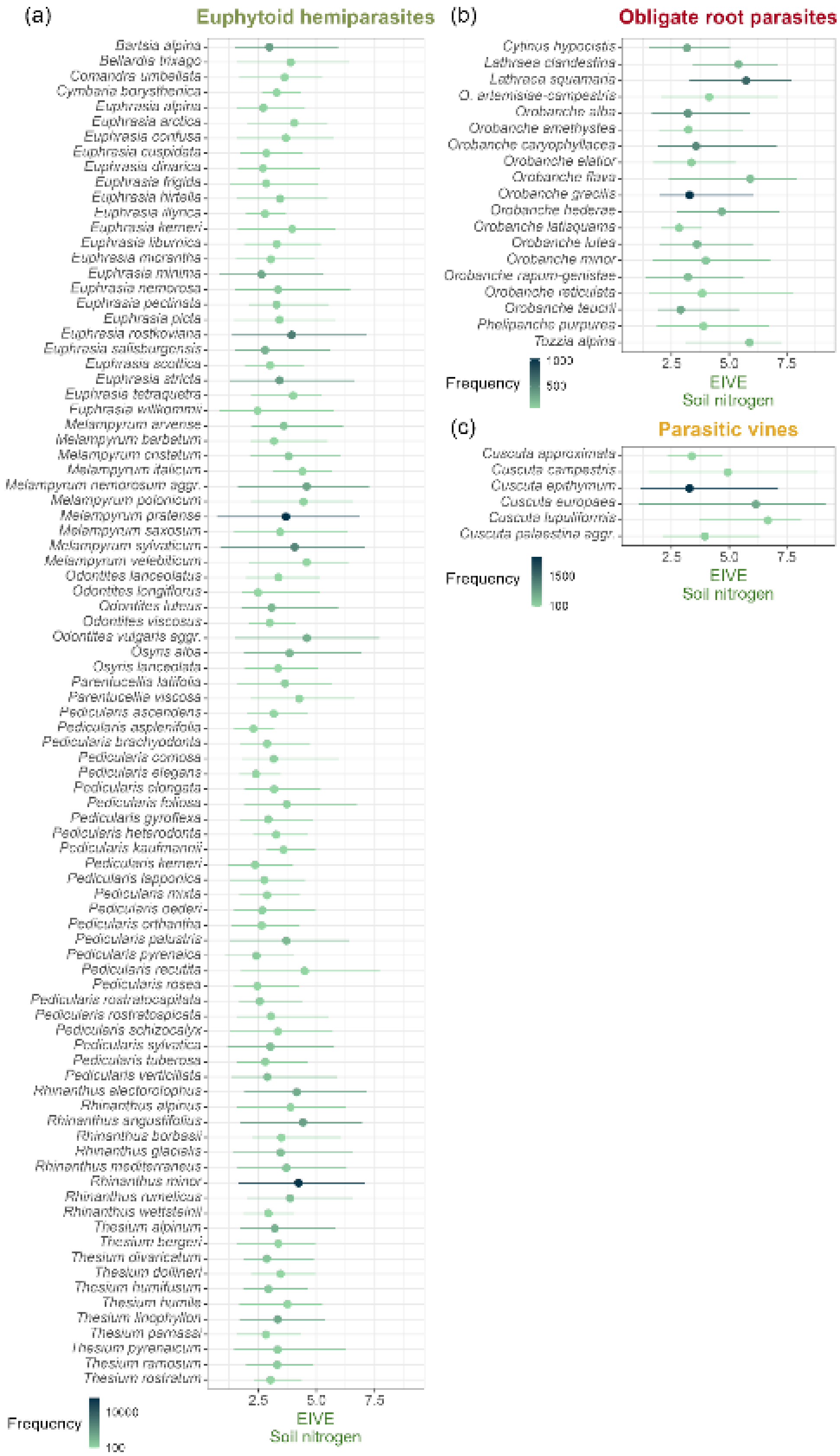

Fig. S10.14. Ranges (lines) and optima (full points) of parasitic plant species along the EIVE Soil nitrogen for a) euphytoid hemiparasites, b) obligate root parasites, and c) parasitic vines. The ranges were defined as intervals covering 95% of cover-weighted presences of given species while the optima were defined as cover-weighted average.

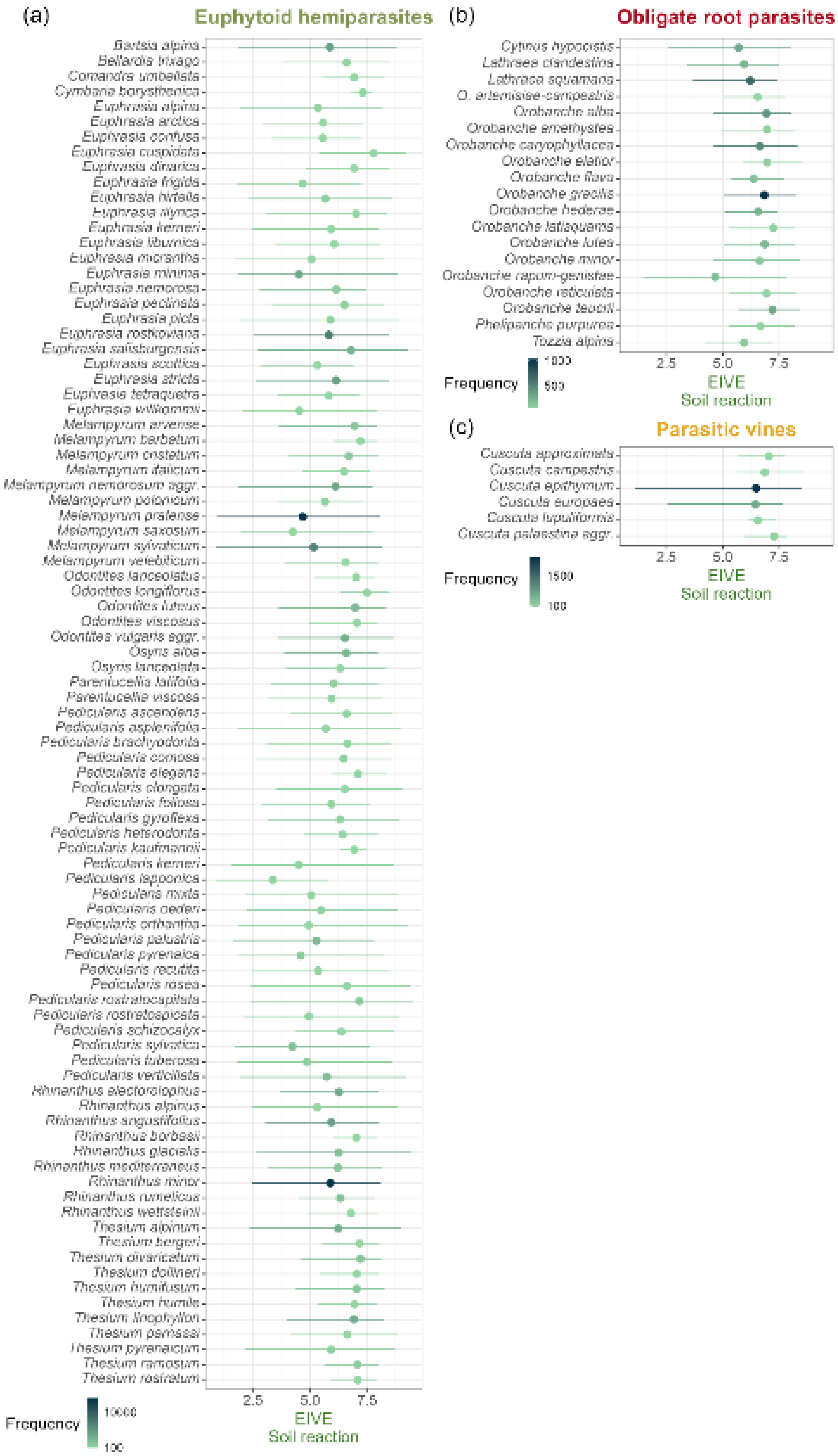

Fig. S10.15. Ranges (lines) and optima (full points) of parasitic plant species along the EIVE Soil reaction for a) euphytoid hemiparasites, b) obligate root parasites, and c) parasitic vines. The ranges were defined as intervals covering 95% of cover-weighted presences of given species while the optima were defined as cover-weighted average.

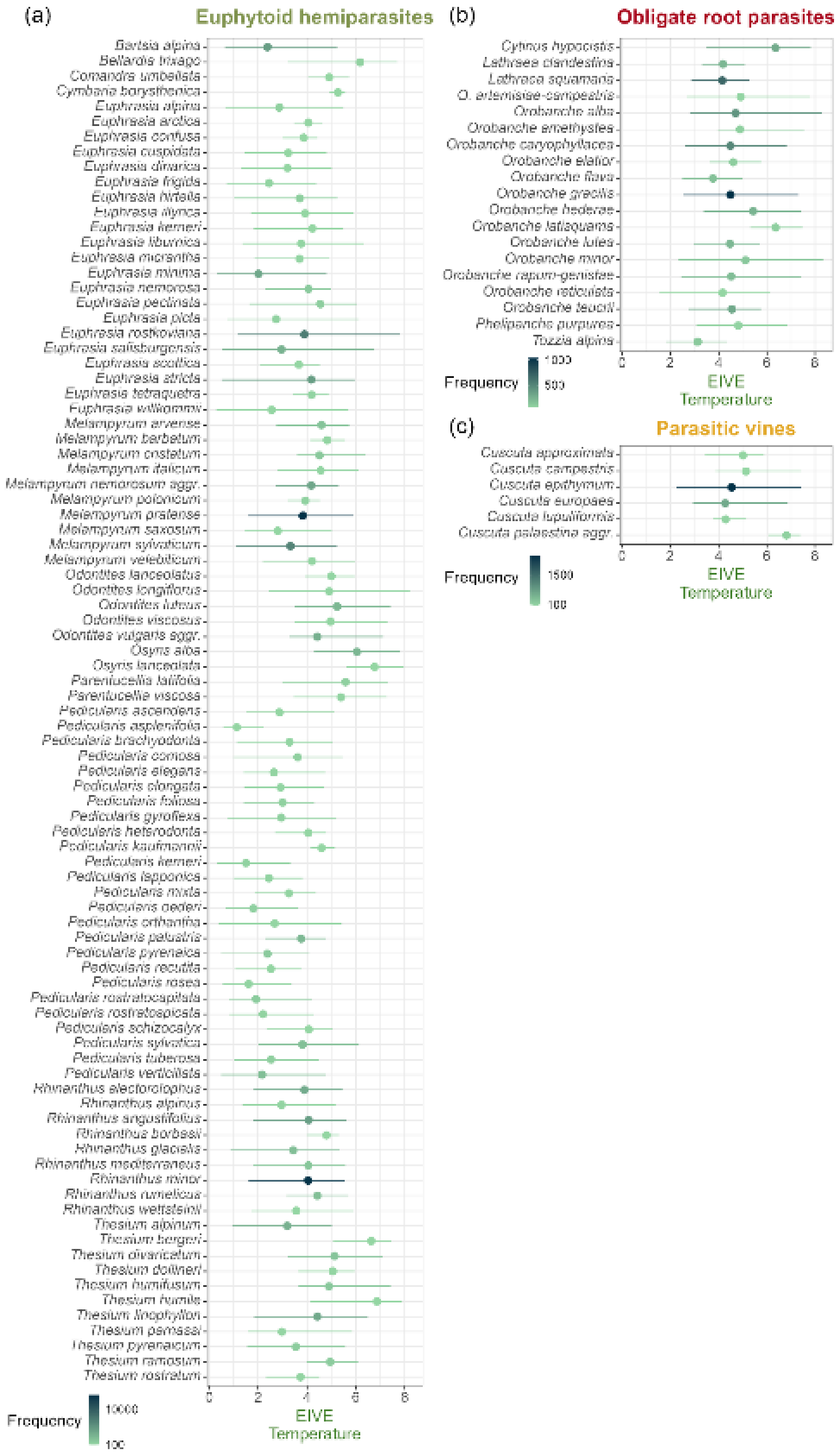

Fig. S10.15. Ranges (lines) and optima (full points) of parasitic plant species along the EIVE Temperature for a) euphytoid hemiparasites, b) obligate root parasites, and c) parasitic vines. The ranges were defined as intervals covering 95% of cover-weighted presences of given species while the optima were defined as cover-weighted average.
